# Arylazo Sulfones as Nonionic Visible-Light Photoacid Generators

**DOI:** 10.1021/acs.joc.2c01248

**Published:** 2022-07-22

**Authors:** Lorenzo Di Terlizzi, Angelo Martinelli, Daniele Merli, Stefano Protti, Maurizio Fagnoni

**Affiliations:** † PhotoGreen Lab, Department of Chemistry, University of Pavia, Viale Taramelli 12, 27100 Pavia, Italy; ‡ Department of Chemistry, University of Pavia, Viale Taramelli 12, 27100 Pavia, Italy

## Abstract

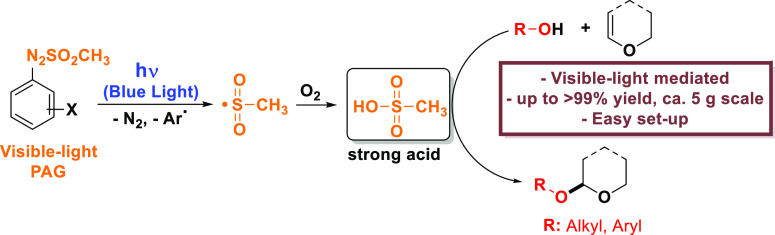

The selective visible-light-driven generation of a weak acid (sulfinic acid, in nitrogen-purged solutions) or a strong acid (sulfonic acid, in oxygen-purged solutions) by using shelf-stable arylazo sulfones was developed. These sulfones were then used for the green, smooth, and efficient photochemical catalytic protection of several (substituted) alcohols (and phenols) as tetrahydropyranyl ethers or acetals.

## Introduction

**Scheme 1:**
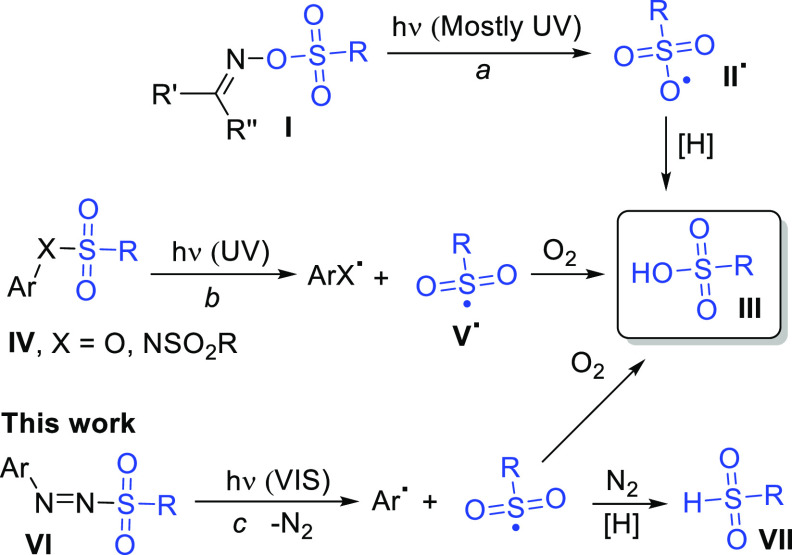
Common Pathways for the Photorelease of Sulfonic Acids

Photoacid generators (PAG) are compounds able to release an acid upon irradiation. Their use in lithography and printing processes is well established, albeit new applications (including cationic polymerizations, construction of microfluidic systems, photocurable coatings, and 3D printing) have emerged in the last three decades. In view of these premises, the design of new classes of PAGs is now a hot topic.^1^ It is widely accepted that PAGs may be classified into two classes: ionic and nonionic derivatives. The former ones are salts having an onium cation such as aryl diazonium,^2^ diaryl halonium,^3−7^ triaryl sulfonium,^7−10^ and triaryl phosphonium,^11−13^ with a halide complex anion (BF_4_^–^, SbF_6_^–^, AsF_6_^–^, PF_6_^–^, or RSO_3_^–^) as the counterion, which exhibit an excellent thermal stability but a narrow wavelength range of absorption and limited solubility in organic media.^1g^ In contrast, nonionic PAGs have found several applications mainly as polymer initiators due to their good solubility in a wide range of organic solvents and polymer matrixes. Among the different nonionic PAGs tested,^1,14,15^ compounds containing a sulfonyl moiety are appealing since they released strong sulfonic acids mostly via generation of the sulfonyloxy radical (**II**^**•**^, Scheme 1 path a) that in turn abstracts a hydrogen atom from the medium. A typical case is the photolysis of α-sulfonyloxy ketones^16,17^ or more recently that of imino-sulfonate derivatives (**I**)^18,19^ that have been employed for the cationic ring-opening polymerization of ε-caprolactone.^20^ An alternative approach involves the release of a sulfonyl radical (**V**^**•**^) in the presence of oxygen. Various compounds have been successfully investigated as “caged sulfonyl radicals”^21^ including benzylic sulfonyl compounds,^22^ aryl sulfonates (**IV**, X = O, Scheme 1 path b),^23^ and even *N*-arylsulfonimides (**IV**, X = NSO_2_R) which were able to generate up to 2 equiv of acid per equivalent of PAG.^24^ Most of the PAGs releasing the sulfonic acid, however, are active only in the UV region.

Nevertheless, there is an interest in the development of visible-light PAGs for applications in 3D printing systems, photocurable adhesives, and incorporation in photoresists sensitive to 436 nm light (the so-called g-line^25^). Visible-absorbing sulfonium salts^26^ BODIPY^27^ and especially photochromic-based derivatives^28^ have been devised accordingly. As part of our ongoing interest in the applications of colored dyedauxiliary-group-bearing molecules, we focused on arylazo sulfones^29^ (**VI**, Scheme 1 path c) as visible-light-active PAGs.

**Figure 1:**
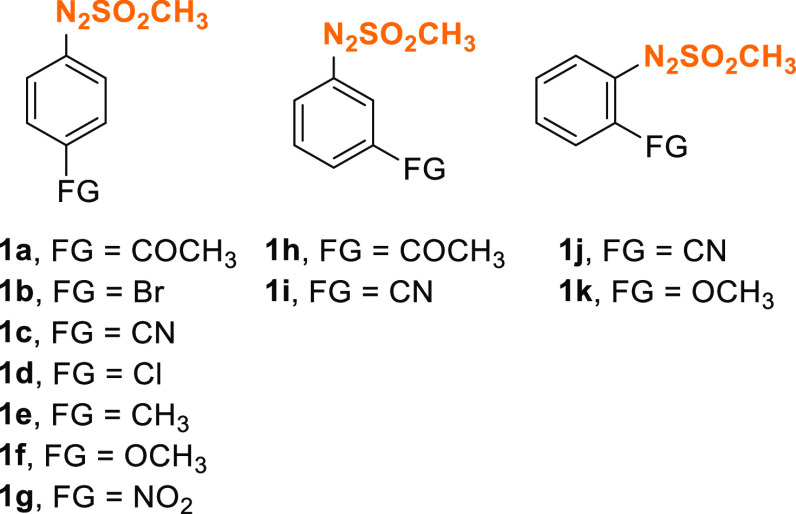
Arylazo sulfones tested in this work.

A dyed auxiliary group is a moiety which, once tethered to a molecule, makes the adduct colored and photoreactive.^29−31^ As an example, the irradiation of arylazo sulfones leads to the homolytic cleavage of the N–S bond forming an aryldiazenyl radical^32^ and a methansulfonyl radical. The latter species can generate sulfinic acid (**VII**) in deoxygenated conditions (Scheme 1) by hydrogen abstraction or methanesulfonic acid (**III**, R = Me) in an oxygen-saturated solution.^23,24^ Furthermore, arylazo sulfones exhibited a satisfactory thermal stability (some derivatives decompose over 145 °C)^30−32^ and an excellent solubility in a wide range of organic media.^30^ In view of these premises, we focused on a set of shelf-stable, easy to synthesize arylazo sulfones having a substituent in the *para* (**1a**–**g**), *meta* (**1h**–**i**), or *ortho* (**1j**–**k**) position (Figure 1). Indeed, the generation of PTSA p-toluenesulfonic acid) from the photolysis of phenylazo-*p*-tolyl sulfone was first observed in the early 1970s,^33^ but a detailed investigation of the acid release and its possible application was lacking.

## Results

Arylazo sulfones **1a**–**k** have been easily prepared from the corresponding anilines (see the Experimental Section). The UV–vis spectra of **1a**–**k** showed an intense absorption band in the UV region (λ_max_ = 280–315 nm) and a further band in the visible-light region (λ_max_ = 412–437 nm) except for **1g** and **1k**, which had a maximum centered at 395 nm (Table S1).

Preliminary irradiation experiments (λ = 456 nm) were carried out on a 2.5 × 10^–2^ M solution of compound **1d** in argon-purged acetonitrile. The reaction has been monitored evaluating both the consumption of **1d** and the amounts of photoproduct(s) obtained after irradiation (Table S2). A total consumption of the arylazo sulfone occurred after 3 h irradiation with chlorobenzene **2d** the only photoproduct detected. We then determined the disappearance quantum yields (Φ_–1_) of **1a**–**k** upon irradiation in oxygen-purged solutions along with the yields of photoproducts obtained and the amount (and the nature) of the acid release (Table 1). The Φ_–1_ values were quite low, not exceeding 0.05, with the *p*-methyl (**1e**) and *p*-nitro (**1g**) derivatives being the most photoreactive. Two main products were formed in the reaction, viz. the dediazosulfonylated derivatives **2a**–**g**^30^ and phenols **3a**–**k**,^34a^ and the latter mainly formed with sulfones bearing electron-withdrawing groups (Table 1). The trapping of aryl radicals by molecular oxygen and the subsequent generation of phenol has been widely reported in the literature.^34^ The low yields of compounds **3e**,**f** and **3j**,**k** may be due to the low stability of such compounds under the tested conditions that allow for a further oxidation to the corresponding quinones (vide infra).

**Table 1:**
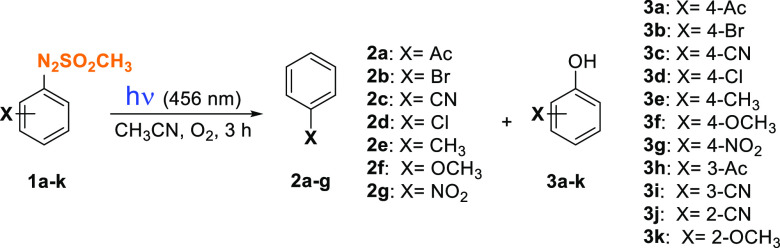
Photoreactivity of Arylazo Sulfones (**1a**–**k**) Irradiated in Oxygen-Purged Solutions^a^^,^^b^

azosulfone	Φ_–1_^b^^,^^c^	**2a**–**g**^d^ (%)	**3a**–**k**^d^ (%)	H^+^^e^ (% yield)	MeSO_3_H^f^ (% yield)
**1a**	0.01	0	52	81	44
**1b**	0.02	5	25	74	59
**1c**	0.01	5	70	94	41
**1d**	0.02	10	71	98	95
**1e**	0.05	6	0	86	78
**1f**	0.02	20	0	78	61
**1g**	0.05	15	69	82	75
**1h**	0.02	0	71	70	58
**1i**	0.02	9	64	59	45
**1j**	0.02	14	2	86	73
**1k**	0.02	4	8	87	87

a Conditions: an oxygen-purged acetonitrile solution of **1a**–**k** (2.5 × 10^–2^ M) was irradiated with a 40 W Kessil Lamp with emission centered at 456 nm for 3 h until the total consumption of the substrate.

b The consumption of **1a**–**k** was determined through HPLC analysis.

c Disappearance quantum yields (Φ_–1_) were measured on a Argon-purged 10^–2^ M acetonitrile solution of the chosen arylazo sulfone (λ= 456 nm, 1 × 40 W Kessil lamp).

d Yields of products were determined through GC analysis.

e Determined by potentiometric titration with a solution of NaOH 0.1 M.

f Determined through IC.

The acidity of the irradiated solutions was evaluated by means of potentiometric titrations with NaOH 0.1 M solution by diluting the sample with 50 mL of deionized water (see Figure 2 as a representative example and Figures S1–S11 for the remaining titrations).

**Figure 2:**
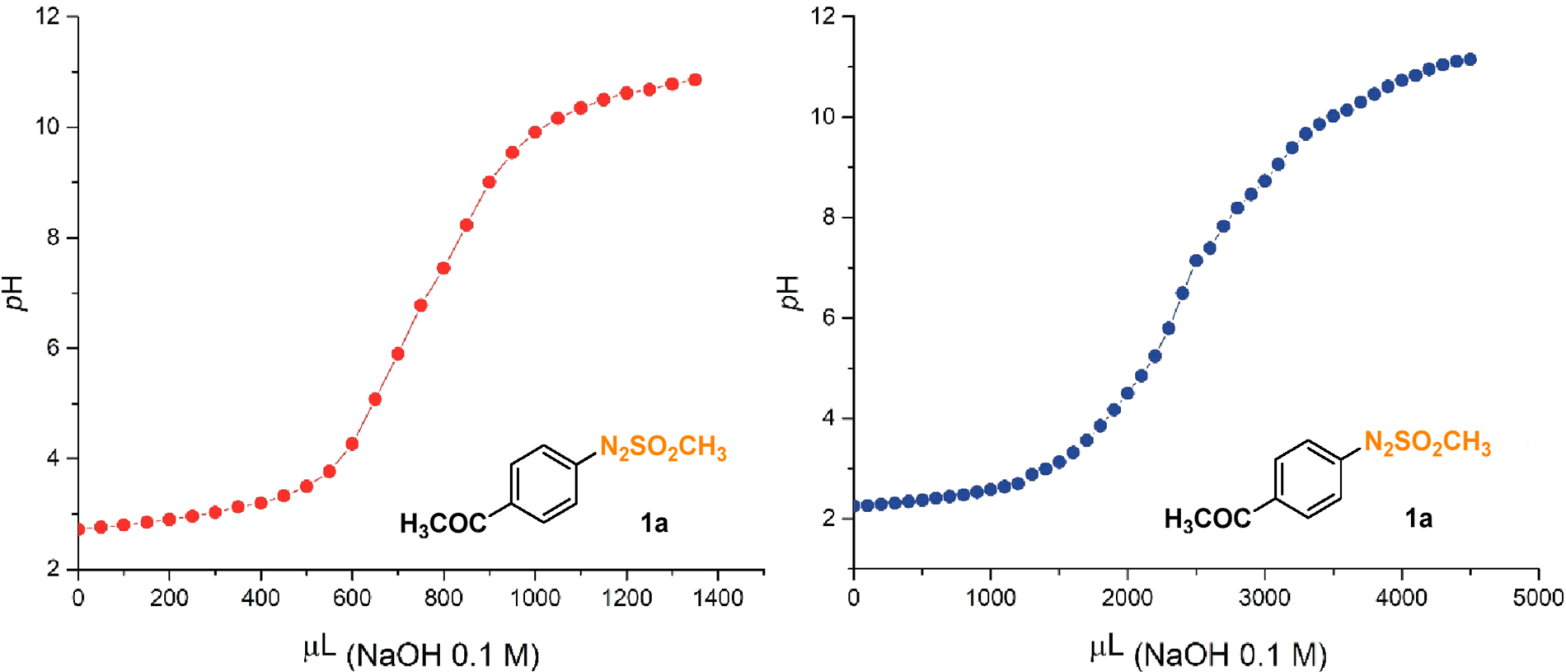
Potentiometric titration of 10 mL of a 2.5 × 10^–2^ M solution of **1a** irradiated in MeCN for 3 h (red, titration of the argon-purged solution; blue, titration of the oxygenated solution).

All of the substrates studied exhibited a good to excellent acid release (up to 95%) with methanesulfonic acid as the only species responsible for the acidity of the media as detected by IC analysis. As can be seen from Table 1, in some cases (**1d**–**f**, **1k**) the amount of total H^+^ found is in good agreement with the amount of methanesulfonic acid measured, while in other cases (e.g., **1a**, **1c**) the amount of H^+^ found is significantly higher. We also reasoned that the phenols generated in solution could be responsible of the remaining acid contribution, but potentiometric titration of 10 mL of a 2.5 × 10^–2^ M solution of **3a** or **3b** (Figure S12) proved that such compounds were not titrated during the measurement of the total acidity released from the irradiated arylazo sulfones. This is also confirmed by the p*K*_a_ values of phenols **3a**–**k** (Table S3). GC–MS analyses of the head space of the irradiated solutions of **1a** and **1f** (Figures S13 and S14) showed the presence of sulfur dioxide that passes undetected by IC and may account for the lack of sulfur-containing acids. Moreover, the formation of quinone-like products (by oxidation of **3a**–**k** as evidenced by the brownish color of the solution after irradiation) known to release protons possibly gives an alternative explanation.^35^

Irradiation experiments on selected arylazo sulfones (**1a**–**g**) were likewise carried out under argon-purged conditions (Table 2), forming **2a**–**g** as the exclusive products. Weak methanesulfinic acid accounted for most of the acidity liberated (in variable amounts) as detected by IC.

**Table 2:**
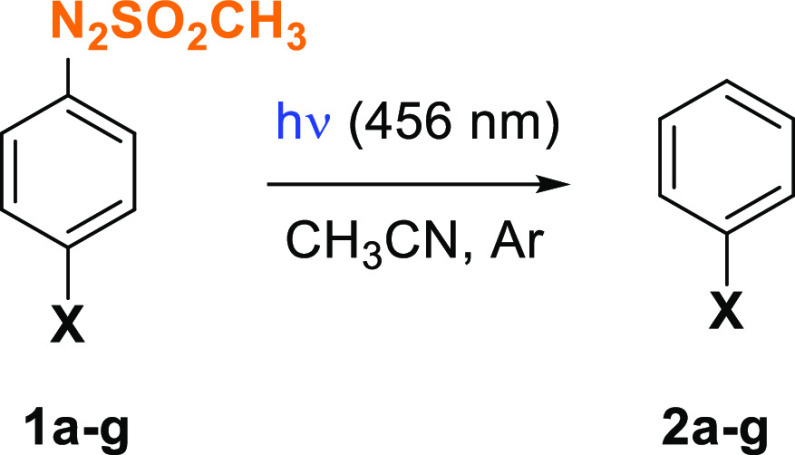
Photoreactivity of Arylazo Sulfones (**1a**–**g**) Irradiated in Argon Purged Solutions^a^^,^^b^

azosulfone	**2a**–**g**^c^	H^+^^d^	MeSO_2_H^e^ (%)
**1a**	44	76	52
**1b**	51	86	69
**1c**	51	69	67
**1d**	63	31	31
**1e**	76	72	71
**1f**	39	21	21
**1g**	75	42	41

a Conditions: an argon-purged acetonitrile solution of **1a**–**g** (2.5 × 10^–2^ M) was irradiated with one 40 W Kessil Lamp with emission centered at 456 nm for 3 h until the total consumption of the arylazo sulfone.

b The consumption of **1a**–**g** was determined by HPLC analysis.

c Yields of **2a**–**g** were determined by GC analysis.

d Determined by potentiometric titration with a solution of NaOH 0.1 M.

e Determined through IC.

With these results in hand, we decided to test arylazo sulfones in the role of PAGs for the photochemical catalytic protection of alcohols **4a**–**m** (see Figure S15) by reaction with 3,4-dihydro-2*H*-pyran **5a** and vinyl ethyl ether **5b**. The protection of benzyl alcohol **4a** as the tetrahydropyranyl ether was taken as a model reaction and optimized (see Table S4). The process was completed after 30 min irradiation in an air-equilibrated acetonitrile solution, employing only 0.5 mol % of an arylazo sulfone. All of the sulfones tested (**1d**–**f**) were effective in promoting the synthesis of THP ether **6** with **1e** being the derivative that led to an almost quantitative yield (Table 3). The reaction did not take place in the absence of **1e** and/or visible light (Table S4). Interestingly, the slow release of acid was beneficial to the reaction since the addition of PTSA or methanesulfonic acid (0.5 mol %) to a mixture of **4a** and **5a** did not lead to the desired acetal after 30 min (Table S4). Compound **6** was isolated in >99% yield even on a 25 mmol scale (4.80 g) and using natural sunlight (2 h) as the light source (Figure S16 and Table 3).

**Table 3:**
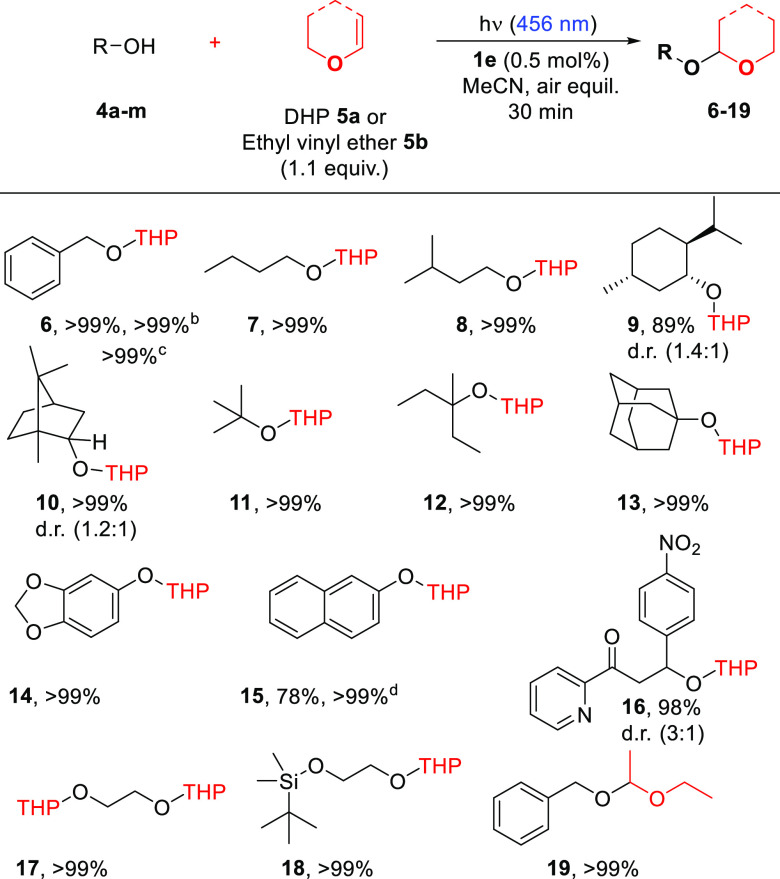
Photochemical-Catalyzed Protection of Alcohols **4a**–**m**.^a^

a Conditions: a solution containing the alcohol **4a**–**m** (5 mmol, 1 equiv), a vinyl ether **5a,b** (5.5 mmol, 1.1 equiv), and **1e** (0.5 mol %) in 4 mL of MeCN was irradiated under air-equilibrated conditions with one 40 W Kessil lamp (emission centered at 456 nm). Isolated yield shown.

b Reaction carried out on 25 mmol scale.

c Upon 2 h sunlight exposure.

d Yield determined by GC analysis.

With these promising results in our hands, different alcohols have been used, and all of them were successfully protected as tetrahydropyranyl ethers. The protocol was efficiently extended to primary alcohols (to give **7** and **8**), secondary (**9** and **10**), and tertiary alcohols (compounds **11**–**13**) even when particularly congested such as adamantan-1-ol **4h** and borneol **4e**. In all cases, an almost quantitative formation of the protected adduct occurred. Protection of phenol **4i** and naphthol **4j** to give ethers **14** and **15** was likewise feasible (Table 3). The presence of other functional groups (see the preparation of **16**) did not hamper the protection event. In the case of ethylene glycol **4l**, the protection of both OH groups smoothly took place by employing 2.2 equiv of **5a**. The mild conditions used allowed us to efficiently protect dimethyl-*tert*-butylsilyl alcohol **4m**, which bears a functional group sensitive to acidic conditions for the desymmetrization of ethylene glycol. Finally, to prove the versatility of this protocol, **4a** was likewise protected by using ethyl vinyl ether **5b** to afford product **19**, again quantitatively.

To verify if the irradiation was also crucial for the entire duration of the reaction, we performed some experiments on the synthesis of **6** at different reaction times: 5, 15, and 30 min (Table S5). It was apparent that the reaction proceeded even in the dark (albeit more slowly) when the reaction mixture was covered with aluminum foil after only 5 min irradiation and the mixture was maintained at room temperature for 30 min (the yield of **6** was 84% vs >99% under continuous photolysis).

## Discussion

**Scheme 2:**
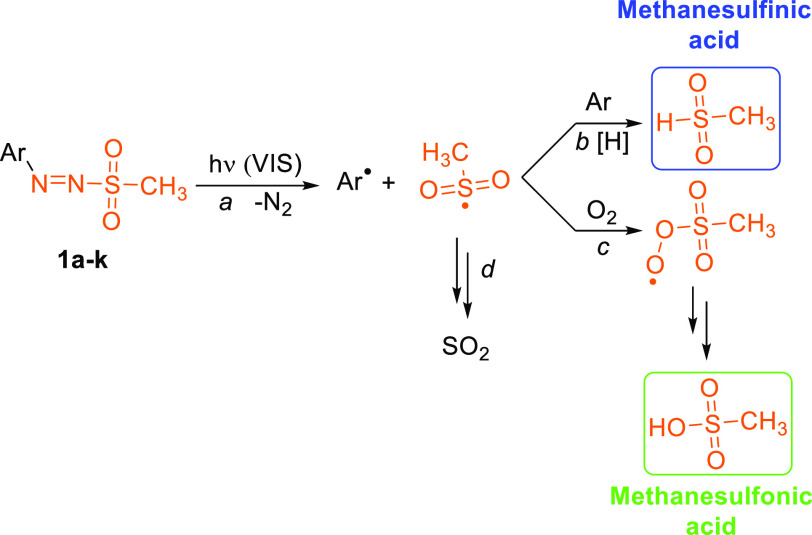
Photoacid Release from Arylazo Sulfones in Argon- and Oxygen-Purged Solutions

The photochemistry of arylazo sulfones was so far studied only for synthetic purposes^30−32,34,36^ in free-radical polymerization^37^ or for the functionalization of gold^38^ and graphene^39^ surfaces. This work clearly demonstrates that these compounds may be promising derivatives for the visible-light release of protons. As previously stressed, the first photochemical event is the homolytic cleavage of the N–S bond followed by nitrogen loss to form (apart an aryl radical) a sulfonyl radical (Scheme 2, *path a*), which acts as the source of acid whose strength depends on the conditions used. The photolysis of **1a**–**k** in argon-purged acetonitrile led to the formation of weak methanesulfinic acid in fair amounts (Table 2) via hydrogen atom abstraction from the media as already reported (path b).^23,24^ However, the amount (up to quantitative for **1d**) and the strength of the acid released improved by shifting to oxygenated conditions where MeSO_3_H is the only species that accounted for the overall acidity (Table 1). Here, the addition of the sulfonyl radical to oxygen took place (Scheme 2, path c).^23,24^ The lack of sulfur-containing derivatives, evidenced in both deaerated and oxygenated conditions, was assigned to sulfur dioxide release by the sulfonyl radical (path d) as proven by head space GC experiments (Figures S13 and S14)

As a matter of fact, the BDE of the C–S bond in the methanesulfonyl radical was estimated to be around 18 kcal mol^–1^ in the gas phase,^40^ making the α-scission feasible.^41^

The low quantum yield in the acid release (<0.05, Table 1) did not hamper the synthetic applications of the examined PAGs, as demonstrated by the protection of alcohols as THP ethers. The formation of these ethers starting from 3,4-dihydro-2*H*-pyran (DHP) is commonly used to protect hydroxyl functional groups.^42,43^ This reaction may be carried out by using a plethora of protocols^44,45^ but some drawbacks still remain. Thus, the acid catalysts mostly used may induce the formation of polymeric byproducts derived from DHP or may be incompatible with other acid-sensitive functional groups. The reaction may suffer upon the use of elevated temperature, long reaction times, harmful solvents and a huge excess of DHP (up to 2 equiv). Dedicated catalysts have been designed, but they can be expensive, moisture sensitive, freshly prepared just prior to use, and based on metal species.^44a−44i^ Representative recent catalytic systems used for the tetrahydropyranylation of alcohols and phenols are 10 mol % pyridinium *p*-toluensulfonate (DCM as solvent, 2 equiv of DHP used),^44a^ diphenylamine-terephthalaldehyde resin *p*-toluenesulfonate (1.5 equiv of DHP),^44b^ silica-gel-supported aluminum chloride (complex synthesis of the resin, dichloroethane as solvent),^44c^ copper(II) chloride–acetic acid (unsatisfactory protection of phenols),^44d^ 5 mol % bismuth(III) nitrate pentahydrate (heavy metal used),^44e^ and Keggin heteropolyacids (MO_6_, M = Mo(VI), W(VI)) (2 equiv of DHP).^44f^

Interestingly, whereas the photochemical deprotection of protected functional groups is sparsely used in synthesis^42^ the protection protocol is quite uncommon, and a single case on the use of dichloroanthraquinone (DCQ) to protect alcohols as THP ethers under photochemical conditions has been reported.^45^ The reaction proceeded in a good fashion under visible light but employed a large amount of PAG (5 mol %), and DCM is the chosen solvent.^45^

The use of arylazo sulfones as caged protons has several advantages. These compounds are not known to engage undesired secondary reactions with most functional groups. Arylazo sulfones can be used in very low amounts (0.5 mol %), and the reaction is complete after only 30 min employing an easy setup (a simple glass vessel must be used). The reaction is promoted by both visible and natural sunlight to maintain the same yield (always close to quantitative) and has been proven to be efficient with several types of alcohols and phenols, even the hindered ones and with alcohols bearing an acid-sensitive functional group (e.g., **18**).

As for the mechanism, the slow acid release is the key to the success of the reaction (a sulfonic acid added in one portion is detrimental; see Table S4). The experiments reported in Table S5 evidenced that the reaction may proceed even in the dark (albeit slowly) after a short irradiation time, confirming that the methanesulfonic acid liberated after the first instance of the reaction could be sufficient to complete the process. However, the reaction kept under continuous irradiation for 30 min sped up the tetrahypyranylation event.

## Conclusions

Arylazo sulfones are an intriguing class of shelf-stable and colored compounds for the visible-light photochemical catalytic release of acids. The different behavior of these sulfones in different media allowed us to tune the strength of the acid release (from weak sulfinic acid to strong sulfonic acid). The role of arylazo sulfones as PAG has been exploited in the acid-catalyzed protection of alcohols as acetals under mild conditions and upon either visible or (natural) solar light irradiation. The latter process did not require any dedicated apparatus, harsh conditions, or delicate catalysts in order to take place.

## Experimental Section

### General Information

^1^H and ^13^C NMR spectra were recorded on a 300 or 75 MHz spectrometer, respectively. The attributions were made on the basis of ^1^H and ^13^C NMR experiments; chemical shifts are reported in ppm downfield from TMS. GC analyses were performed using a HP SERIES 5890 II equipped with a fire ion detector (FID, temperature 350 °C). Analytes were separated using a Restek Rtx-5MS (30 m × 0.25 mm × 0.25 μm) capillary column with nitrogen as a carrier gas at 1 mL min^–1^. The injector temperature was 250 °C. The GC oven temperature was held at 80 °C for 2 min, increased to 250 °C by a temperature ramp of 10 °C min^–1^, and held for 10 min. The potentiometric titrations were carried out using a water solution of NaOH 0.1 M and a METLER-TOLEDO glass pHmeter. Ion chromatography analyses were performed by means of a Dionex GP40 instrument equipped with a conductometric detector (Dionex 20 CD20) and an electrochemical suppressor (ASRS Ultra II, 4 mm) by using the following conditions: chromatographic column IONPAC AS23 (4 mm × 250 mm), guard column IONPAC AG12 (4 mm × 50 mm), eluent: NaHCO_3_ 0.8 mm+Na_2_CO_3_ 4.5 mm, flux: 1 mL min^–1^; current imposed at detector: 50 mA. Commercially available sodium methanesulfinate and methanesulfonic acid were used as standards.

### General Procedure for the Synthesis of Arylazo Sulfones

Arylazo sulfones **1a**–**k** were previously synthesized and fully characterized by our research groups^46^ by the following procedure: diazonium salts^47^ were freshly prepared prior to use from the corresponding anilines and purified by dissolution in acetonitrile and precipitation by addition of cold diethyl ether. To a cooled (0 °C) suspension of the chosen diazonium salt (1 equiv, 0.3 M) in CH_2_Cl_2_ was added sodium methanesulfinate (1.2 equiv) in one portion. The temperature was allowed to rise to room temperature, and the solution stirred overnight. The resulting mixture was then filtered, and the obtained solution was evaporated to afford the desired arylazo sulfone. The crude product was finally dissolved in CH_2_Cl_2_ and precipitated by addition of cold *n*-hexane.^46^ Arylazo sulfone **1k** was synthesized starting from the corresponding diazonium salt, prepared following a known procedure.^46^ Alcohols **4a**–**m** were commercially available except **4k**(48) and **4m**(49) which were prepared as previously described.

### General Procedure for the Photoinduced Protection of Alcohols

A Pyrex glass vessel was charged with the chosen alcohol (**4a**–**m**, 5 mmol, 1 equiv, 1.25 M), the selected vinyl ether (**5a,b**, 5.5 mmol, 1.1 equiv, 1.375 M), and a catalytic amount of arylazo sulfone **1e** (0.025 mmol, 0.5 mol %) in 4 mL of acetonitrile. The so-formed mixture was irradiated for 30 min by using the EvoluChem apparatus equipped with one 40 W Kessil lamp (emission centered at 456 nm) placed 3 cm above the reaction vessel. The solvent was eliminated in vacuo, and the residue was purified by silica gel column chromatography (cyclohexane–ethyl acetate mixture as eluant).

#### 2-(Benzyloxy)tetrahydro-2*H*-pyran (**6**)

From 5 mg (0.025 mmol, 0.5 mol %) of **1e**, 744 μL of **5a** (5.5 mmol, 1.1 equiv), and 520 μL of **4a** (5 mmol, 1 equiv). Purification was carried out on a silica gel chromatographic column (eluant: neat cyclohexane) to afford 960.0 mg of **6** (>99% yield, colorless liquid). Spectroscopic data were in accordance with the literature.^50^^1^H NMR (300 MHz, acetone-*d*_6_): δ 7.51–7.17 (m, 5H), 4.82–4.71 (m, 2H), 4.52 (d, *J* = 12.1 Hz, 1H), 3.95–3.50 (m, 2H), 1.93–1.52 (m, 6H). ^13^C{^1^H} NMR (75 MHz, acetone-*d*_6_): δ 139.8, 129.0, 128.4, 128.0, 98.4, 69.2, 62.2, 31.3, 26.3, 20.0. The reaction was scaled up, starting from 25 mg (0.125 mmol, 0.5 mol %) of **1e**, 3.7 mL of **5a** (27.5 mmol, 1.1 equiv), and 2.6 mL of **4a** (25 mmol, 1 equiv) in 10 mL of acetonitrile. Purification was carried out by silica gel chromatography (eluant: neat cyclohexane) to afford 4.80 g of **6** (>99% yield, colorless liquid).

#### Sunlight-Promoted Synthesis of **6**

A Pyrex glass vessel was charged with **1e** (0.025 mmol, 0.5 mol %), benzyl alcohol **4a** (5 mmol, 1 equiv, 1.25 M), and **5a** (5.5 mmol, 1.1 equiv, 1.375 M) in 4 mL of acetonitrile. The mixture was placed on a window ledge of the University of Pavia (45°11′31″ N, 9°09′33″ E, 77 above sea level, 08/04/2022, 10:00 am) during a sunny day, and the reaction was monitored through GC analysis. After 2 h, the reaction was completed to afford 960 mg of **6** (5 mmol, > 99% yield). (See Figure S16.)

#### 2-Butoxytetrahydro-2*H*-pyran (**7**)

From 5 mg (0.025 mmol, 0.5 mol %) of **1e**, 744 μL of **5a** (5.5 mmol, 1.1 equiv), and 457 μL of **4b** (5 mmol, 1 equiv). Purification was carried out by silica gel chromatographic column (eluant: neat cyclohexane) to afford 850.0 mg of **7** (>99% yield, colorless liquid). Spectroscopic data were in accordance with the literature.^51^^1^H NMR (300 MHz, acetone-*d*_6_): δ 4.56 (t, *J* = 3.5 Hz, 1H), 3.84–3.66 (m, 2H), 3.49–3.31 (m, 2H), 1.46 (s, 12H), 0.94 (t, *J* = 7.3 Hz, 3H). ^13^C{^1^H} NMR (75 MHz, acetone-*d*_6_): δ 99.1, 67.4, 62.1, 32.7, 31.5, 27.6, 26.5, 20.2, 14.3.

#### 2-(Isopentyloxy)tetrahydro-2*H*-pyran (**8**)

From 5 mg (0.025 mmol, 0.5 mol %) of **1e**, 744 μL of **5a** (5.5 mmol, 1.1 equiv), and 545 μL of **4c** (5 mmol, 1 equiv). Purification was carried out by silica gel chromatographic column (eluant: neat cyclohexane) to afford 915.0 mg of **8** (>99% yield, colorless liquid). Spectroscopic data were in accordance with the literature.^52^^1^H NMR (300 MHz, acetone-*d*_6_): δ 4.57 (t, *J* = 3.4 Hz, 1H), 3.86–3.71 (m, 2H), 3.52–3.34 (m, 2H), 1.86–1.47 (m, 9H), 0.95 (d, *J* = 6.6 Hz, 6H). ^13^C{^1^H} NMR (75 MHz, acetone-*d*_6_): δ 98.8, 66.0, 61.8, 39.5, 31.5, 26.4, 25.9, 23.2, 23.0, 20.1.

#### 2-((2-Isopropyl-5-methylcyclohexyl)oxy)tetrahydro-2*H*-pyran (**9**)

From 5 mg (0.025 mmol, 0.5 mol %) of **1e**, 744 μL of **5a** (5.5 mmol, 1.1 equiv), and 780.0 mg of **4d** (5 mmol, 1 equiv). Purification was carried out by silica gel chromatographic column (eluant: cyclohexane/ethyl acetate mixture) to afford 1.115 g of **9** (89% yield, colorless oil). Spectroscopic data were in accordance with the literature.^53^ The product was obtained as a diastereomeric mixture (1.4:1). ^1^H NMR (300 MHz, acetone-*d*_6_): δ 4.84–4.57 (m, 1H), 3.88 (dtd, *J* = 15.0, 7.5, 4.0 Hz, 1H), 3.53–3.27 (m, 2H), 2.44–2.08 (m, 2H), 1.91–1.01 (m, 12H), 0.96–0.87 (m, 7H), 0.80 (dd, *J* = 6.9, 4.7 Hz, 4H). ^13^C{^1^H} NMR (75 MHz, acetone-*d*_6_): δ 101.6, 95.3, 80.5, 74.6, 63.1, 62.8, 49.2, 44.5, 41.0, 35.4, 32.4, 32.3, 30.6, 26.4, 26.0, 23.9, 22.7, 21.5, 20.7, 16.7, 16.2.

#### 2-((1,7,7-Trimethylbicyclo[2.2.1]heptan-2-yl)oxy)tetrahydro-2*H*-pyran (**10**)

From 5 mg (0.025 mmol, 0.5 mol %) of **1e**, 744 μL of **5a** (5.5 mmol, 1.1 equiv), and 770 mg of **4e** (5 mmol, 1 equiv). Purification was carried out by silica gel chromatographic column (eluant: neat cyclohexane) to afford 1.19 g of **10** (>99% yield, colorless oil). Spectroscopic data were in accordance with the literature.^54^ The product was obtained as a diastereomeric mixture (1.2:1). ^1^H NMR (300 MHz, acetone-*d*_6_): δ 4.68–4.52 (m, 1H), 4.10–3.52 (m, 2H), 3.44 (ddd, *J* = 11.5, 4.1, 1.9 Hz, 1H), 2.24–2.01 (m, 2H), 1.88–1.47 (m, 9H), 1.28–1.16 (m, 2H), 0.95–0.81 (m, 9H). ^13^C{^1^H} NMR (75 MHz, acetone-*d*_6_): δ 101.5, 96.8, 84.7, 80.0, 62.3, 50.3, 49.7, 48.6, 48.2, 46.3, 46.2, 41.0, 38.5, 36.6, 32.3, 27.7, 26.8, 20.8, 20.4, 19.5, 14.5.

#### 2-*tert*-Butoxytetrahydro-2*H*-pyran (**11**)

From 5 mg (0.025 mmol, 0.5 mol %) of **1e**, 744 μL of **5a** (5.5 mmol, 1.1 equiv), and 478 μL of **4f** (5 mmol, 1 equiv). Purification was carried out by silica gel chromatographic column (eluant: neat cyclohexane) to afford 850.0 mg of **11** (>99% yield, colorless liquid). Spectroscopic data were in accordance with the literature.^55^^1^H NMR (300 MHz, acetone-*d*_6_): δ 4.83 (dd, *J* = 4.8, 2.8 Hz, 1H), 3.94–3.39 (m, 2H), 1.85–1.43 (m, 6H), 1.22 (s, 9H). ^13^C{^1^H} NMR (75 MHz, acetone-*d*_6_): δ 94.7, 74.8, 63.1, 33.8, 29.8, 27.0, 21.5.

#### 2-((3-Methylpentan-3-yl)oxy)tetrahydro-2*H*-pyran (**12**)

From 5 mg (0.025 mmol, 0.5 mol %) of **1e**, 744 μL of **5a** (5.5 mmol, 1.1 equiv), and 616 μL of **4g** (5 mmol, 1 equiv). Purification was carried out by silica gel chromatographic column (eluant: neat cyclohexane) to afford 942.0 mg of **12** (>99% yield, colorless liquid). Spectroscopic data were in accordance with the literature.^56^^1^H NMR (300 MHz, acetone-*d*_6_): δ 4.82–4.67 (m, 1H), 4.02–3.41 (m, 2H), 2.05–1.58 (m, 4H), 1.51 (ddd, *J* = 9.4, 6.1, 2.3 Hz, 6H), 1.15 (d, *J* = 3.9 Hz, 3H), 0.87 (q, *J* = 7.3 Hz, 6H). ^13^C{^1^H} NMR (75 MHz, acetone-*d*_6_): δ 93.6, 78.8, 63.5, 32.7, 31.5, 31.0, 25.7, 23.2, 21.0, 6.41, 6.27.

#### 2-((Adamantan-1-yl)oxy)tetrahydro-2*H*-pyran (**13**)

From 5 mg (0.025 mmol, 0.5 mol %) of **1e**, 744 μL of **5a** (5.5 mmol, 1.1 equiv), and 761 mg of **4h** (5 mmol, 1 equiv). Purification was carried out by silica gel chromatographic column (eluant: neat cyclohexane) to afford 1.18 g of **13** (>99% yield, colorless oil). Spectroscopic data were in accordance with the literature.^55^^1^H NMR (300 MHz, acetone-*d*_6_): δ 4.95 (dd, *J* = 4.8, 2.7 Hz, 1H), 3.93–3.38 (m, 2H), 2.13–1.42 (m, 21H). ^13^C{^1^H} NMR (75 MHz, acetone-*d*_6_): δ 92.8,73.7, 62.8, 43.8, 37.4, 33.5, 31.8, 26.7, 21.2.

#### 5-((Tetrahydro-2*H*-pyran-2-yl)oxy)benzo[*d*][1,3]dioxole (**14**)

From 5 mg (0.025 mmol, 0.5 mol %) of **1e**, 744 μL of **5a** (5.5 mmol, 1.1 equiv), and 690 mg of **4i** (5 mmol, 1 equiv). Purification was carried out by silica gel chromatographic column (eluant: cyclohexane/ethyl acetate mixture) to afford 1.24 g of **14** (>99% yield, slightly yellow oil). Spectroscopic data were in accordance with the literature.^57^^1^H NMR (300 MHz, acetone-*d*_6_): δ 6.65 (d, *J* = 8.3 Hz, 1H), 6.42 (d, *J* = 2.5 Hz, 1H), 6.29 (dd, *J* = 8.3, 2.5 Hz, 1H), 5.89 (s, 2H), 3.84 (d, *J* = 19.0 Hz, 1H), 3.60–3.22 (m, 2H), 1.69–1.47 (m, 6H). ^13^C{^1^H} NMR (75 MHz, acetone-*d*_6_): δ 153.5, 149.1, 141.4, 108.8, 107.2, 101.7, 98.7, 67.4, 31.4, 27.5, 26.3, 20.16.

#### 2-(Naphthalen-2-yloxy)tetrahydro-2*H*-pyran (**15**)

From 5 mg (0.025 mmol, 0.5 mol %) of **1e**, 744 μL of **5a** (5.5 mmol, 1.1 equiv), and 720 mg of **4j** (5 mmol, 1 equiv). Purification was carried out by silica gel chromatographic column (eluant: cyclohexane/ethyl acetate mixture) to afford 929.0 mg of **15** (78% yield, > 99% GC yield, yellow oil). Spectroscopic data were in accordance with the literature.^58^^1^H NMR (300 MHz, acetone-*d*_6_): δ 7.84 (dd, *J* = 8.0, 5.6 Hz, 3H), 7.61–7.28 (m, 4H), 5.62 (t, *J* = 3.2 Hz, 1H), 3.96–3.54 (m, 2H), 2.06–1.56 (m, 6H). ^13^C{^1^H} NMR (75 MHz, acetone-*d*_6_): δ 156.1, 135.8, 130.6, 130.3, 128.7, 128.1, 127.3, 124.9, 120.3, 111.6, 97.3, 62.6, 31.4, 26.2, 19.8

#### 3-(4-Nitrophenyl)-1-(pyridin-2-yl)-3-((tetrahydro-2*H*-pyran-2-yl)oxy)propan-1-one (**16**)

From 5 mg (0.025 mmol, 0.5 mol %) of **1e**, 744 μL of **5a** (5.5 mmol, 1.1 equiv), and 1360 mg of **4k** (5 mmol, 1 equiv). Purification was carried out by silica gel chromatographic column (eluant: cyclohexane/ethyl acetate mixture) to afford 1781.0 mg of **16** (98% yield, yellow oil). The product was obtained as a diastereomeric mixture (3:1). ^1^H NMR (300 MHz, acetone-*d*_6_): δ 8.86–8.67 (m, 4H), 8.52 (d, *J* = 16.2 Hz, 1H), 8.39–8.25 (m, 3H), 8.30–8.20 (m, 3H), 8.25–8.13 (m, 2H), 8.19–7.90 (m, 10H), 7.84–7.66 (m, 7H), 7.71–7.59 (m, 3H), 5.58 (dd, *J* = 8.3, 5.2 Hz, 2H), 5.45 (dd, *J* = 8.4, 4.6 Hz, 1H), 4.97 (t, *J* = 3.5 Hz, 1H), 4.44 (t, *J* = 3.2 Hz, 2H), 4.01–3.74 (m, 5H), 3.58–3.33 (m, 5H), 3.30–3.17 (m, 1H), 2.87 (s, 3H), 2.06 (qui, *J* = 2.2 Hz, 2H), 1.81–1.34 (m, 10H), 1.29 (s, 1H). ^13^C{^1^H} NMR (75 MHz, acetone-*d*_6_): δ 150.7, 150.2, 150.2, 150.1, 148.6, 141.8, 138.5, 138.3, 138.2, 130.5, 129.2, 128.6, 128.6, 128.5, 125.8, 125.0, 124.5, 124.2, 123.5, 122.4, 122.3, 100.4, 95.77, 75.9, 73.5, 62.9, 62.0, 46.6, 46.5, 27.6, 26.2, 26.2, 20.1, 19.5. HRMS (EI) *m*/*z*: [M + Na]^+^ Calcd for C_19_H_20_N_2_O_5_Na 379.1264; Found 379.1252.

#### 1,2-Bis((tetrahydro-2*H*-pyran-2-yl)oxy)ethane (**17**)

From 5 mg (0.025 mmol, 5 mol %) of **1e**, 1488 μL of **5a** (11 mmol, 2.2 equiv), and 280 μL of **4l** (5 mmol, 1 equiv). Purification was carried out by silica gel chromatographic column (eluant: cyclohexane/ethyl acetate mixture) to afford 1250.0 mg of **17** (>99% yield, yellow oil). Spectroscopic data were in accordance with the literature.^59^^1^H NMR (300 MHz, acetone-*d*_6_): δ 4.64 (q, *J* = 3.0 Hz, 2H), 3.83 (ddt, *J* = 11.2, 7.2, 3.7 Hz, 4H), 3.63–3.41 (m, 4H), 1.83–1.47 (m, 12H). ^13^C{^1^H} NMR (75 MHz, acetone-*d*_6_): δ 99.6, 67.7, 62.52, 32.0, 27.0, 20.6.

#### *tert*-Butyldimethyl(2-((tetrahydro-2*H*-pyran-2-yl)oxy)ethoxy)silane (**18**)

From 5 mg (0.025 mmol, 0.5 mol %) of **1e**, 744 μL of **5a** (5.5 mmol, 1.1 equiv), and 865 μL of **4m** (5 mmol, 1 equiv). Purification was carried out by silica gel chromatographic column (eluant: cyclohexane/ethyl acetate mixture) to afford 1.336 g of **18** (>99% yield, yellow oil). Spectroscopic data were in accordance with the literature.^60^^1^H NMR (300 MHz, acetone-*d*_6_): δ 4.64 (q, *J* = 3.0 Hz, 2H), 3.83 (ddt, *J* = 11.2, 7.2, 3.7 Hz, 4H), 3.63–3.41 (m, 4H), 1.83–1.47 (m, 12H). ^13^C{^1^H} NMR (75 MHz, acetone-*d*_6_): δ 99.6, 67.7, 62.52, 32.0, 27.0, 20.6.

#### ((1-Ethoxyethoxy)methyl)benzene (**19**)

From 5 mg (0.025 mmol, 0.5 mol %) of **1e**, 526 μL of ethyl vinyl ether **5b** (5.5 mmol, 1.1 equiv), and 520 μL of **4a** (5 mmol, 1 equiv). Purification was carried out by silica gel chromatographic column (eluant: neat cyclohexane) to afford 901 mg of **19** (>99% yield, colorless oil). Spectroscopic data were in accordance with the literature.^61^^1^H NMR (300 MHz, acetone-*d*_6_): δ 7.43–7.34 (m, 5H), 4.93 (dd, *J* = 41.3, 5.3 Hz, 1H), 4.73–4.60 (m, 2H), 3.83–3.42 (m, 2H), 1.38 (d, *J* = 5.3 Hz, 3H), 1.24 (t, *J* = 7.1 Hz, 3H). ^13^C{^1^H} NMR (75 MHz, acetone-*d*_6_): δ 140.4, 129.4, 128.7, 128.4, 100.4, 67.9, 61.6, 20.8, 16.3.

## Supporting Information Available

Experimental procedures, potentiometric titrations, and ^1^H and ^13^C NMR spectra of all new compounds (PDF)

## References

[ref1] aWan P.; Shukla D. Utility of acid-base behavior of excited states of organic molecules. Chem. Rev. 1993, 93, 571–584. 10.1021/cr00017a024.

[ref2] Smets G.; Aerts A.; Erum J. Photochemical Initiation of Cationic Polymerization and Its Kinetics. Polym. J. 1980, 12, 539–547. 10.1295/polymj.12.539.

[ref3] Crivello J. V.; Lam J. H. W. Diaryliodonium Salts. A New Class of Photoinitiators for Cationic Polymerization. Macromolecules 1977, 10, 1307–1315. 10.1021/ma60060a028.

[ref4] Pappas S. B.; Pappas B. C.; Gatechair L. R.; Schnabel W. Photoinitation of cationic polymerization. II. Laser flash photolysis of diphenyliodonium salts. J. Polym. Sci. Polym. Chem. Ed. 1984, 22, 69–76. 10.1002/pol.1984.170220107.

[ref5] Crivello J. V.; Lee J. L. Alkoxy-substituted diaryliodonium salt cationic photoinitiators. J. Polym. Sci. Polym. Chem. Ed. 1989, 27, 3951–3968. 10.1002/pola.1989.080271207.

[ref6] Villotte S.; Gigmes D.; Dumur F.; Lalevée J. Design of Iodonium Salts for UV or Near-UV LEDs for Photoacid Generator and Polymerization Purposes. Molecules 2020, 25, 149 10.3390/molecules25010149. PMC6982746 31905900

[ref7] Crivello J. V.; Lam J. H. W. Photosensitive polymers containing diaryliodonium salt groups in the main chain. J. Polym. Sci. Polym. Chem. Ed. 1979, 17, 3845–3858. 10.1002/pol.1979.170171205.

[ref8] Saeva F. D.; Morgan B. P.; Luss H. R. Photochemical conversion of sulfonium salts to sulfides via a 1,3-sigmatropic rearrangement. Photogeneration of Broensted acids. J. Org. Chem. 1985, 50, 4360–4362. 10.1021/jo00222a031.

[ref9] Crivello J. V.; Lam J. H. W. Photoinitiated cationic polymerization by dialkyl-4-hydroxyphenylsulfonium salts. J. Polym. Sci. Polym. Chem. Ed. 1980, 18, 1021–1034. 10.1002/pol.1980.170180321.

[ref10] Crivello J. V.; Lee J. L. Photosensitized cationic polymerizations using dialkylphenacylsulfonium and dialkyl(4-hydroxyphenyl)sulfonium salt photoinitiators. Macromolecules 1981, 14, 1141–1147. 10.1021/ma50006a001.

[ref11] Neckers D. C.; Abu-Abdoun I. I. p,p’-Bis((triphenylphosphonio)methyl)benzophenone salts as photoinitiators of free radical and cationic polymerization. Macromolecules 1984, 17, 2468–2473. 10.1021/ma00142a003.

[ref12] Abu-Abdoun I. I.; Ali A. Cationic photopolymerization of p-methylstyrene initiated by phosphonium and arsonium salts. Eur. Polym. J. 1993, 29, 1439–1443. 10.1016/0014-3057(93)90055-K.

[ref13] Komoto K.; Ishidoya M.; Ogawa H.; Sawada H.; Okuma K.; Ohata H. Photopolymerization of vinyl ether by hydroxy- and methylthio-alkylphosphonium salts as novel photocationic initiators. Polymer 1994, 35, 217–218. 10.1016/0032-3861(94)90077-9.

[ref14] Scaiano J. C.; Barra M.; Krzywinski M.; Sinta R.; Calabrese G. Laser flash photolysis determination of absolute rate constants for reactions of bromine atoms in solution. J. Am. Chem. Soc. 1993, 115, 8340–8344. 10.1021/ja00071a048.

[ref15] Gannon T.; McGimpsey W. G. Photochemistry of trans-10,11-dibromodibenzosuberone: a near-UV photoacid generator. J. Org. Chem. 1993, 58, 913–916. 10.1021/jo00056a025.

[ref16] Fouassier J. P.; Burr D. Triplet state reactivity of α-sulfonyloxy ketones used as polymerization photoinitiators. Macromolecules 1990, 23, 3615–3619. 10.1021/ma00217a013.

[ref17] Ruhlmann D.; Fouassier J. P. Relations structure-proprietes dans les photoamorceurs de polymerisation—8. les derives de sulfonyl cetones. Eur. Polym. J. 1993, 29, 1079–1088. 10.1016/0014-3057(93)90313-5.

[ref18] Asakura T.; Yamato H.; Ohwa M. Novel Photoacid Generators. J. Photopolym. Sci. Technol. 2000, 13, 223–230. 10.2494/photopolymer.13.223.

[ref19] Ortica F.; Scaiano J. C.; Pohlers G.; Cameron J. F.; Zampini A. Laser Flash Photolysis Study of Two Aromatic *N*-Oxyimidosulfonate Photoacid Generators. Chem. Mater. 2000, 12, 414–420. 10.1021/cm990440t.

[ref20] Lopez de Pariza X.; Cordero Jara E.; Zivic N.; Ruiperez F.; Long T. E.; Sardon H. Novel imino- and aryl-sulfonate based photoacid generators for the cationic ring-opening polymerization of ε-caprolactone. Polym. Chem. 2021, 12, 4035–4042. 10.1039/D1PY00734C.

[ref21] Ravelli D.; Fagnoni M. In CRC Handbook of Organic Photochemistry and Photobiology, 3rd ed.; Griesbeck A., Oelgemoeller M., Ghetti F., Eds.; CRC Press, 2012; pp 393–417.

[ref22] Langler R. F.; Marini Z. A.; Pincock J. A. The photochemistry of benzylic sulfonyl compounds: The preparation of sulfones and sulfinic acids. Can. J. Chem. 1978, 56, 903–907. 10.1139/v78-151.

[ref23] Torti E.; Della Giustina G.; Protti S.; Merli D.; Brusatin G.; Fagnoni M. Aryl tosylates as non-ionic photoacid generators (PAGs): photochemistry and applications in cationic photopolymerizations. RSC Adv. 2015, 5, 33239–33248. 10.1039/C5RA03522H.

[ref24] Torti E.; Protti S.; Merli D.; Dondi D.; Fagnoni M. Photochemistry of N-Arylsulfonimides. An easy available class of non ionic PhotoAcid Generators (PAGs). Chem.—Eur. J. 2016, 22, 16998–17005. 10.1002/chem.201603522. 27739113

[ref25] aIwashima C.; Imai G.; Okamura H.; Tsunooka M.; Shirai M. Synthesis of i- and g-Line Sensitive Photoacid Generators and Their Application to Photopolymer Systems. J. Photopolym. Sci. Technol. 2003, 16, 91–96. 10.2494/photopolymer.16.91.

[ref26] aTakahashi Y.; Kodama S.; Ishii Y. Visible-Light-Sensitive Sulfonium Photoacid Generators Bearing a Ferrocenyl Chromophore. Organometallics 2018, 37, 1649–1651. 10.1021/acs.organomet.8b00203.

[ref27] Sambath K.; Wan Z.; Wang Q.; Chen H.; Zhang Y. BODIPY-Based Photoacid Generators for Light-Induced Cationic Polymerization. Org. Lett. 2020, 22, 1208–1212. 10.1021/acs.orglett.0c00118. 31944778

[ref28] aSilvi S.; Arduini A.; Pochini A.; Secchi A.; Tomasulo M.; Raymo F. M.; Baroncini M.; Credi A. J A Simple Molecular Machine Operated by Photoinduced Proton Transfer. J. Am. Chem. Soc. 2007, 129, 13378–13379. 10.1021/ja0753851. 17935334

[ref29] Qiu D.; Lian C.; Mao J.; Fagnoni M.; Protti S. Dyedauxiliary groups, an emerging approach in organic chemistry. The case of arylazo sulfones. J. Org. Chem. 2020, 85, 12813–12822. 10.1021/acs.joc.0c01895. 32956584 PMC8011925

[ref30] Blank L.; Fagnoni M.; Protti S.; Rueping M. Visible-Light Promoted Formation of C-B and C-S Bonds under metal and photocatalyst-free conditions. Synthesis 2019, 51, 1243–1252. 10.1055/s-0037-1611648.

[ref31] aLiu Q.; Wang L.; Yue H.; Li J.; Luo Z.; Wei W. Catalyst-free visible-light-initiated oxidative coupling of aryldiazo sulfones with thiols leading to unsymmetrical sulfoxides in air. Green Chem. 2019, 21, 1609–1613. 10.1039/C9GC00222G.

[ref32] Othman Abdulla H.; Scaringi S.; Amin A. A.; Mella M.; Protti S.; Fagnoni M. Aryldiazenyl Radicals from Arylazo Sulfones: Visible Light-Driven Diazenylation of Enol Silyl Ethers. Adv. Synth. Catal. 2020, 362, 2150–2154. 10.1002/adsc.201901424.

[ref33] Kobayashi M.; Fujii S.; Minato H. Photolysis of Phenylazo p-Tolyl Sulfones. Bull. Chem. Soc. Jpn. 1972, 45, 2039–2042. 10.1246/bcsj.45.2039.

[ref34] aChawla R.; Jaiswal S.; Dutta P. K.; Yadav L. D. S. A photocatalyst-free visible-light-mediated solvent-switchable route to stilbenes/vinyl sulfones from β-nitrostyrenes and arylazo sulfones. Org. Biomol. Chem. 2021, 19, 6487–6492. 10.1039/D1OB01028J. 34241618

[ref35] Bolton J. L.; Dunlap T. Formation and Biological Targets of Quinones: Cytotoxic versus Cytoprotective Effects. Chem. Res. Toxicol. 2017, 30 (1), 13–37. 10.1021/acs.chemrestox.6b00256. 27617882 PMC5241708

[ref36] Crespi S.; Protti S.; Fagnoni M. Wavelength Selective Generation of Aryl Radicals and Aryl Cations for Metal-Free Photoarylations. J. Org. Chem. 2016, 81, 9612–9619. 10.1021/acs.joc.6b01619. 27696841

[ref37] Nitti A.; Martinelli A.; Batteux F.; Protti S.; Fagnoni M.; Pasini D. Blue Light Driven Free-Radical Polymerization using Arylazo Sulfones as Initiators. Polym. Chem. 2021, 12, 5747–5751. 10.1039/D1PY00928A.

[ref38] Médard J.; Decorse P.; Mangeney C.; Pinson J. M.; Fagnoni S. Protti, Simultaneous Photografting of Two Organic Groups on a Gold Surface by using Arylazo Sulfones As the Single Precursors. Langmuir 2020, 36, 2786–2793. 10.1021/acs.langmuir.9b03878. 32090577

[ref39] Lombardi L.; Kovtun A.; Mantovani S.; Bertuzzi G.; Favaretto L.; Bettini C.; Palermo V.; Melucci M.; Bandini M. Visible-Light Assisted Covalent Surface Functionalization of Reduced Graphene Oxide Nanosheets with Arylazo Sulfones. Chem.—Eur. J. 2022, 28, e202200333 10.1002/chem.202200333. 35319124

[ref40] Bertrand M. Recent progress in the use of sulfonyl radicals in organic synthesis. A review. Org. Prep. Proced. Int. 1994, 26, 257–290. 10.1080/00304949409458426.

[ref41] aHorowitz A. Radiolytic decomposition of methanesulfonyl chloride in liquid cyclohexane. A kinetic determination of the bond dissociation energies D(ME-SO_2_) and D(c-C_6_H_11_-SO_2_). Int. J. Chem. Kinet. 1976, 8, 709–723. 10.1002/kin.550080507.

[ref42] Wuts P. G. M. Greene’s Protective Groups in Organic Synthesis, 5th ed.; John Wiley & Sons: Hoboken, 2014.

[ref43] Sartori G.; Ballini R.; Bigi F.; Bosica G.; Maggi R.; Righi P. Protection (and Deprotection) of Functional Groups in Organic Synthesis by Heterogeneous Catalysis. Chem. Rev. 2004, 104, 199–250. 10.1021/cr0200769. 14719975

[ref44] aRoss P. S.; Rahman A. A.; Sigman M. S. Development and Mechanistic Interrogation of Interrupted Chain-Walking in the Enantioselective Relay Heck Reaction. J. Am. Chem. Soc. 2020, 142, 10516–10525. 10.1021/jacs.0c03589. 32412759 PMC7376753

[ref45] Oates R. P.; Jones P. B. Photosensitized Tetrahydropyran Transfer. J. Org. Chem. 2008, 73, 4743–4745. 10.1021/jo800519h. 18505291

[ref46] Lian C.; Yue G.; Mao J.; Liu D.; Ding Y.; Liu Z.; Qiu D.; Zhao X.; Lu K.; Fagnoni M.; Protti S. Visible-light-driven synthesis of arylstannanes from arylazo sulfones. Org. Lett. 2019, 21, 5187–5191. 10.1021/acs.orglett.9b01788. 31247810

[ref47] Evrard D.; Lambert F.; Policar C.; Balland V.; Limoges B. Electrochemical functionalization of carbon surfaces by aromatic azide or alkyne molecules: a versatile platform for click chemistry. Chem.—Eur. J. 2008, 14, 9286–9291. 10.1002/chem.200801168. 18780382

[ref48] Crouch R. D.; Richardson A.; Howard J. L.; Harker R. L.; Barker K. H. The Aldol Addition and Condensation: The Effect of Conditions on Reaction Pathway. J. Chem. Educ. 2007, 84, 475–476. 10.1021/ed084p475.

[ref49] Cornil J.; Echeverria P.-G.; Reymond S.; Phansavath P.; Ratovelomanana-Vidal V.; Guérinot A.; Cossy J. Synthetic Studies toward the C14–C29 Fragment of Mirabalin. Org. Lett. 2016, 18, 4534–4537. 10.1021/acs.orglett.6b02162. 27602486

[ref50] Duan Z.; Gu Y.; Deng Y. Brønsted Acidic Ionic Liquids: Fast, Mild, and Efficient Catalysts for Solvent-Free Tetrahydropyranylation of Alcohols. Synth. Commun. 2005, 35, 1939–1945. 10.1081/SCC-200064995.

[ref51] Jawor M. L.; Ahmed B. M.; Mezei G. Solvent-and catalyst-free, quantitative protection of hydroxyl, thiol, carboxylic acid, amide and heterocyclic amino functional groups. Green Chem. 2016, 18, 6209–6214. 10.1039/C6GC02562E.

[ref52] Hajipour A. R.; Pourmousavi S. A.; Ruoho A. E. Simple and facile tetrahydropyranylation of alcohols by use of catalytic amounts of benzyltriphenylphosphonium tribromide. Synth. Commun. 2005, 35, 2889–2894. 10.1080/00397910500297297.

[ref53] Hon Y.-S.; Lee C.-F.; Chen R.-J.; Szu P.-H. Acetonyltriphenylphosphonium bromide and its polymer-supported analogues as catalysts in protection and deprotection of alcohols as alkyl vinyl ethers. Tetrahedron 2001, 57, 5991–6001. 10.1016/S0040-4020(01)00558-0.

[ref54] Markó I. E; Ates A.; Augustyns B.; Gautier A.; Quesnel Y.; Turet L.; Wiaux M. Remarkable deprotection of THP and THF ethers catalysed by cerium ammonium nitrate (CAN) under neutral conditions. Tetrahedron Lett. 1999, 40, 5613–5616. 10.1016/S0040-4039(99)01043-6.

[ref55] Kotke M.; Schreiner P. Generally applicable organocatalytic tetrahydropyranylation of hydroxy functionalities with very low catalyst loading. Synthesis 2007, 779–790. 10.1055/s-2007-965917.

[ref56] Chandrasekhar S.; Takhi M.; Reddy Y. R.; Mohapatra S.; Rao C. R.; Reddy K. V. TaCl5-silicagel and TaCl_5_ as new Lewis acid systems for selective tetrahydropyranylation of alcohols and thioacetalisation, trimerisation and aldolisation of aldehydes. Tetrahedron 1997, 53, 14997–15004. 10.1016/S0040-4020(97)01051-X.

[ref57] Semwal A.; Nayak S. K. Ti (III) chloride: A novel reagent for the chemoselective deprotection of tetrahydropyranyl ethers. Synthesis 2005, 1, 71–74. 10.1055/s-2004-834949.

[ref58] Wiles C.; Watts P. Parallel synthesis in an EOF-based micro reactor. Chem. Commun. 2007, 46, 4928–4930. 10.1039/b712546a. 18361372

[ref59] Da Silveira Neto B. A.; Ebeling G.; Gonçalves R. S.; Gozzo F. C.; Eberlin M. N.; Dupont J. Organoindate room temperature ionic liquid: Synthesis, physicochemical properties and application. Synthesis 2004, 1155–1158. 10.1055/s-2004-822372.

[ref60] Gopinath P.; Nilaya S.; Muraleedharan K. Highly chemoselective esterification reactions and Boc/THP/TBDMS discriminating deprotections under Samarium (III) Catalysis. Org. Lett. 2011, 13, 1932–1935. 10.1021/ol200247c. 21395228

[ref61] Hon Y.-S.; Lee C.-F.; Chen R.-J.; Szu P.-H. Acetonyltriphenylphosphonium bromide and its polymer-supported analogues as catalysts in protection and deprotection of alcohols as alkyl vinyl ethers. Tetrahedron 2001, 57, 5991–6001. 10.1016/S0040-4020(01)00558-0.

