# Supplementary material for: Arylazo Sulfones as Nonionic Visible-Light Photoacid Generators
Source: J Org Chem. 2022 Jul 22;88(10):6313–21. doi: 10.1021/acs.joc.2c01248 (PMC10204067; doi:10.1021/acs.joc.2c01248)
Supplement: Supplementary file 1 — jo2c01248_si_001.pdf [file jo2c01248_si_001.pdf]

Arylazo sulfones as non-ionic visible-light photoacid generators

Lorenzo Di Terlizzi,[a] Angelo Martinelli,[b] Daniele Merli,[b] Stefano Protti[a] and Maurizio Fagnoni[a]*

[a] PhotoGreen Lab, Department of Chemistry, University of Pavia, Viale Taramelli 12, 27100 Pavia, Italy

[b] Department of Chemistry, University of Pavia, Viale Taramelli 12, 27100 Pavia, Italy

1. **Figures and Tables** **S2**
2. **Potentiometric titrations of a 2.5×10^{-2} M solution of 1a-k after irradiation in MeCN** **S5**
3. **GC-MS analysis** **S11**
4. **Experimental section** **S13**
5. **Copy of the ^{1}H and ^{13}C NMR of compounds 6-19** **S17**

1. Figures and Tables

Table S1. Molar extinction coefficient and λ_{max} of arylazo sulfones **1a-k**.[a]

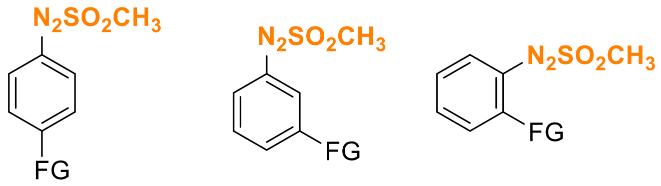

1a, FG = $COCH_3$ **1h**, FG = $COCH_3$ **1j**, FG = CN
1b, FG = Br **1i**, FG = CN **1k**, FG = OCH_3
1c, FG = CN
1d, FG = Cl
1e, FG = CH_3
1f, FG = OCH_3
1g, FG = NO_2

Arylazo sulfones	λ_{max} (nm), ε (mol L^{-1} cm^{-1})
**1a**	294, (15921)<br>432, (181)
**1b**	309, (16355)<br>425, (210)
**1c**	288, (19568)<br>435, (155)
**1d**	305, (14003)<br>424, (167)
**1e**	310, (15264)<br>420, (215)
**1f**	342, (17405)<br>425, (230)
**1g**	287, (15360)<br>395, (3206)
**1h**	289, (12413)<br>425, (157)
**1i**	289, (12680)<br>427, (140)
**1j**	290, (13492)<br>436, (123)
**1k**	294, (9238)<br>395, (3205)

[a] Conditions: A 10^{-4} M solution of **1a-k** was used to evaluate the maximum of absorption in the UV region whereas a 5×10^{-4} M solution of **1a-k** was used to evaluate the maximum of absorption in the visible region.

Table S2. Irradiation of **1d** in argon purged acetonitrile at 456 nm.[a]

Time (min)	**1d**, consumption %[b]	**2d**, Yield %[c]
10	5	4
30	19	13
60	45	30
80	52	35
120	76	49
180	100	63

[a] Conditions: an argon purged solution of **1d** (2.5×10^{-2} M) was irradiated with a 40 W Kessil Lamp with emission centred at 456 nm. [b] The consumption of the reagent was monitored through HPLC analysis. [c] Yield of **2d** was determined through GC-FID analysis.

Table S3. *p*Ka table of phenols **3a-k** possibly obtained by irradiation of **1a-k** in oxygen purged experiments.

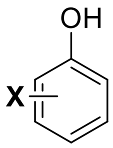

3a-k

3a: X= 4-Ac
3b: X= 4-Br
3c: X= 4-CN
3d: X= 4-Cl
3e: X= 4-CH_3
3f: X= 4-OCH_3
3g: X= 4-NO_2
3h: X= 3-Ac
3i: X= 3-CN
3j: X= 2-CN
3k: X= 2-OCH_3

Compound	*p*Ka
**3a**	8.12 [S1]
**3b**	9.34 [S2]
**3c**	7.97 [S3]
**3d**	9.43 [S4]
**3e**	10.26 [S2]
**3f**	10.20 [S4]
**3g**	7.15 [S2]
**3h**	9.19 [S5]
**3i**	8.61 [S3]
**3j**	6.86 [S3]
**3k**	10.68 [S6]

2. Potentiometric titrations of a 2.5×10^{-2} M solution of 1a-k after irradiation in MeCN.

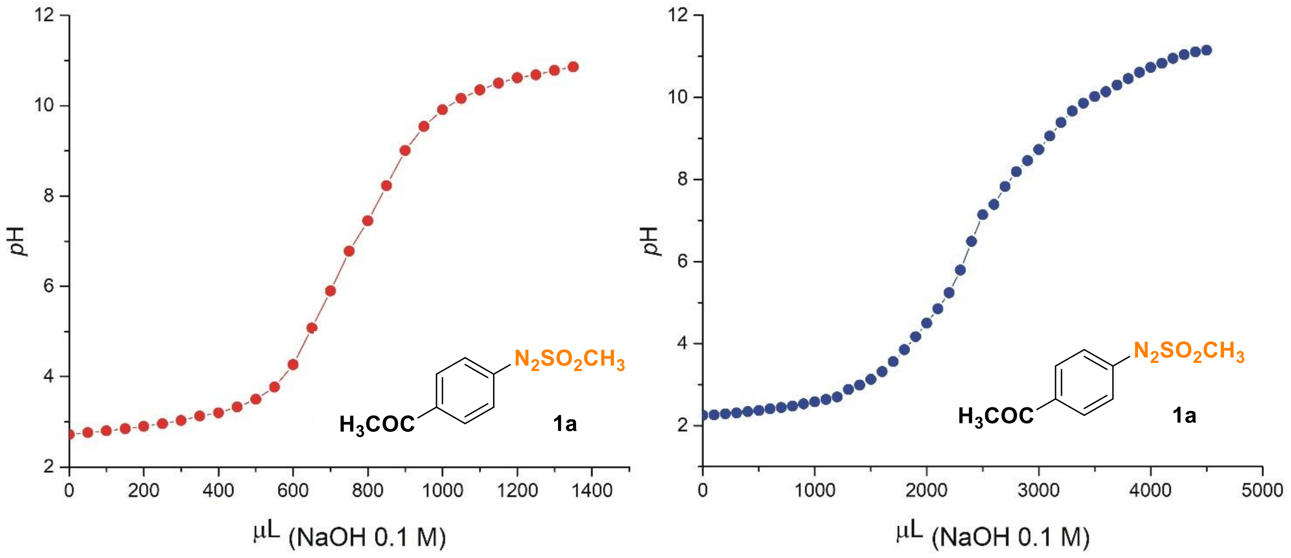

Figure S1. Potentiometric titration of 10 mL a 2.5×10^{-2} M solution of **1a** irradiated in MeCN for 3 h (in red the titration of the Argon purged solution while in blue that of the oxygenated one).

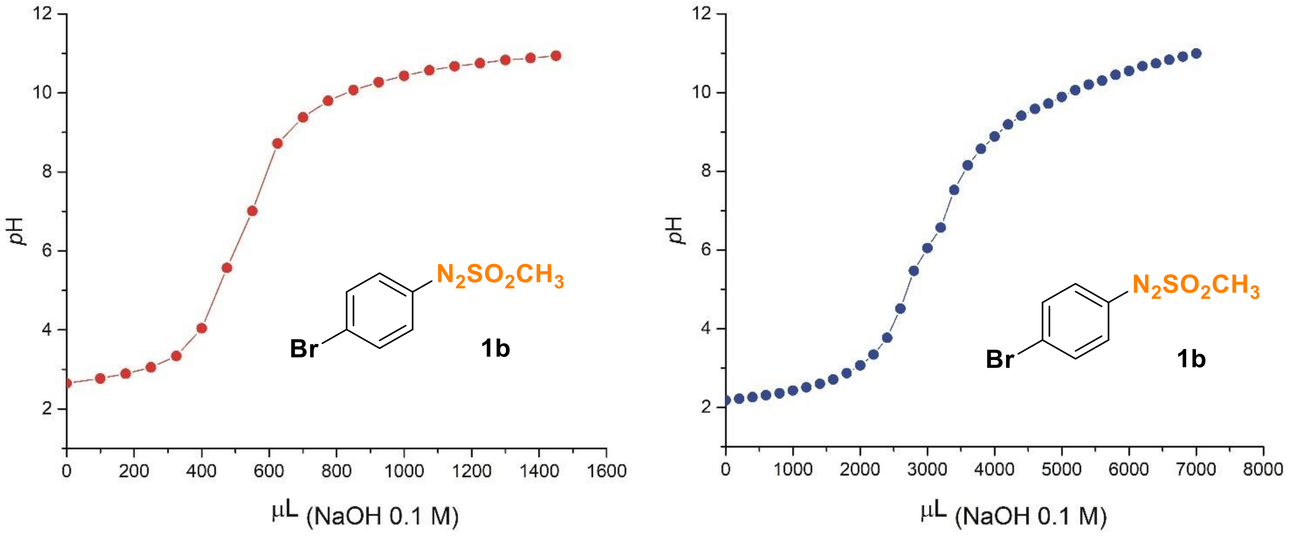

Figure S2. Potentiometric titration of 10 mL of a 2.5×10^{-2} M solution of **1b** irradiated in MeCN for 3 h (in red the titration of the Argon purged solution while in blue that of the oxygenated one).

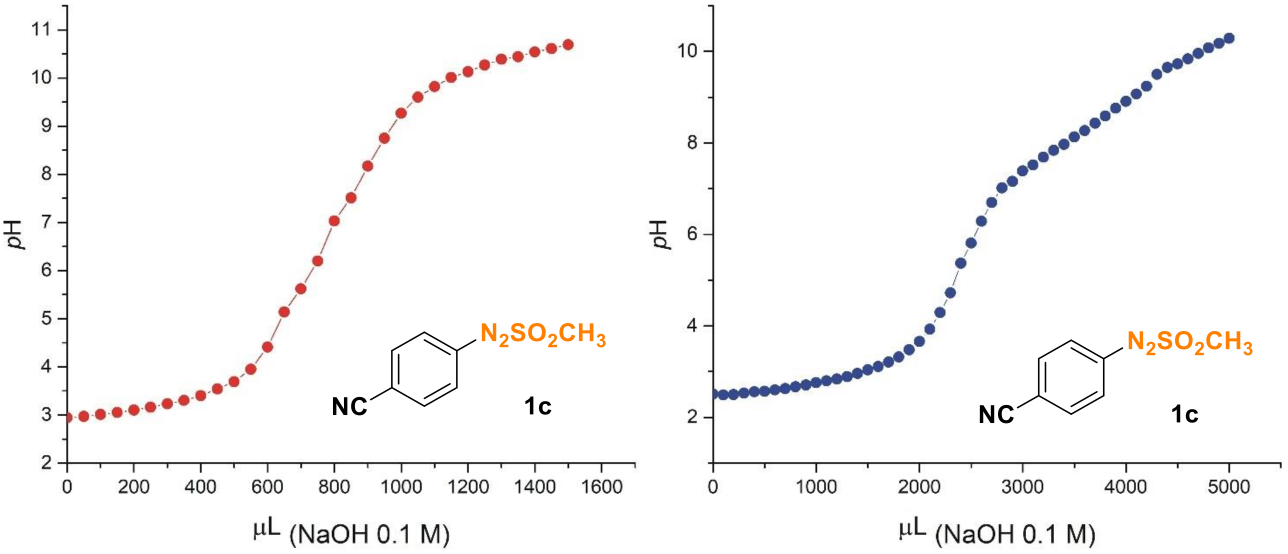

Figure S3. Potentiometric titration of 10 mL of a 2.5×10^{-2} M solution of **1c** irradiated in MeCN for 3 h (in red the titration of the Argon purged solution while in blue that of the oxygenated one).

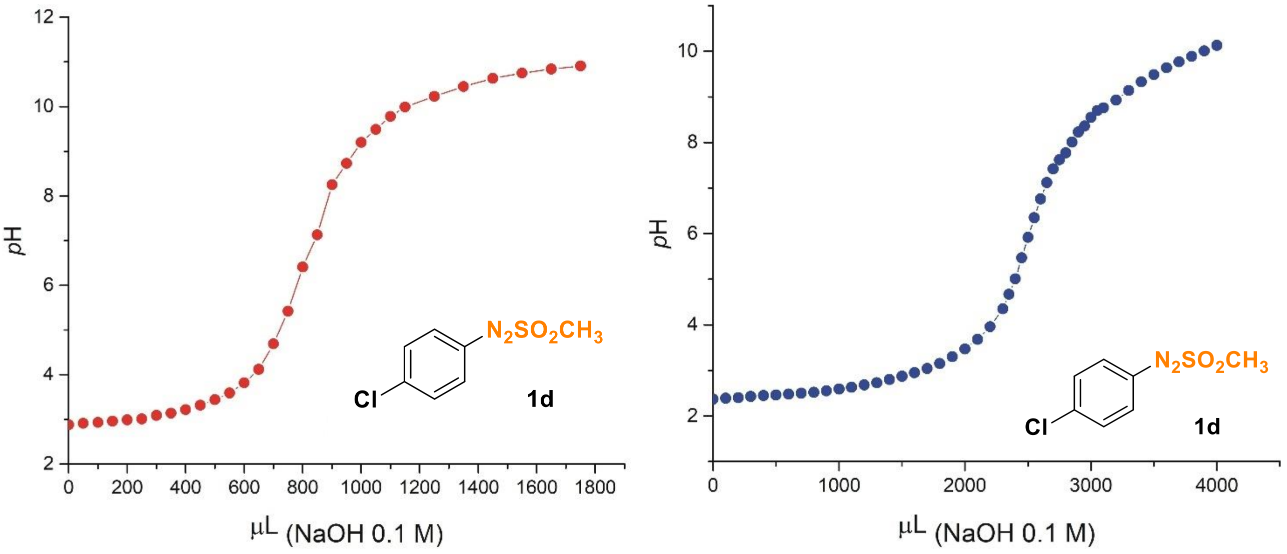

Figure S4. Potentiometric titration of 10 mL of a 2.5×10^{-2} M solution of **1d** irradiated in MeCN for 3 h (in red the titration of the Argon purged solution while in blue that of the oxygenated one).

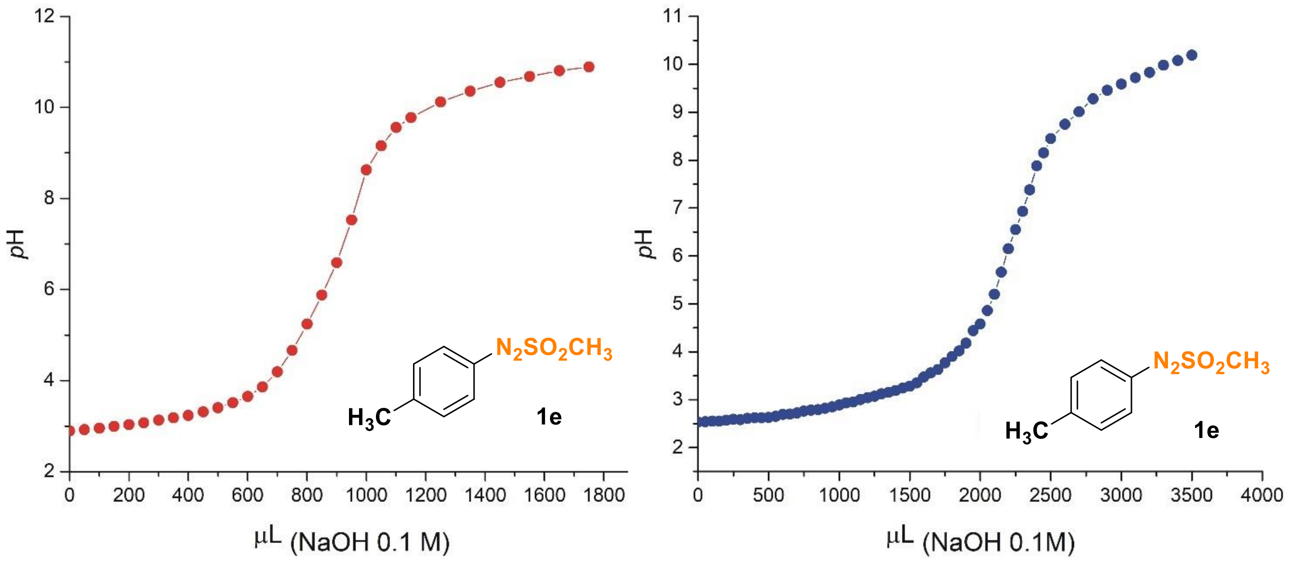

Figure S5. Potentiometric titration of 10 mL of a 2.5×10^{-2} M solution of **1e** irradiated in MeCN for 3 h (in red the titration of the Argon purged solution while in blue that of the oxygenated one).

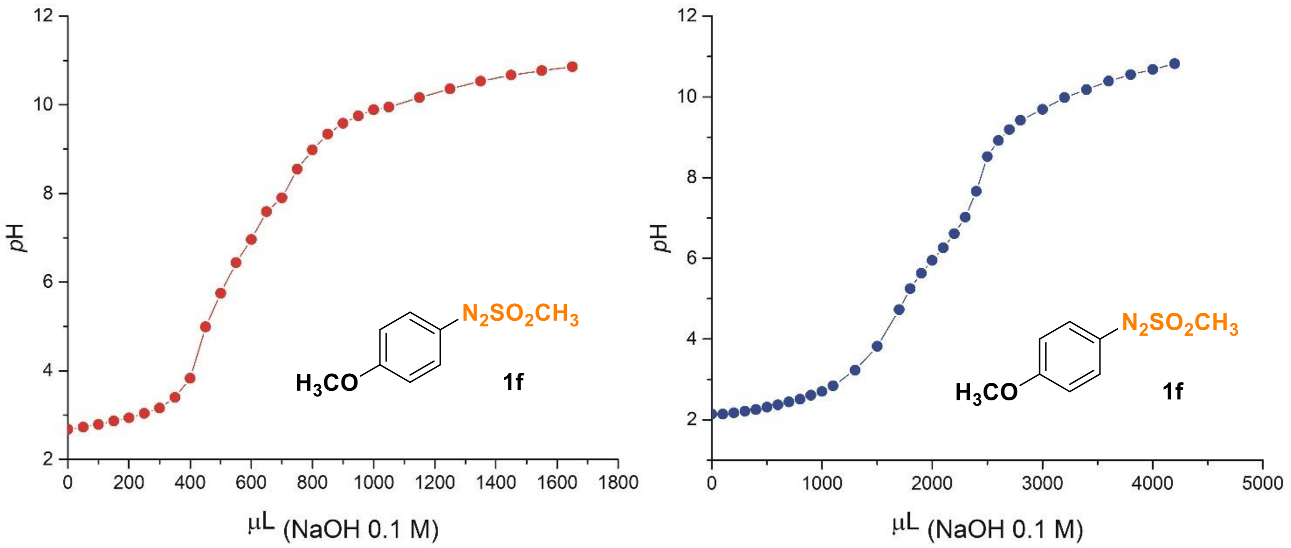

Figure S6. Potentiometric titration of 10 mL of a 2.5×10^{-2} M solution of **1f** irradiated in MeCN for 3 h (in red the titration of the Argon purged solution while in blue that of the oxygenated one).

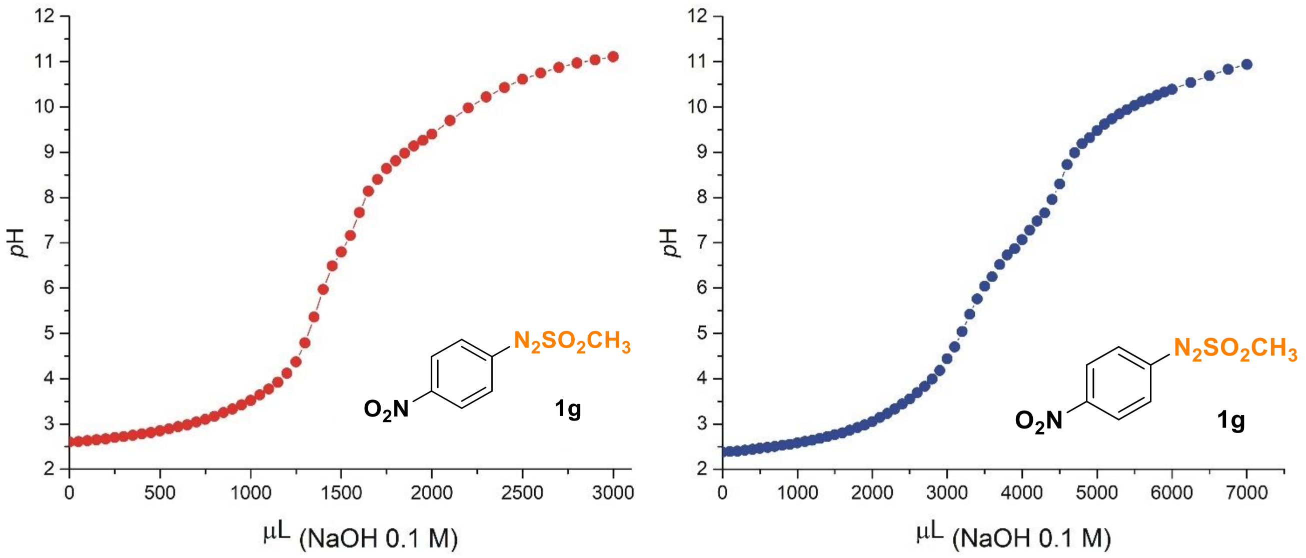

Figure S7. Potentiometric titration of 10 mL of a 2.5×10^{-2} M solution of **1g** irradiated in MeCN for 3 h (in red the titration of the Argon purged solution while in blue that of the oxygenated one).

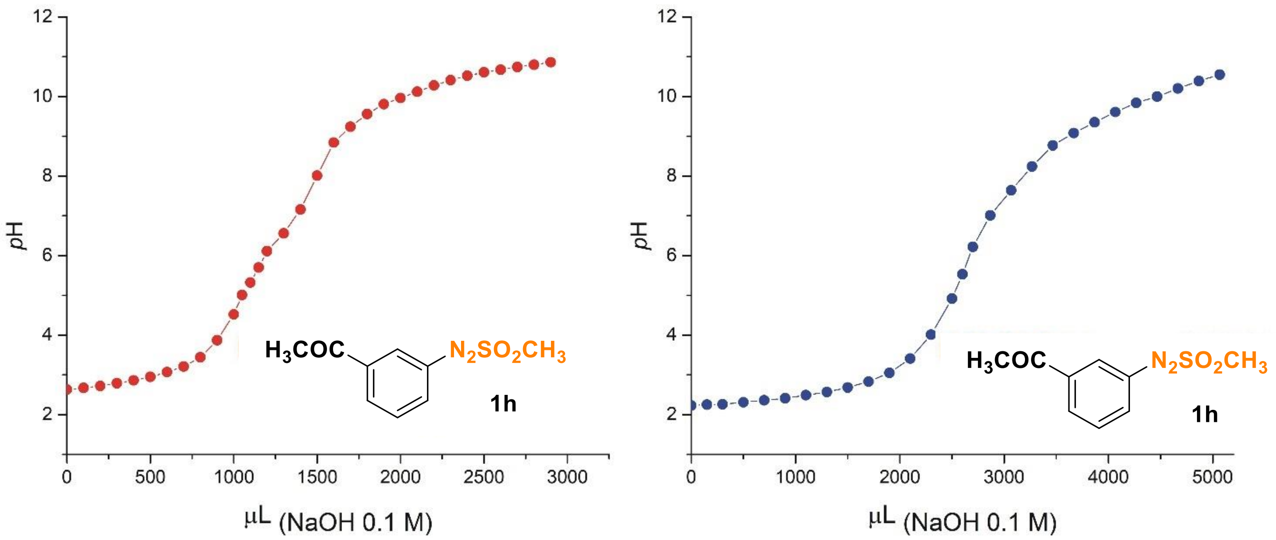

Figure S8. Potentiometric titration of 10 mL of a 2.5×10^{-2} M solution of **1h** irradiated in MeCN for 3 h (in red the titration of the Argon purged solution while in blue that of the oxygenated one).

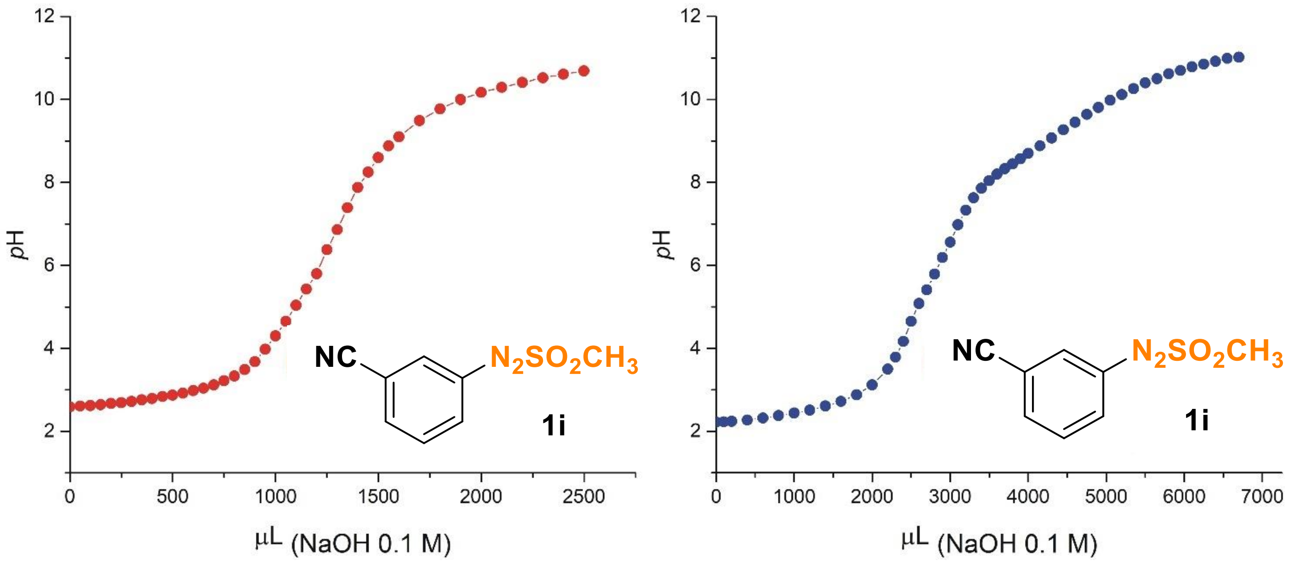

Figure S9. Potentiometric titration of 10 mL of a 2.5×10^{-2} M solution of **1i** irradiated in MeCN for 3 h (in red the titration of the Argon purged solution while in blue that of the oxygenated one).

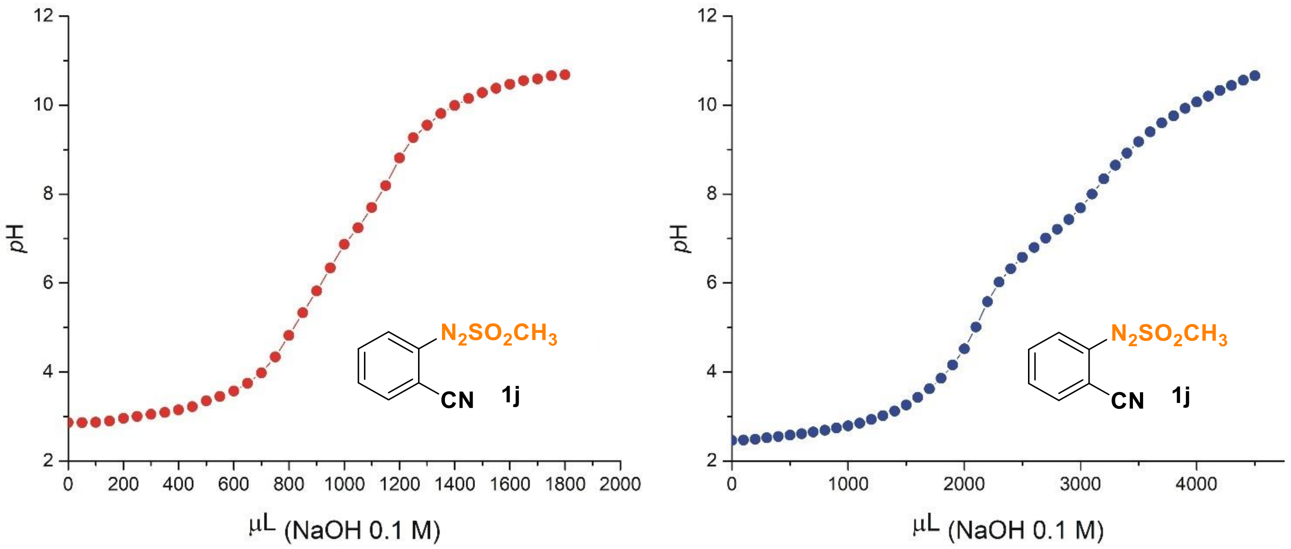

Figure S10. Potentiometric titration of 10 mL of a 2.5×10^{-2} M solution of **1j** irradiated in MeCN for 3 h (in red the titration of the Argon purged solution while in blue that of the oxygenated one).

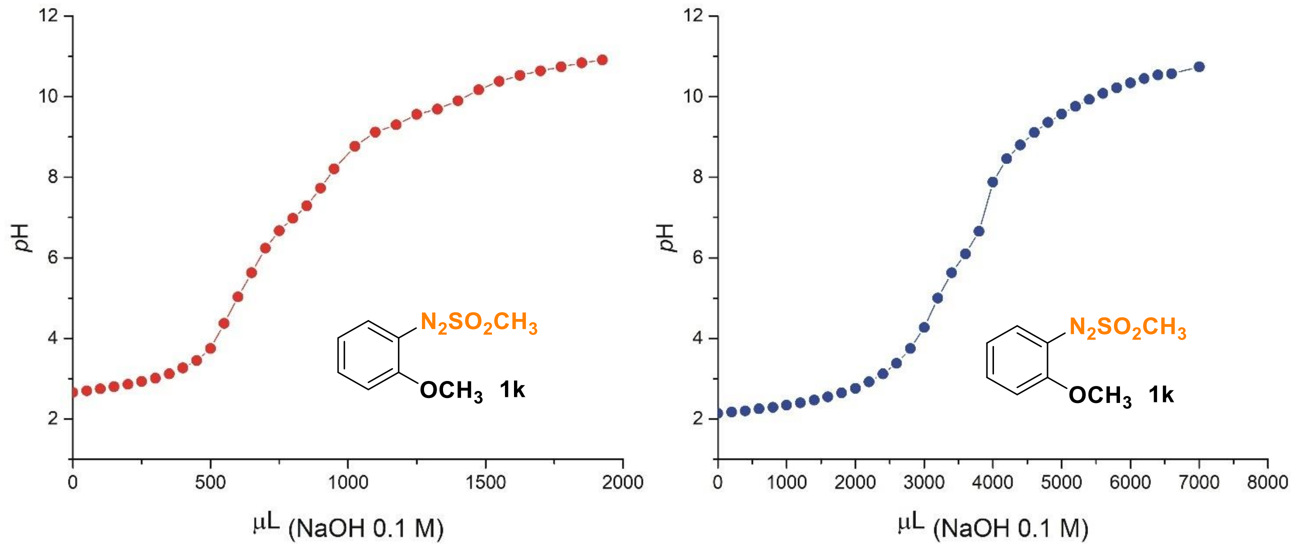

Figure S11. Potentiometric titration of 10 mL of a 2.5×10^{-2} M solution of **1k** irradiated in MeCN for 3 h (in red the titration of the Argon purged solution while in blue that of the oxygenated one).

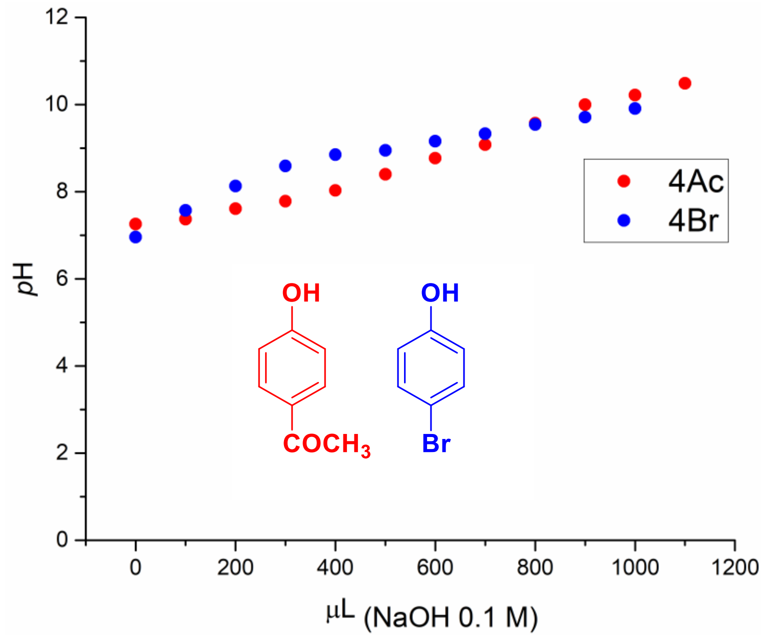

Figure S12. Potentiometric titration of 10 mL of a 2.5×10^{-2} M solution of **3a** (in red) and **3b** (in blue).

The results of the present experiments exclude the contribution of phenols to the total acidity released by the irradiated arylazo sulfones.

3. GC-MS Analysis.

GC-MS analysis were carried out to verify the presence of sulfur containing products different from methanesulfonic or methanesulfinic acid. An oxygen purged solution of **1a** and an argon purged solution of **1f** were irradiated for 3 h in a sealed vial. After the irradiation the resulting mixtures were heated to 40 °C and the head space was injected with a sealed syringe. GC−MS analyses were carried out using a Thermo Scientific DSQII single quadrupole GC−MS system. A Restek Rtx-5MS (30 m × 0.25 mm × 0.25 μm) capillary column was used for the separation of analytes with helium as a carrier gas at 1 mL/min. The injection in the GC system was performed in splitless mode, and the injector temperature was 250 °C. The GC oven temperature was held at 35 °C for 5 min, increased to 150 °C by a temperature ramp of 3 °C min^{-1}, and held for 10 min. The transfer line temperature was 250 °C, and the ion source temperature was 250 °C. Mass spectral analyses were carried out in full scan mode. In both cases the main product was sulfur dioxide (M^+ = 64).

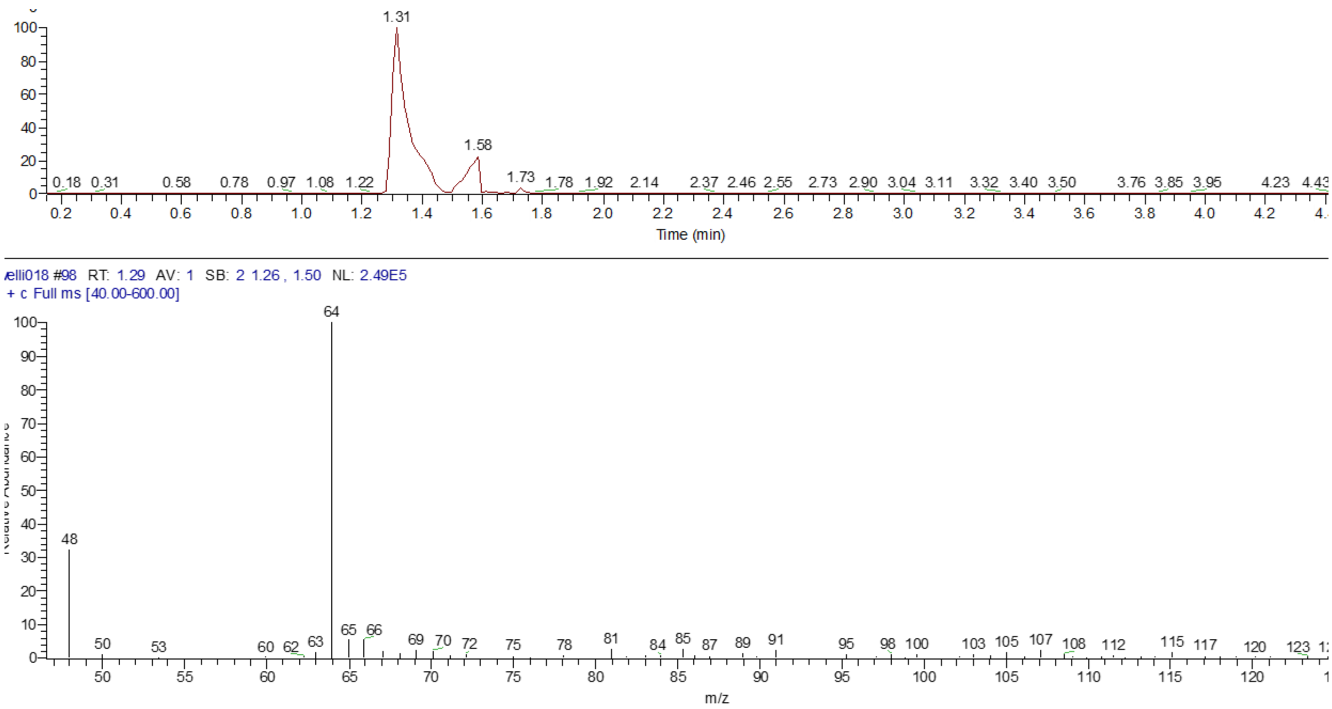

Figure S13. GC-MS analysis of the head space of a **1a** solution irradiated in oxygen-purged media

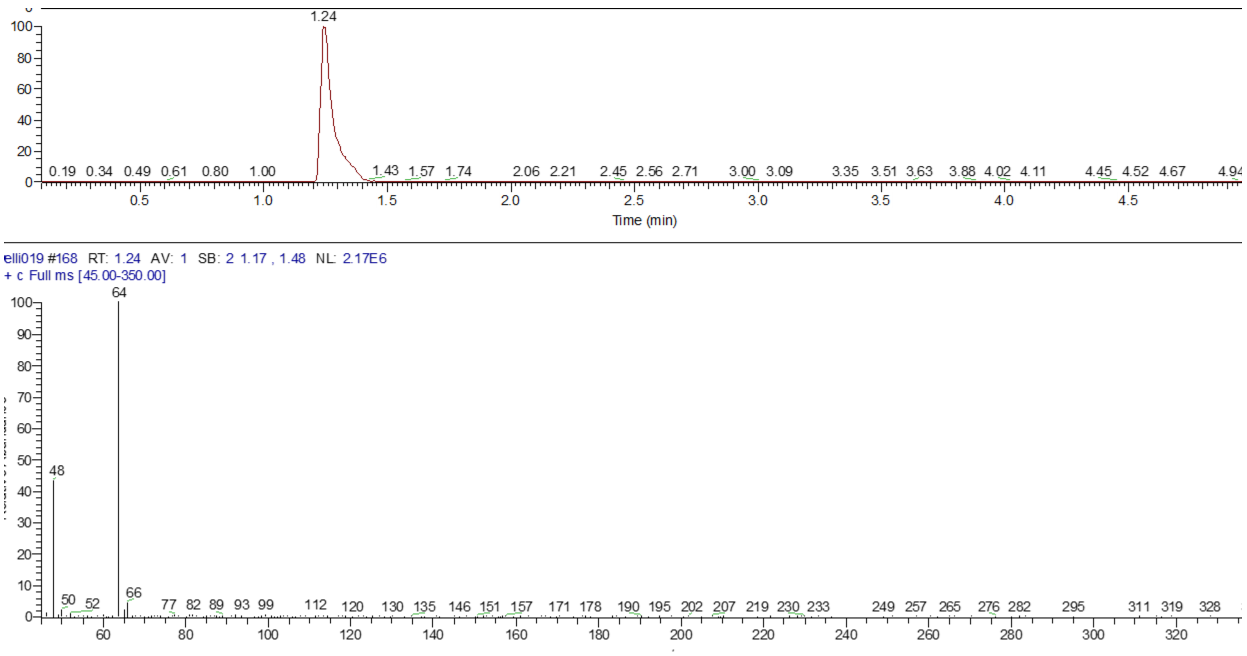

Figure S14. GC-MS analysis of the head space of a **1f** solution irradiated in argon-purged media.

4. Experimental section.

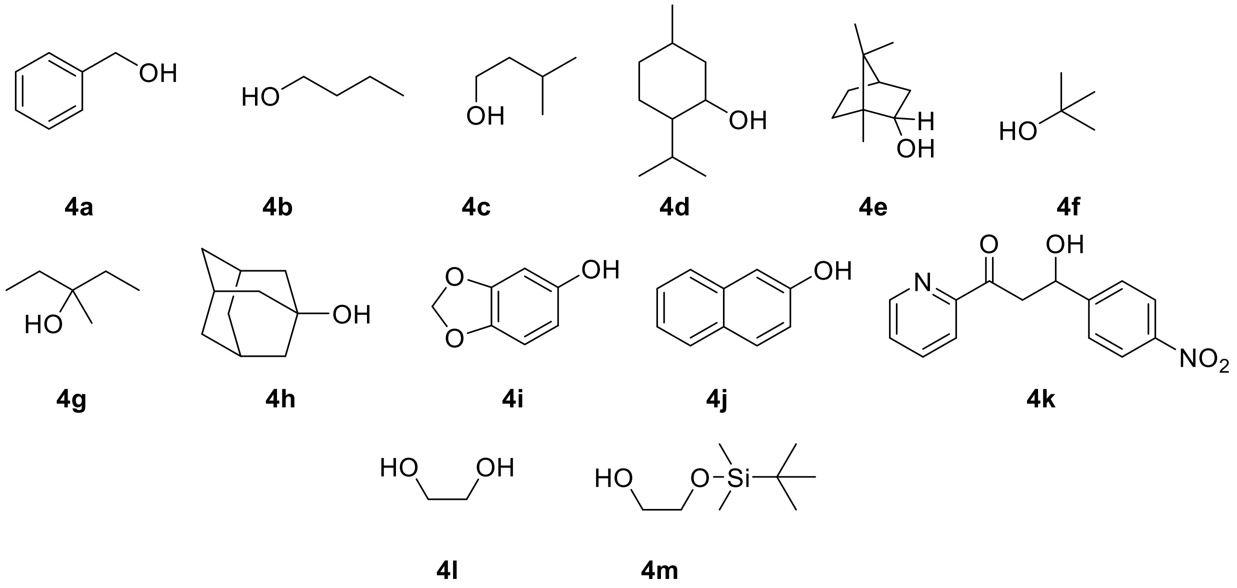

Figure S15. Alcohol employed for the synthesis of tetrahydropyranyl ethers or acetals using arylazo sulfones as PAG catalyst.

Table S4. Optimization of the Photocatalyzed Protection of 4a

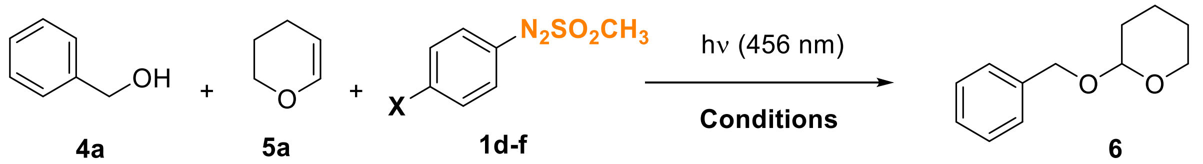

Entry	Conditions	Yield
1	**4a** (0.5 mmol), **5a** (1 equiv), **1e** (10 mol %), DCM, 24 h, O_2	7%
2	**4a** (0.5 mmol), **5a** (1 equiv), **1e** (5 mol %), DCM, 24 h, O_2	80%
3	**4a** (0.5 mmol), **5a** (1 equiv), **1e** (2.5 mol %), DCM, 24 h, O_2	82 %
4	**4a** (0.5 mmol), **5a** (1 equiv), **1e** (1.25 mol %), DCM, 24 h, O_2	85%
5	**4a** (5 mmol), **5a** (1 equiv), **1e** (0.5 mol %), DCM, 24 h, O_2	90%
6	**4a** (5 mmol), **5a** (1.1 equiv), **1e** (0.5 mol %), DCM, 24 h, O_2	>99%
7	**4a** (5 mmol), **5a** (1.1 equiv), **1e** (0.5 mol %), MeCN, 24 h, O_2	>99%
8	**4a** (5 mmol), **5a** (1.1 equiv), **1e** (0.5 mol %), MeCN, 24 h, air equil.	>99%
9	**4a** (5 mmol), **5a** (1.1 equiv), **1e** (0.5 mol %), MeCN, 30 min, air equil.	>99%
10	**4a** (5 mmol), **5a** (1.1 equiv), **1e** (0.5 mol %), MeCN, 30 min, Ar sat..	73%
11	**4a** (5 mmol), **5a** (1.1 equiv), **1d** (0.5 mol %), MeCN, 30 min, air equil.	98%
12	**4a** (5 mmol), **5a** (1.1 equiv), **1f** (0.5 mol %), MeCN, 30 min, air equil.	94%
13	**4a** (5 mmol), **5a** (1.1 equiv), MeCN, 30 min, air equil.	0%
14	**4a** (5 mmol), **5a** (1.1 equiv), **1e** (0.5 mol %), MeCN, 24 h, air equil., Dark	0%
15	**4a** (5 mmol), **5a** (1.1 equiv), **1e** (0.5 mol %), MeCN, 24 h, air equil., 2,6-luditine (0.5 mol %)	0%
16	**4a** (5 mmol), **5a** (1.1 equiv), **PTSA** (0.5 mol %), MeCN, 30 min	0%
17	**4a** (5 mmol), **5a** (1.1 equiv), **MSA** (0.5 mol %), MeCN, 30 min	<5%

PTSA = *p*-Toluenesulfonic acid; **MSA** = Methanesulfonic acid

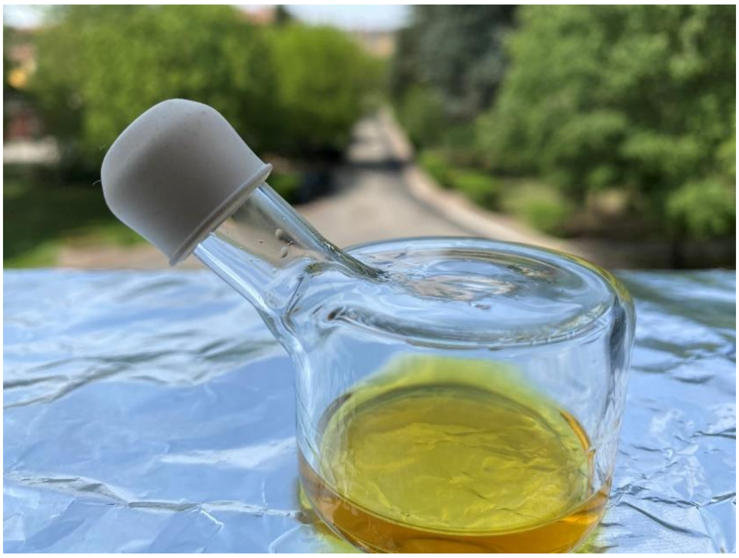

Figure S16. Sunlight promoted synthesis of **6** performed with a Pyrex glass vessel placed outside the window with an aluminium foil underneath to perform the photoinduced protection of benzyl alcohol **4a** (5 mmol) with **5a** (5.5 mmol) in the presence of **1e** (0.5 mol%) in 4 mL of acetonitrile.

Mechanistic studies on alcohol protection.

Three Pyrex glass vessels were charged with arylazo sulfone **1e** (0.5 mol%), benzyl alcohol **4a** (5 mmol, 1 equiv, 1.25 M) and **5a** (5.5 mmol, 1.1 equiv, 1.375 M) in 4 mL of acetonitrile. The so-formed mixtures were irradiated for 5, 15 and 30 minutes respectively using EvoluChem apparatus equipped with a 40W Kessil lamp (emission centred at 456 nm) placed 3 centimetres above the reaction vessel. As the irradiation stopped, Na_2CO_3 was added (5.0 mg, 0.05 mmol) and the product formation was monitored through GC analysis (Table S5, entries 1-3).

Finally, two more glass vessels were charged with arylazo sulfone **1e** (0.025 mmol, 0.5 mol%) with benzyl alcohol **4a** (5 mmol, 1 equiv, 1.25 M) and **5a** (5.5 mmol, 1.1 equiv, 1.375 M) in 4 mL of acetonitrile. The so-formed mixtures were irradiated for 5 min each, then covered with an aluminium foil for 10 min and 25 min respectively. The product formation was monitored through GC analysis (entries 4-5).

Table S5. Mechanistic investigations

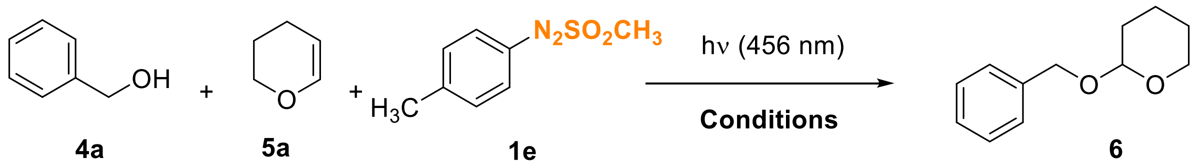

4a 5a 1e 6

Entry	Conditions	Yield
1	**4a** (5 mmol), **5a** (1.1 equiv), **1e** (0.5 mol %), MeCN, 5 min, then Na_2CO_3 (0.05 mmol)	13%
2	**4a** (5 mmol), **5a** (1.1 equiv), **1e** (0.5 mol %), MeCN, 15 min, then Na_2CO_3 (0.05 mmol)	48 %
3	**4a** (5 mmol), **5a** (1.1 equiv), **1e** (0.5 mol %), MeCN, 30 min, then Na_2CO_3 (0.05 mmol)	100%
4	**4a** (5 mmol, 1 equiv), **5a** (5.5 mmol, 1 equiv), **1e** (0.5 mol %), MeCN, 5 minutes, then 10 min in dark	40%
5	**4a** (5 mmol, 1 equiv), **5a** (5.5 mmol, 1 equiv), **1e** (0.5 mol %), MeCN, 5 minutes, then 25 min in dark	84%

5 Copy of ^{1}H and ^{13}C NMR of compounds 6-19.

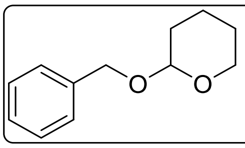

6 ^{1}H-NMR (300 MHz, acetone-d_6)

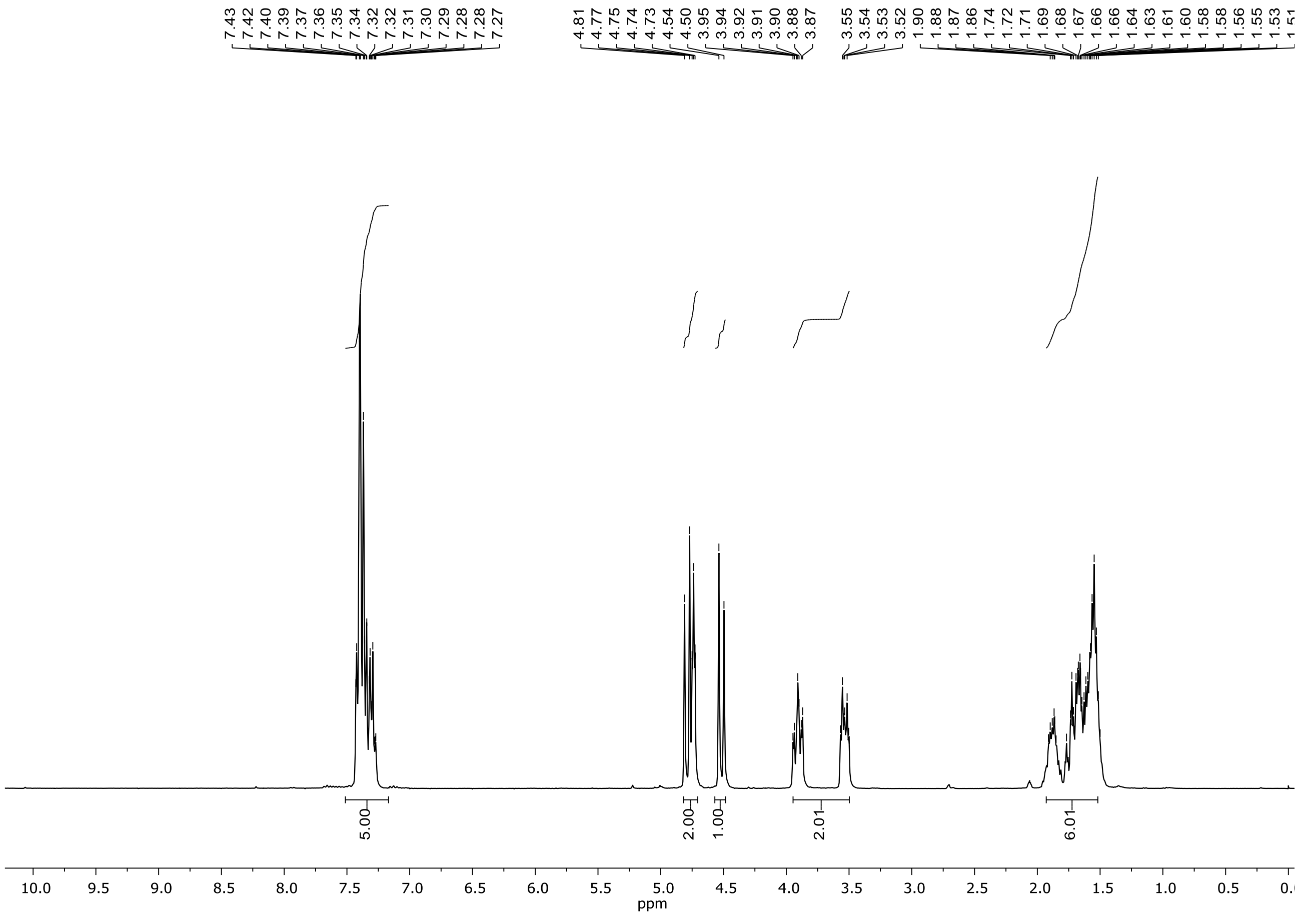

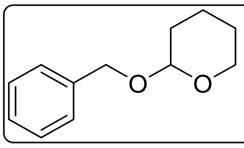

6 $^{13}C\{^{1}H\}$-NMR (75 MHz, acetone-d_6)

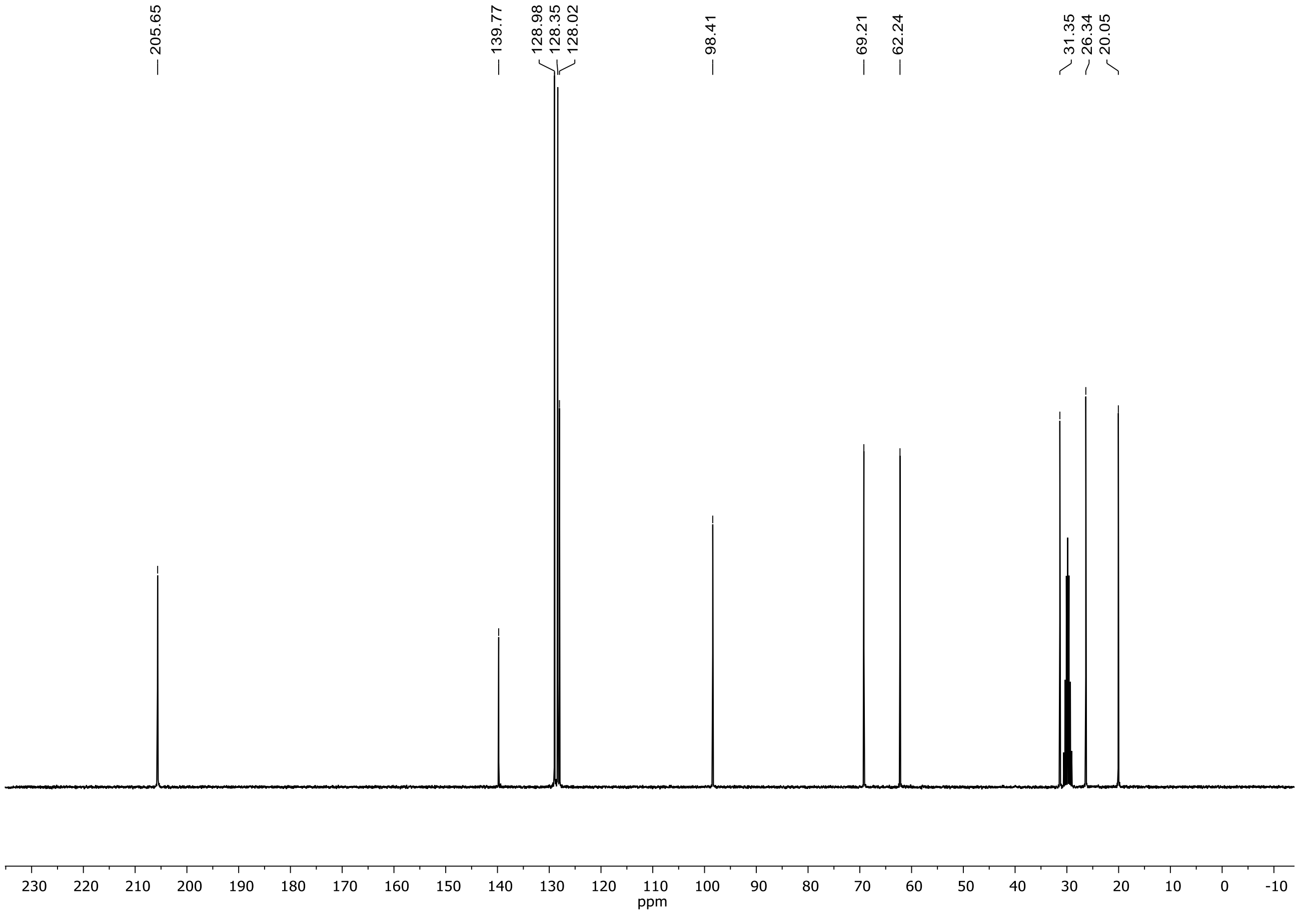

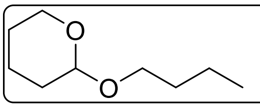

7

^{1}H-NMR (300 MHz, acetone-*d*$_6$)

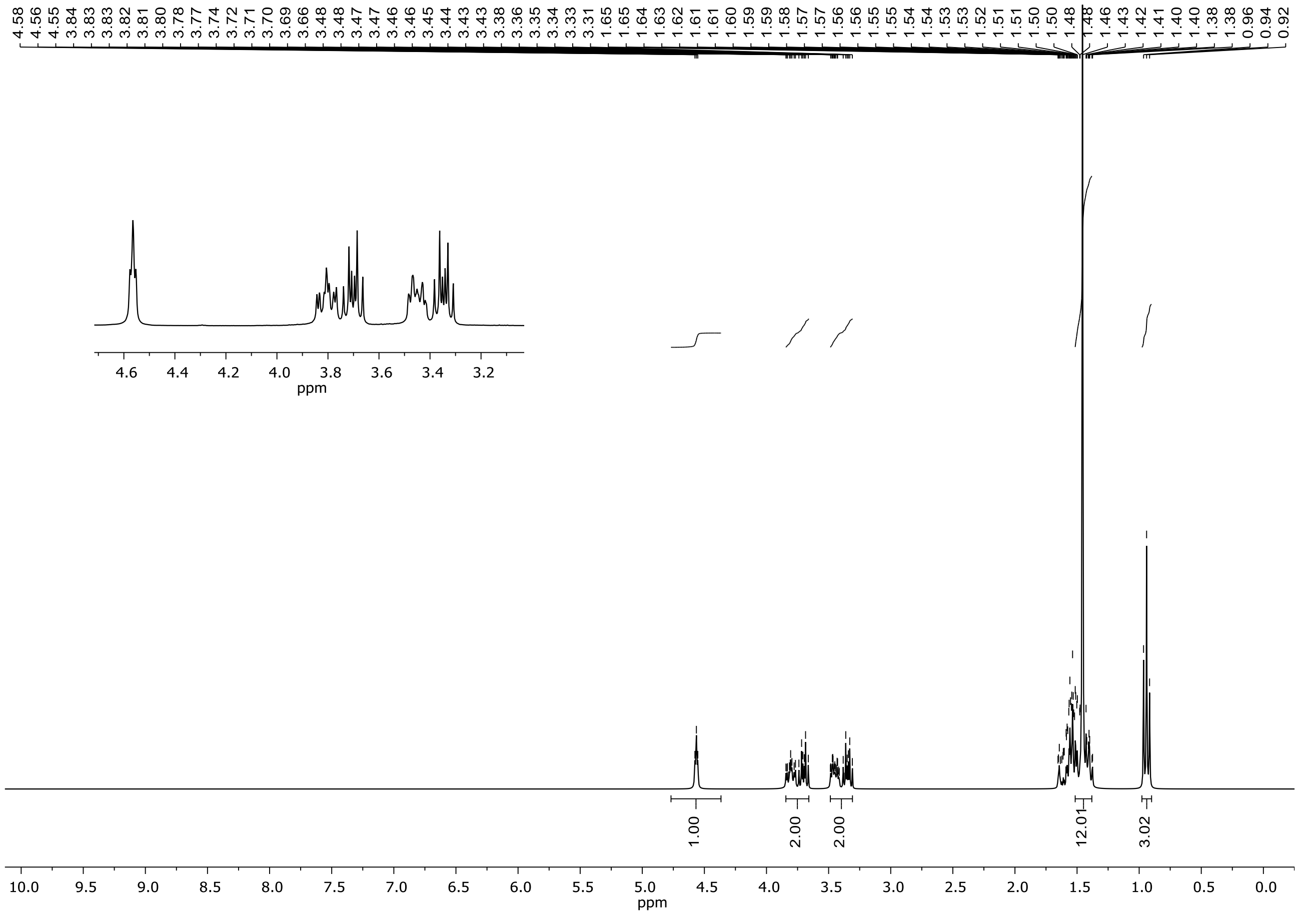

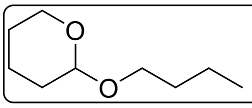

7

$^{13}C\{^{1}H\}$-NMR (75 MHz, acetone-d_6)

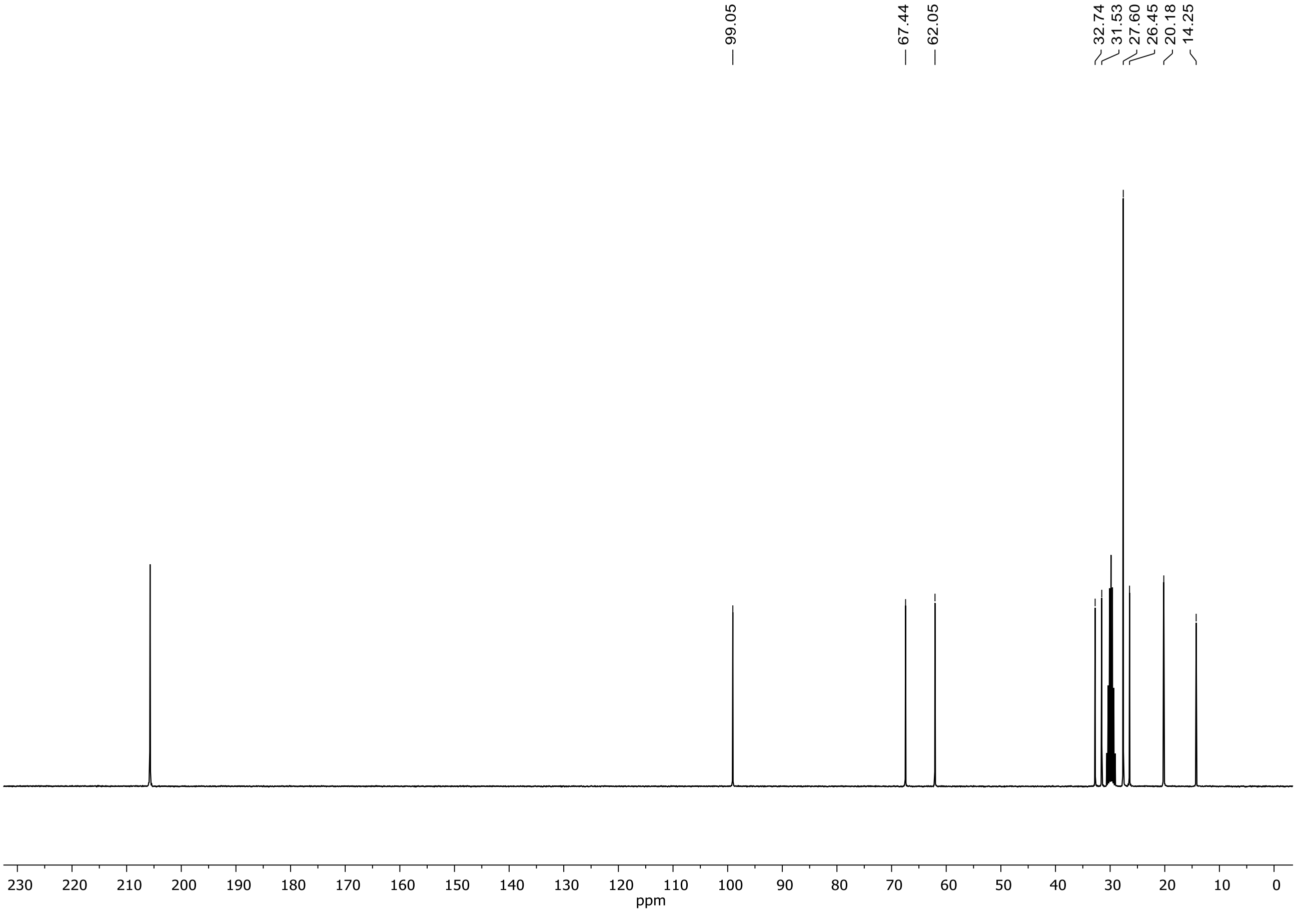

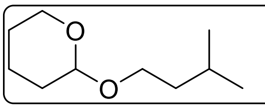

8 ^{1}H-NMR (300 MHz, acetone-d_6)

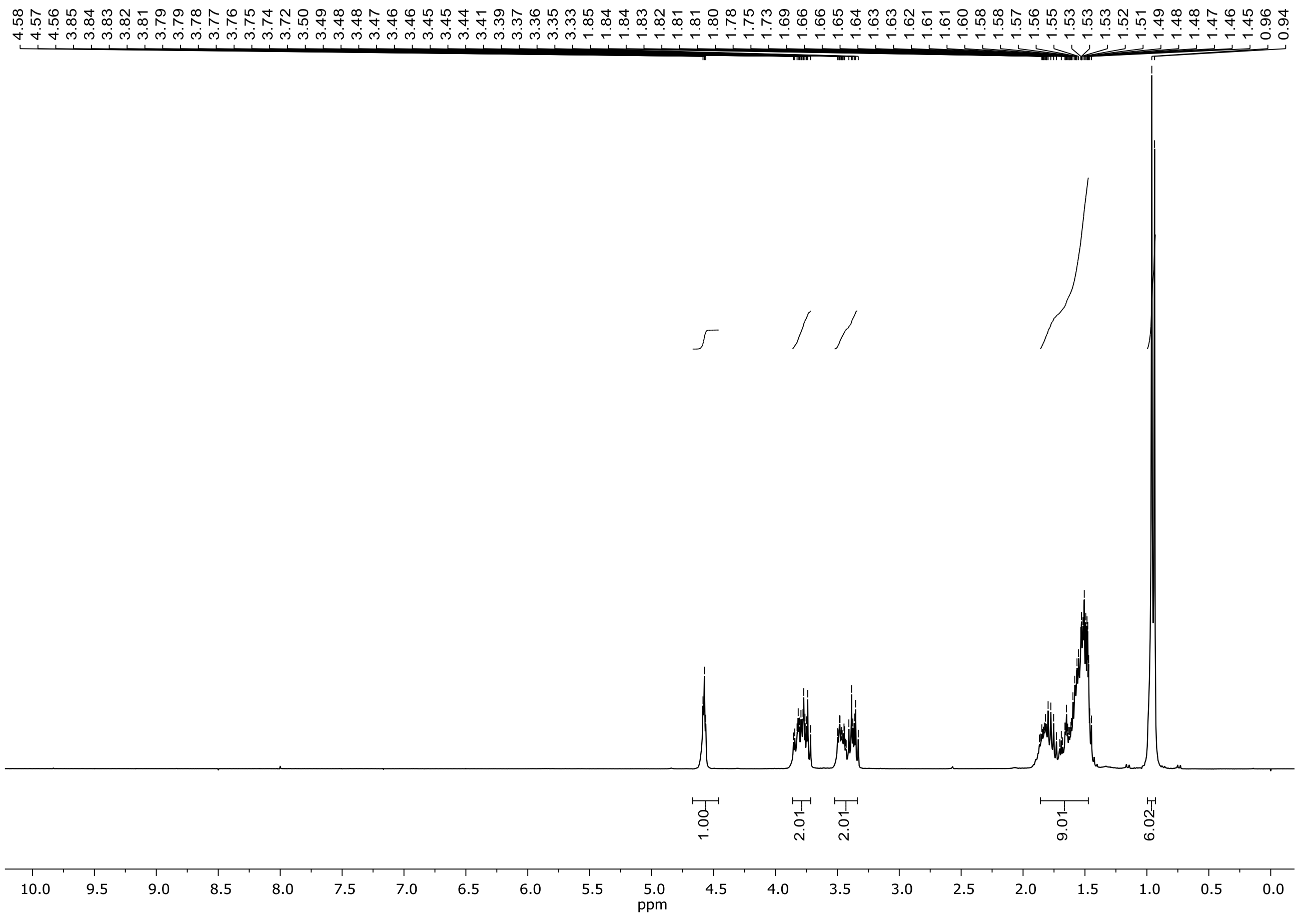

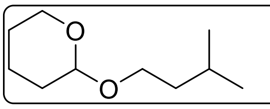

8 $^{13}C\{^{1}H\}$-NMR (75 MHz, acetone-d_6)

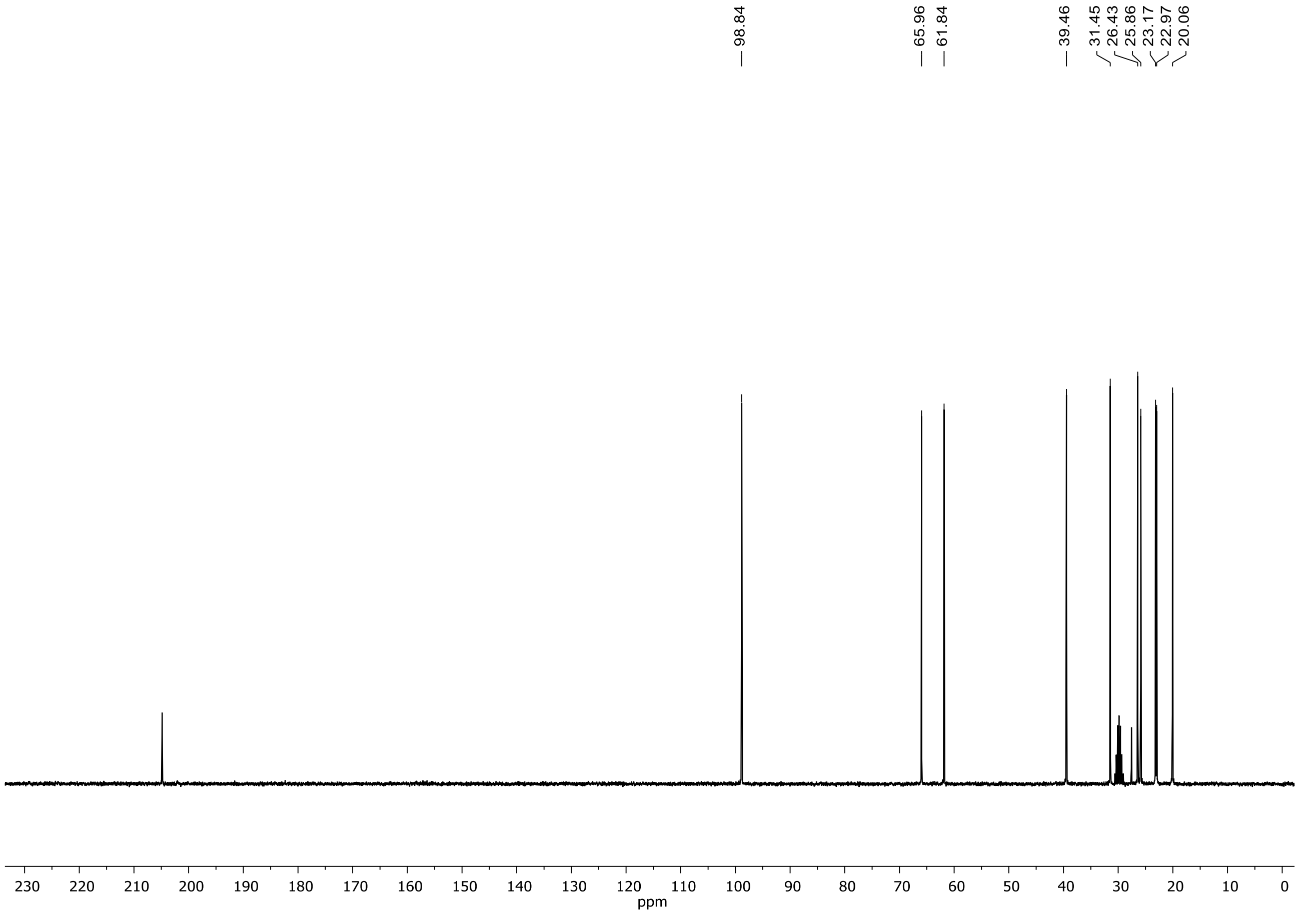

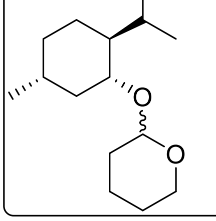

9

^{1}H-NMR (300 MHz, acetone-d_6)

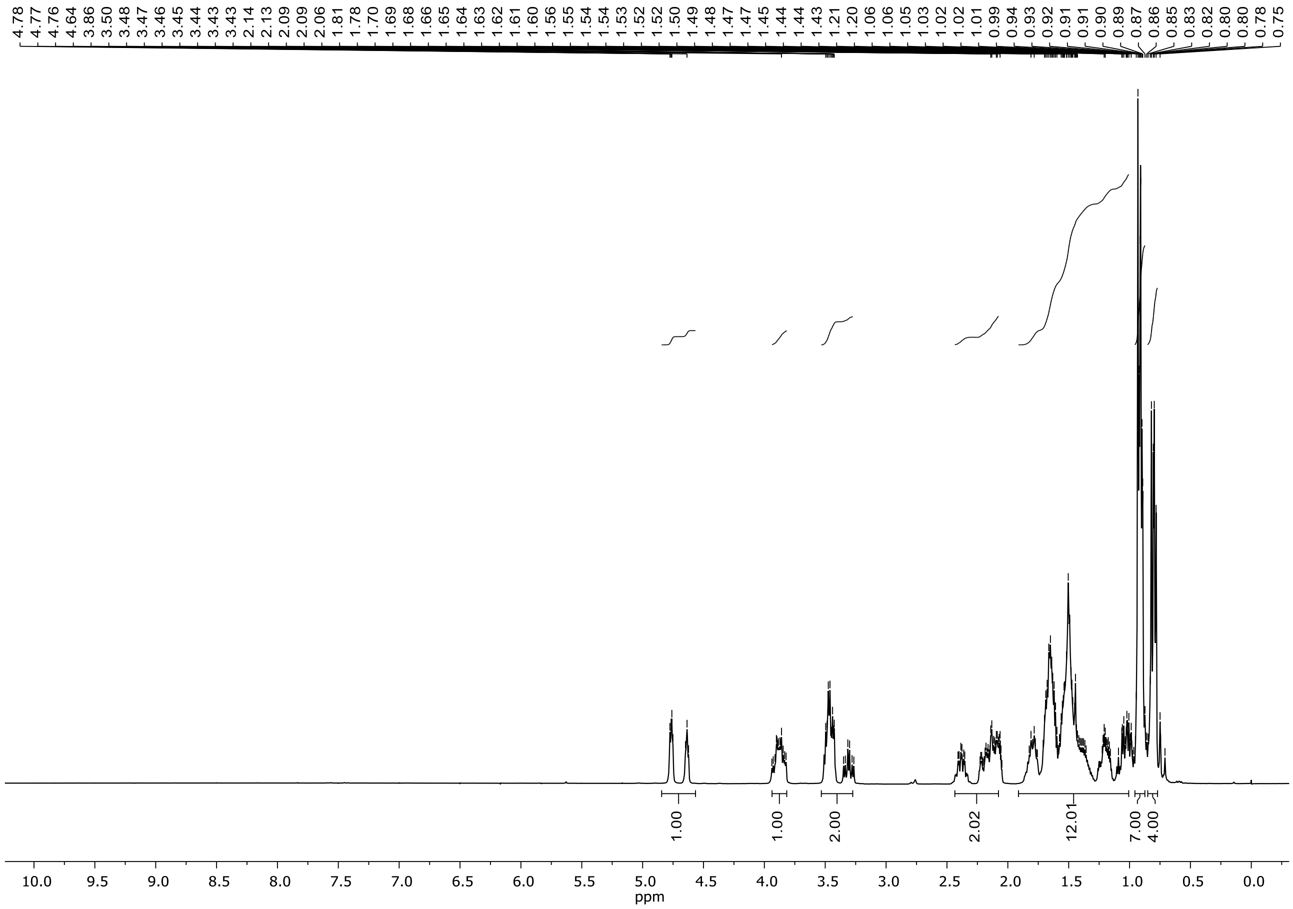

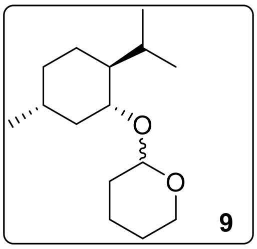

$^{13}C\{^{1}H\}$-NMR (75 MHz, acetone-d_6)

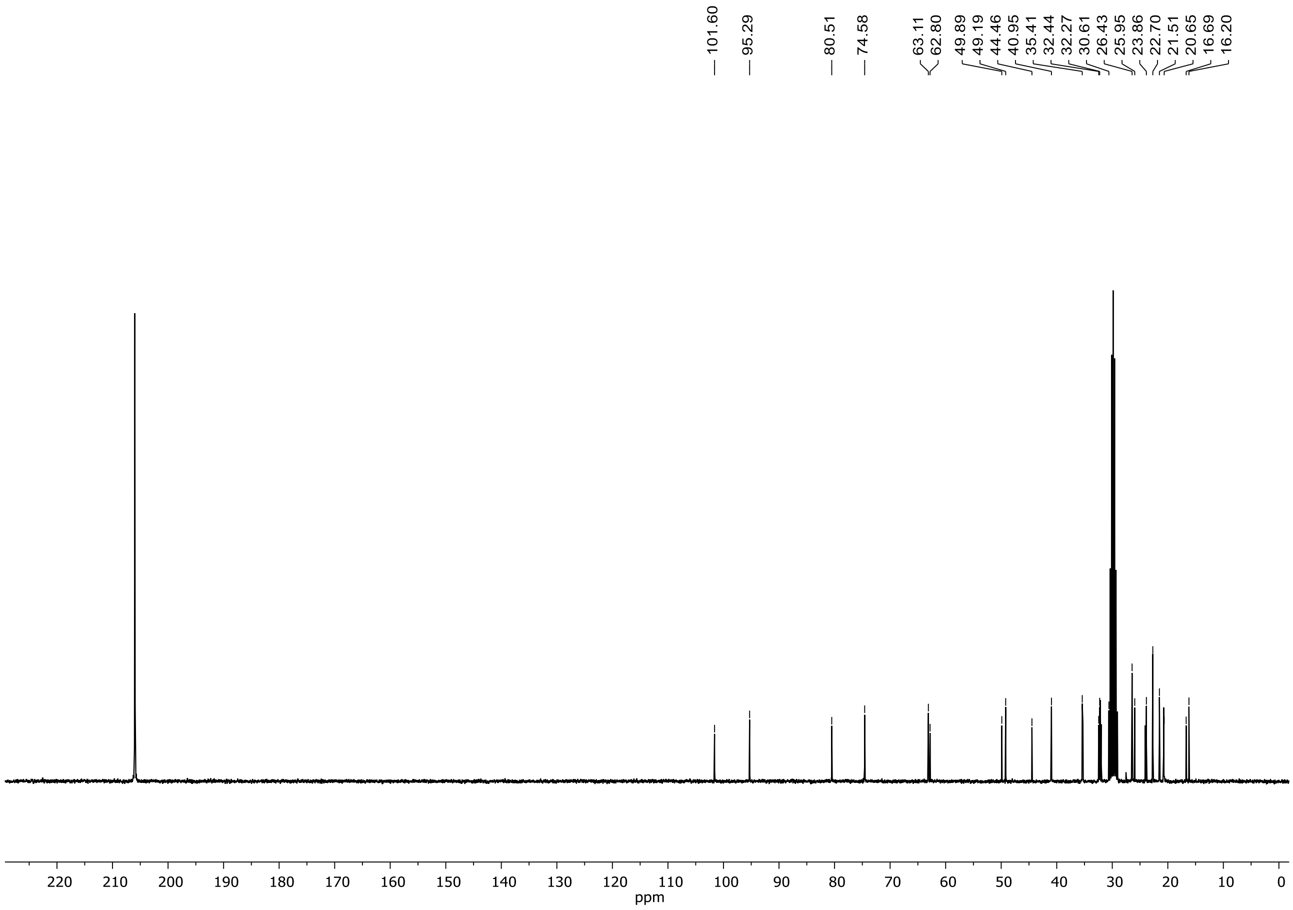

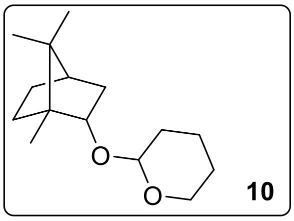

^{1}H-NMR (300 MHz, acetone-d_6)

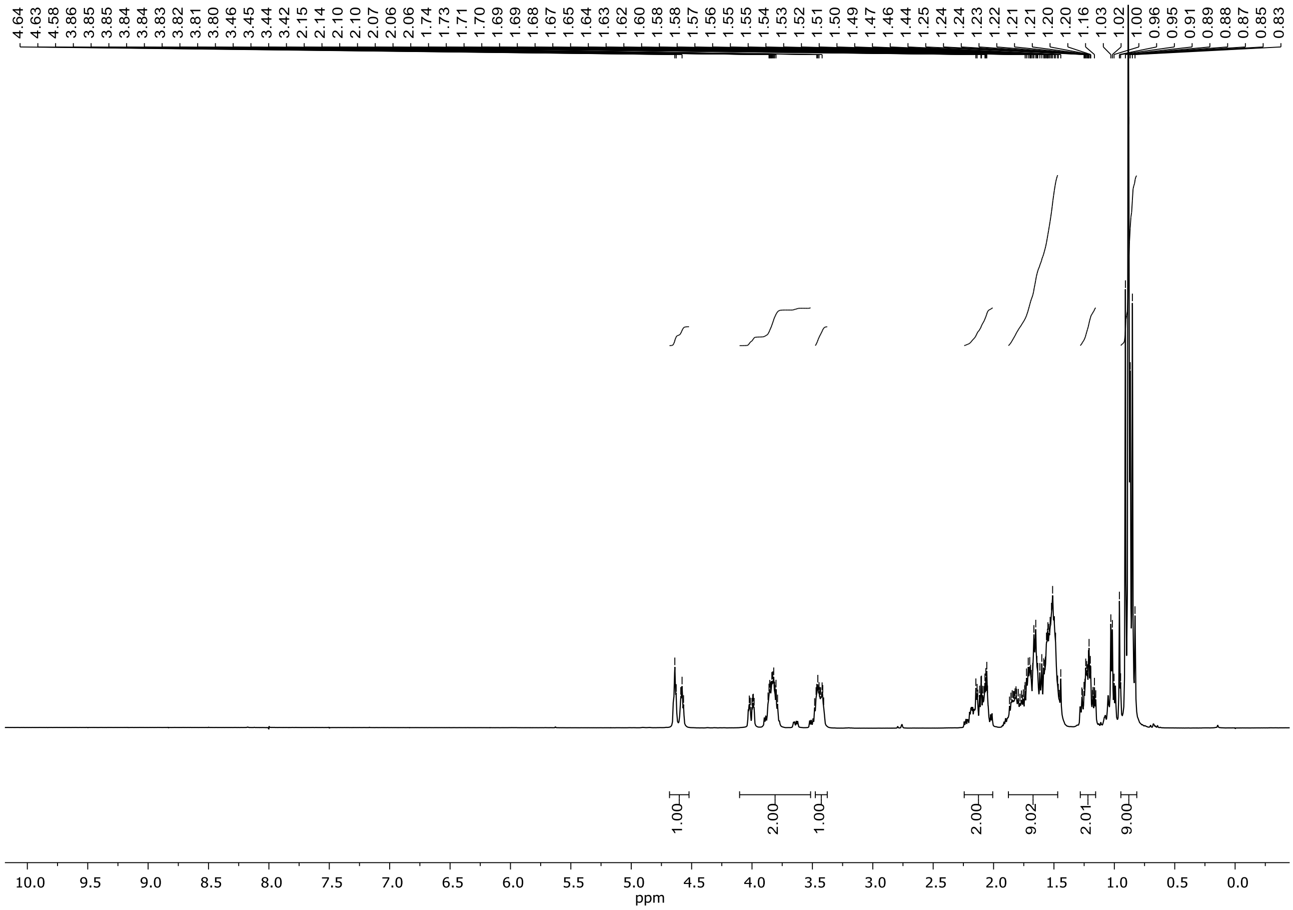

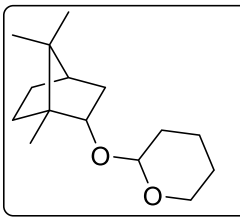

10

$^{13}C\{^{1}H\}$-NMR (75 MHz, acetone-d_6)

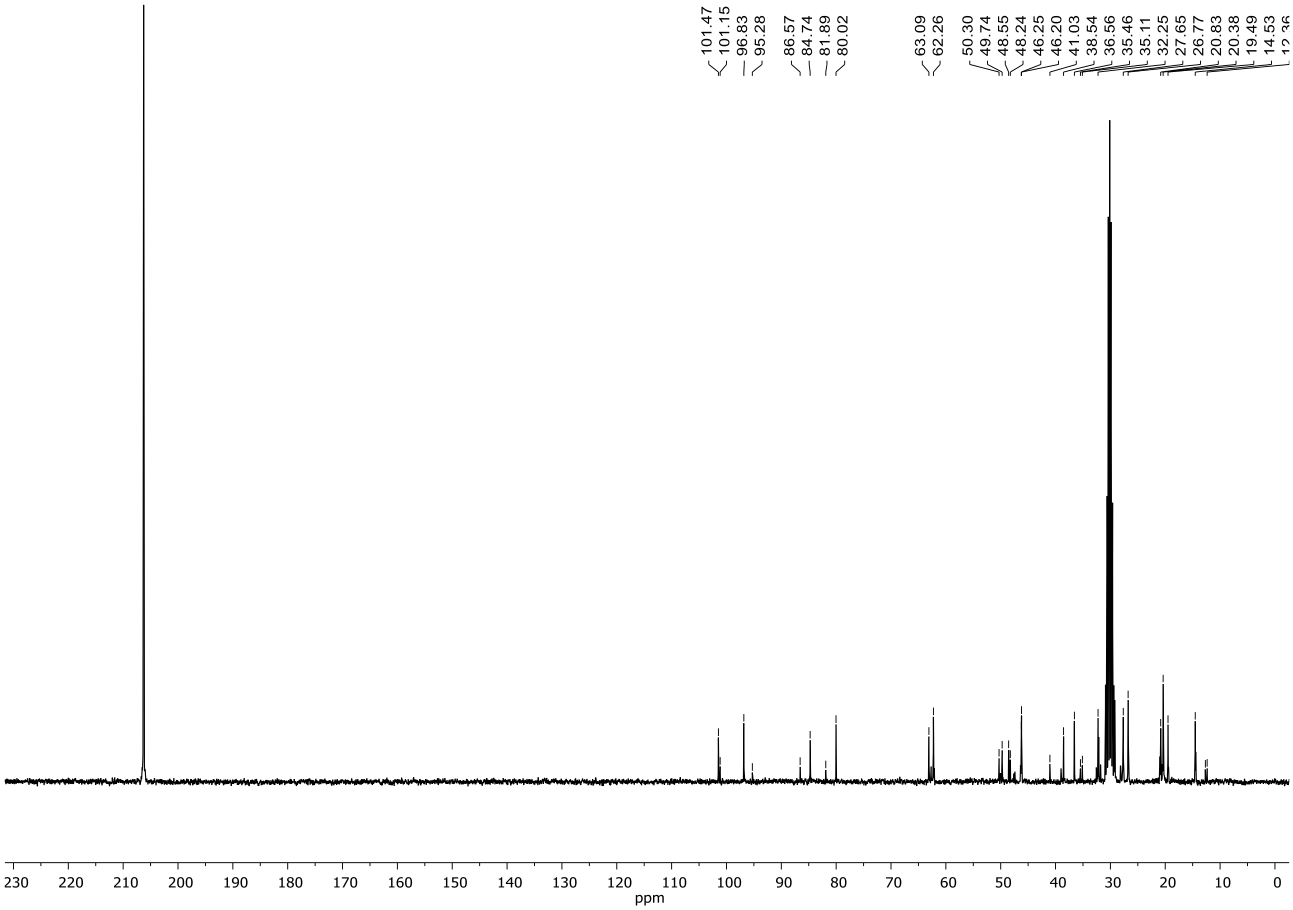

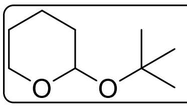

11 ^{1}H-NMR (300 MHz, acetone-d_6)

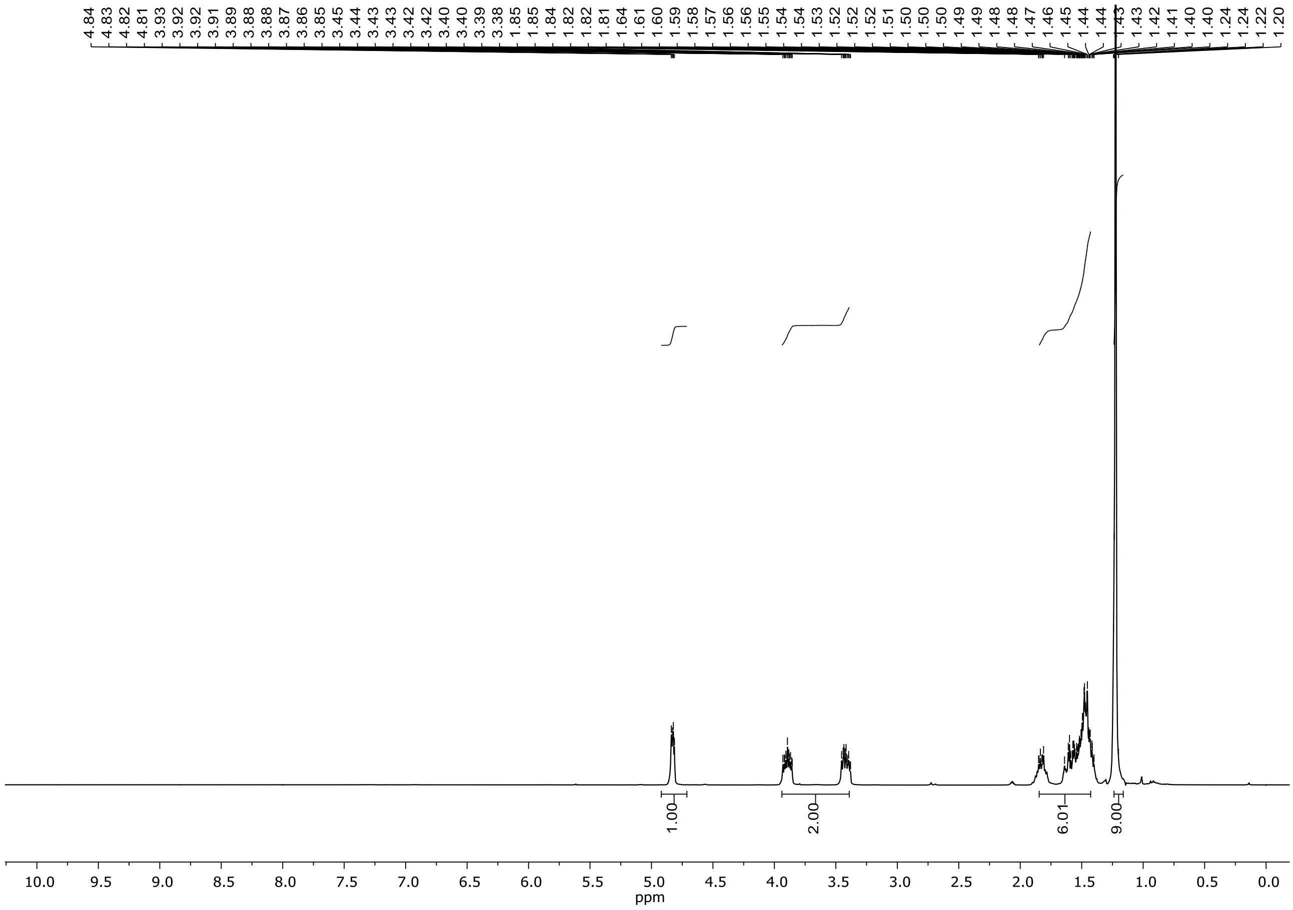

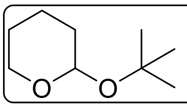

11 $^{13}C\{^{1}H\}$-NMR (75 MHz, acetone-d_6)

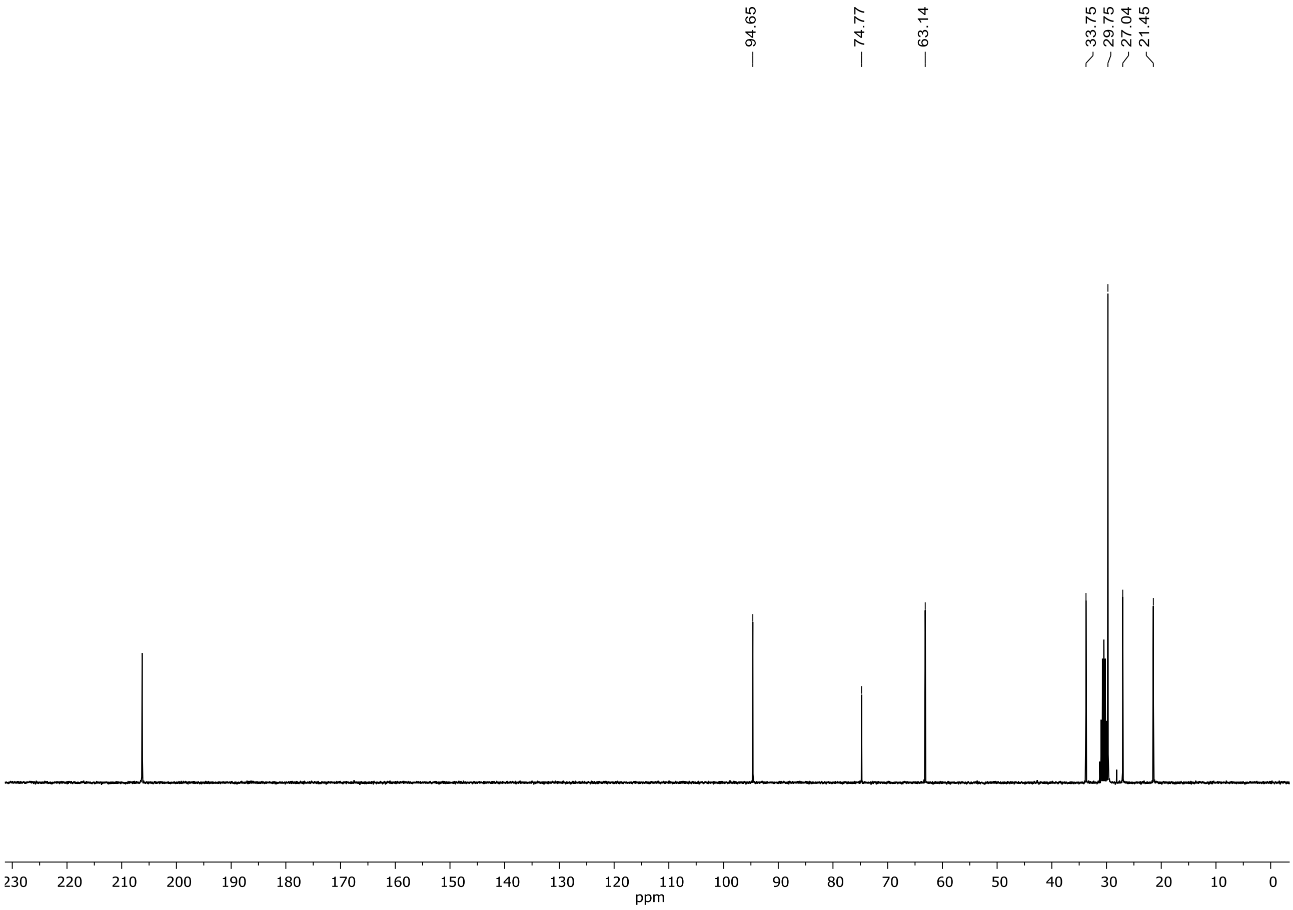

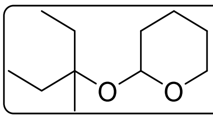

12 ^{1}H-NMR (300 MHz, acetone-d_6)

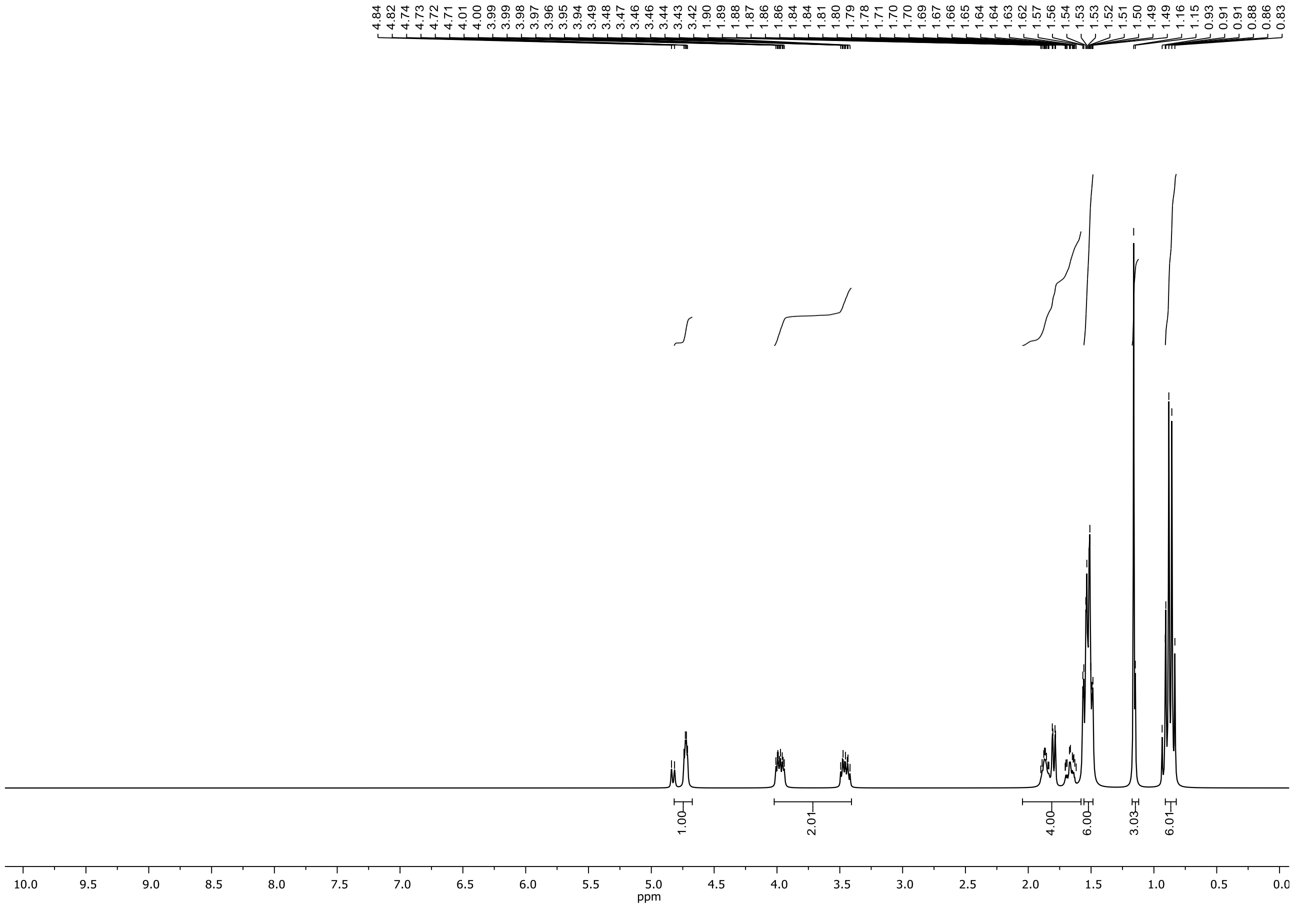

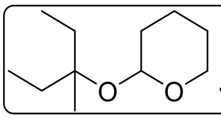

12 $^{13}C\{^{1}H\}$-NMR (75 MHz, acetone-d_6)

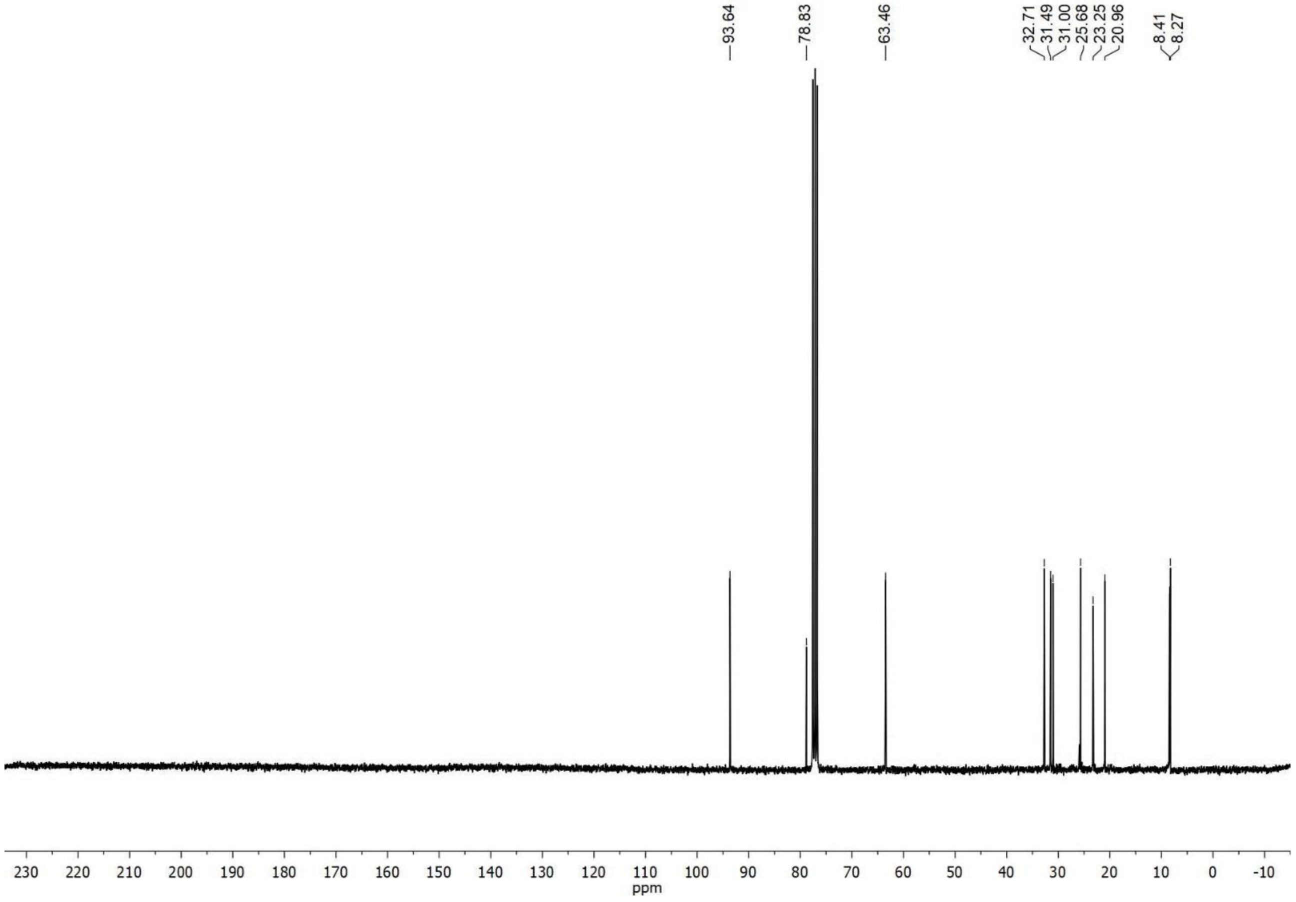

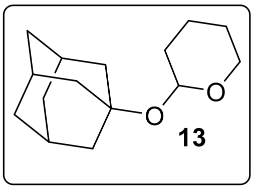

^{1}H-NMR (300 MHz, acetone-d_6)

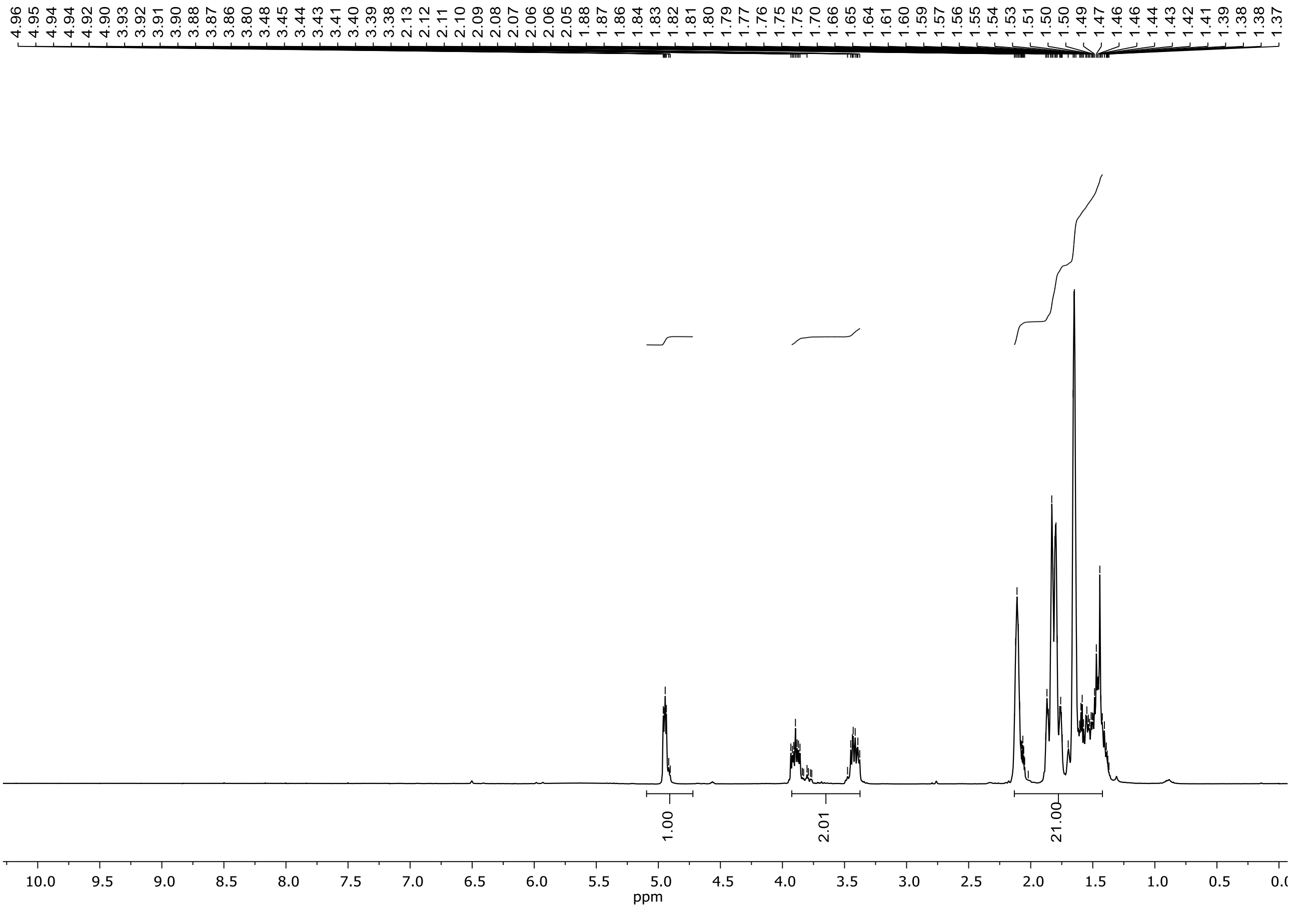

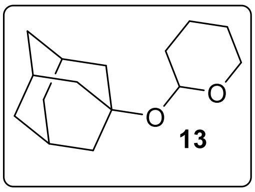

$^{13}C\{^{1}H\}$-NMR (75 MHz, acetone-d_6)

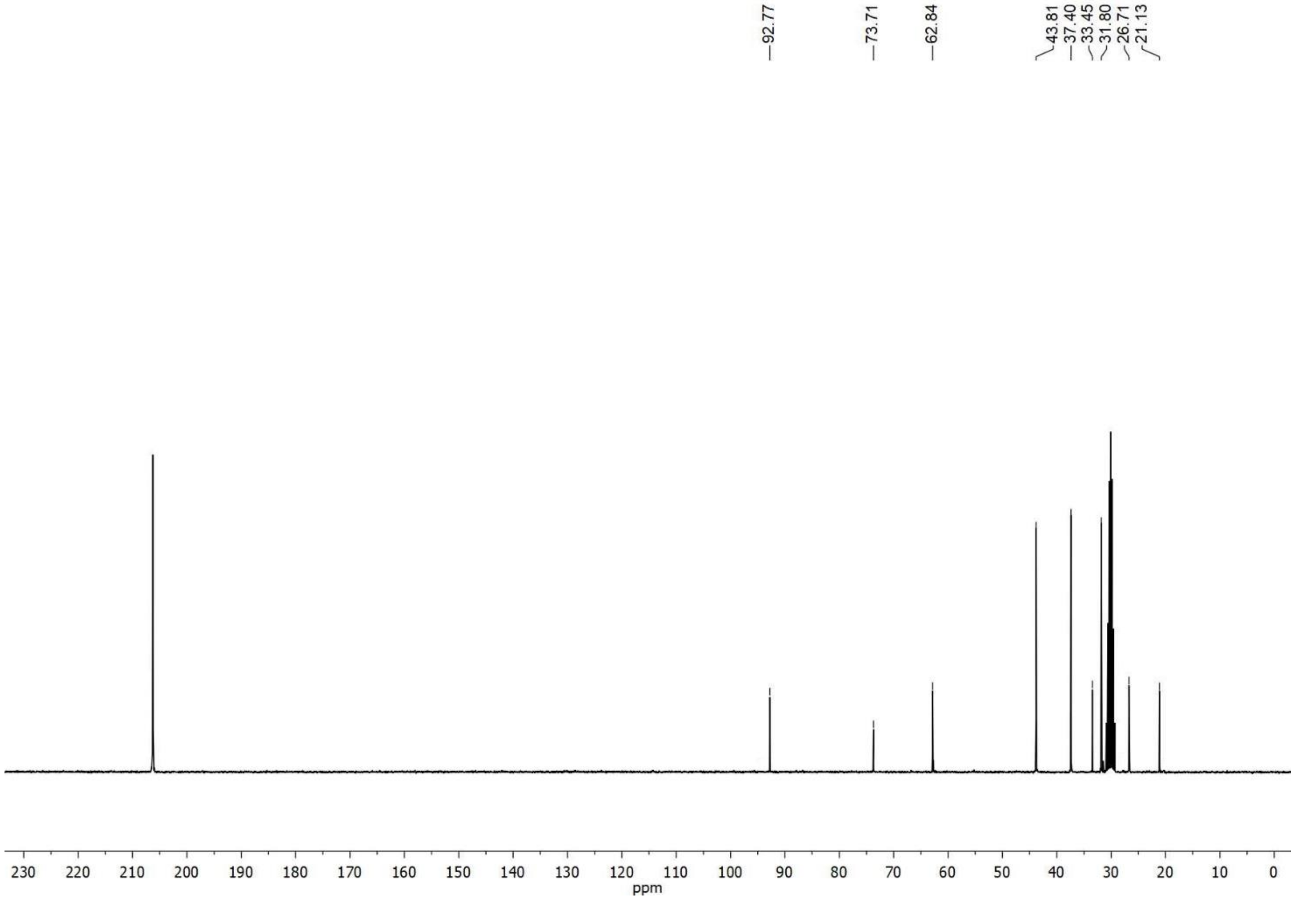

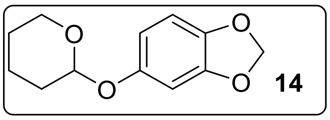

^{1}H-NMR (300 MHz, acetone-d_6)

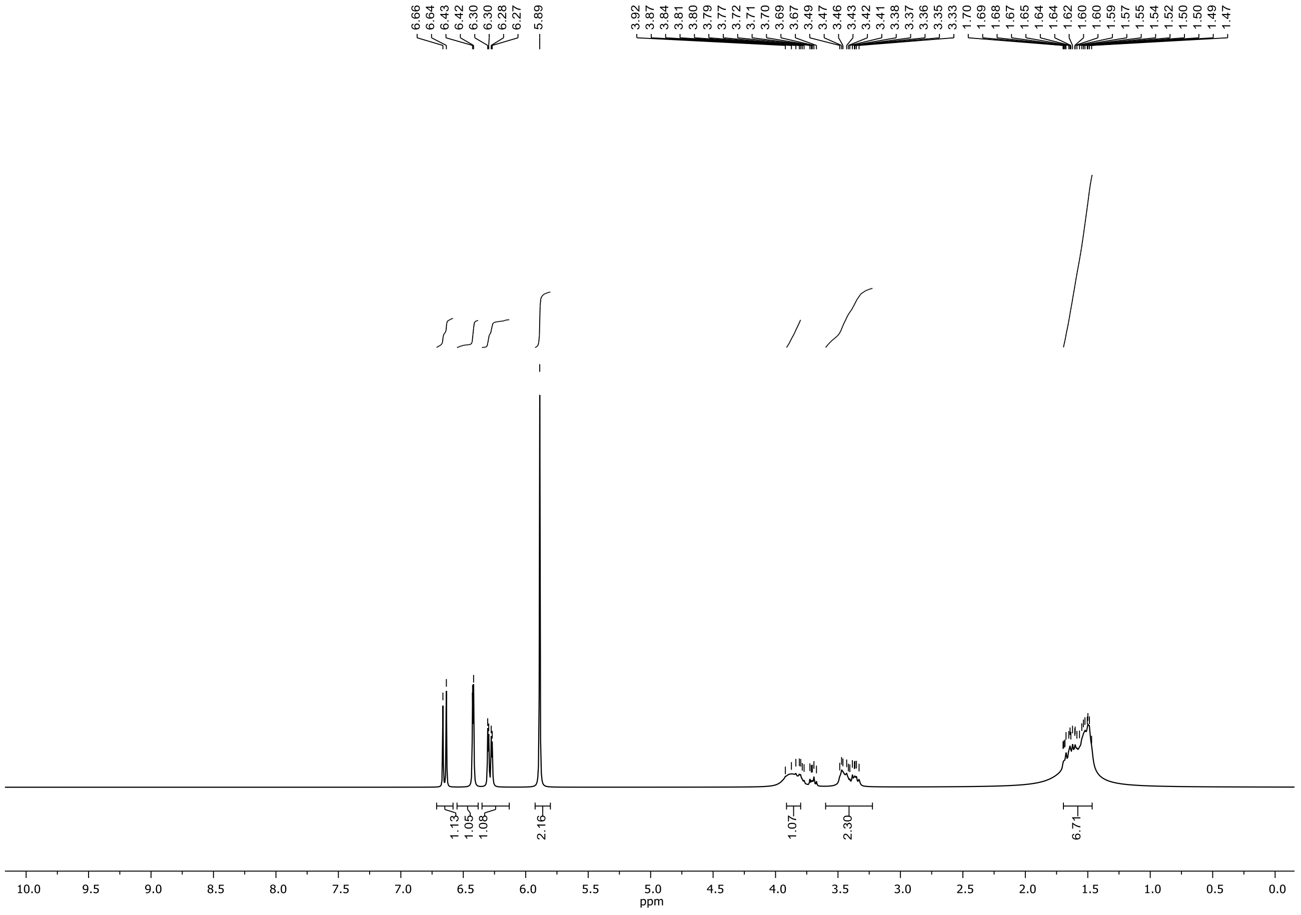

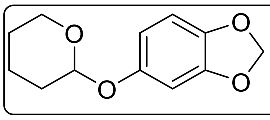 **14** $^{13}C\{^1H\}$-NMR (75 MHz, acetone-d_6)

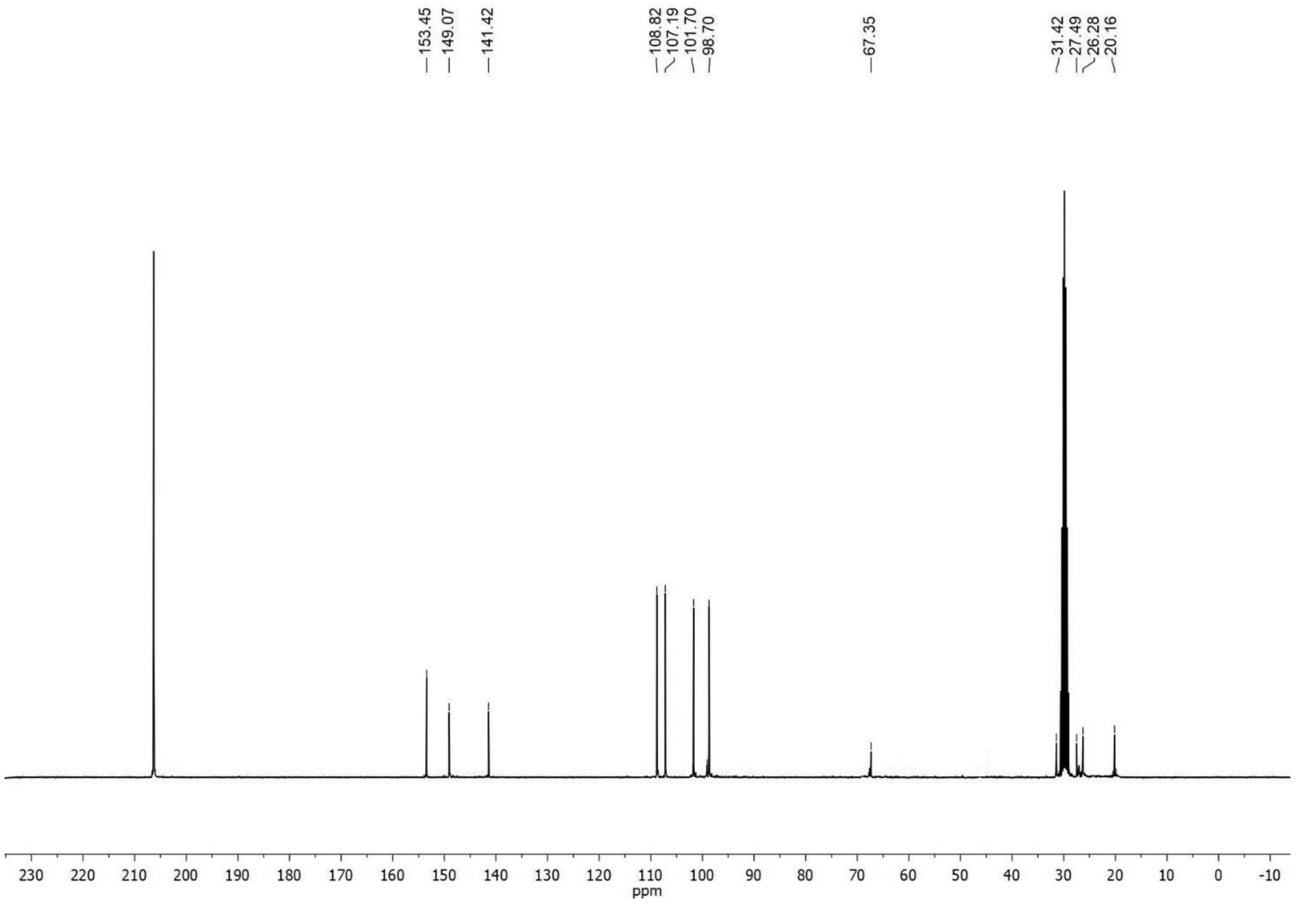

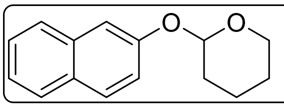

15 ^{1}H-NMR (300 MHz, acetone-d_6)

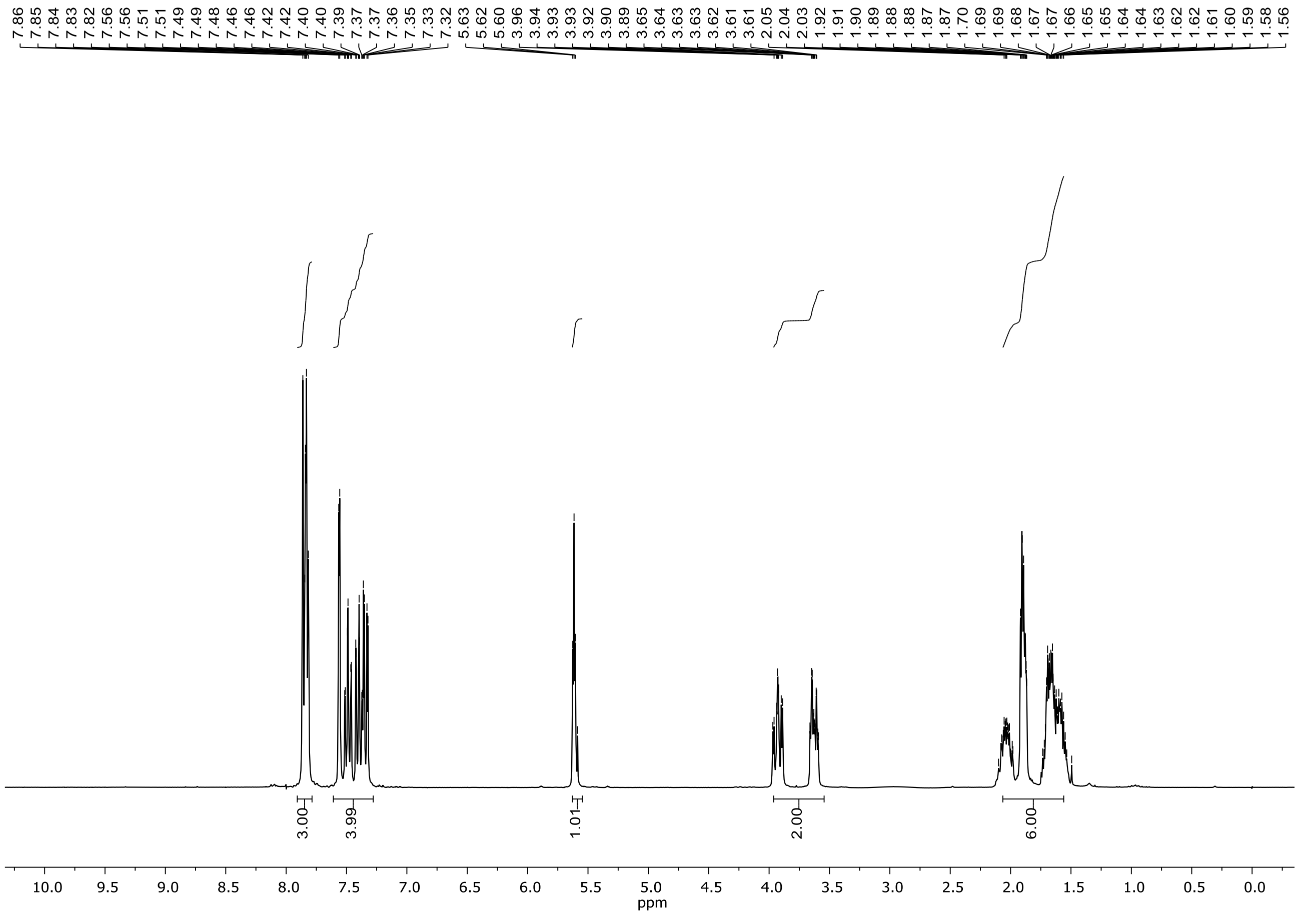

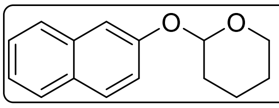

15

$^{13}C\{^{1}H\}$-NMR (75 MHz, acetone-d_6)

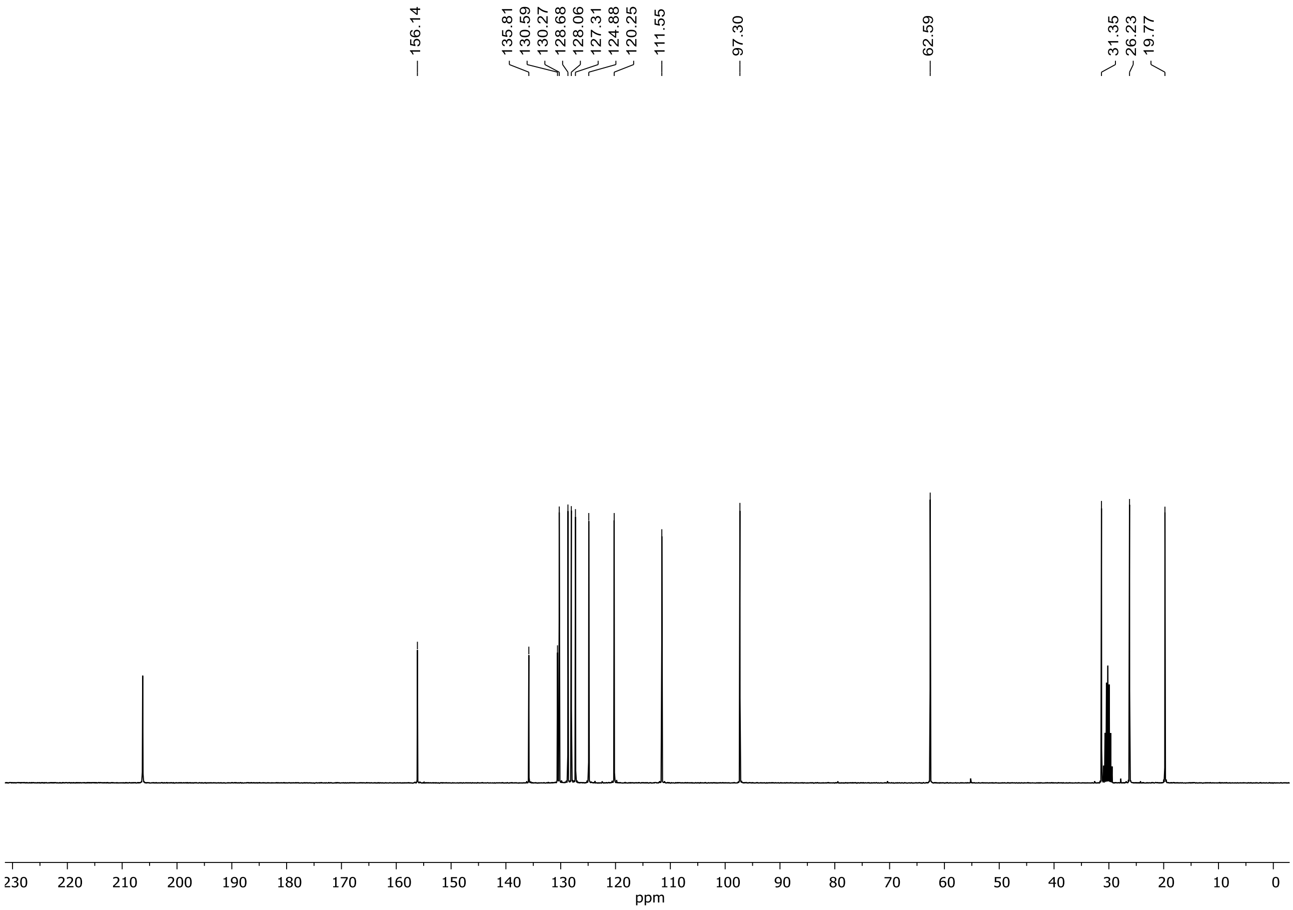

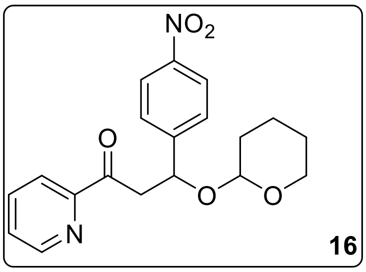

16 ^{1}H-NMR (300 MHz, acetone-d_6)

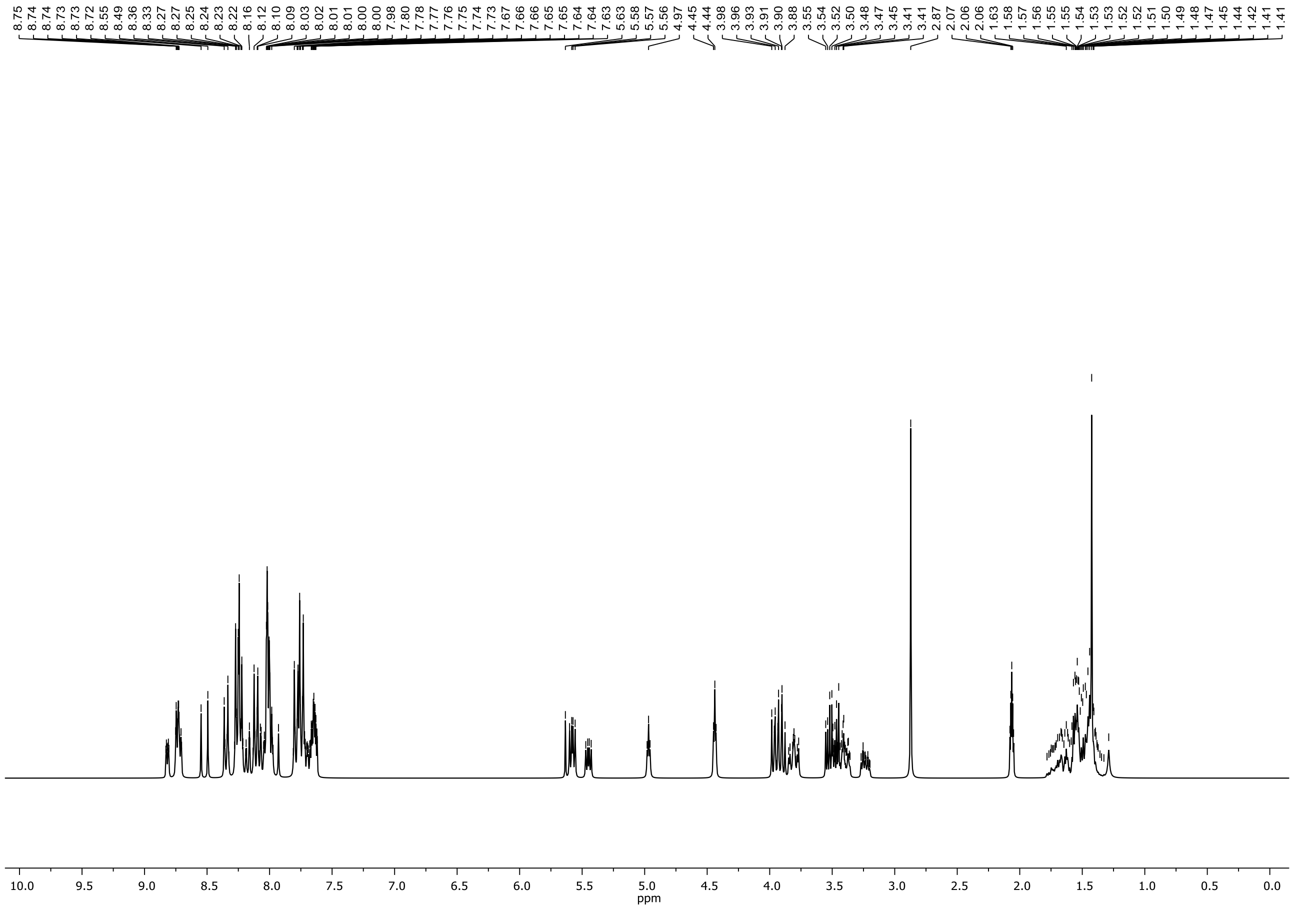

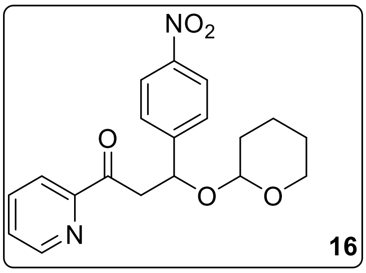

$^{13}C\{^{1}H\}$-NMR (75 MHz, acetone-d_6)

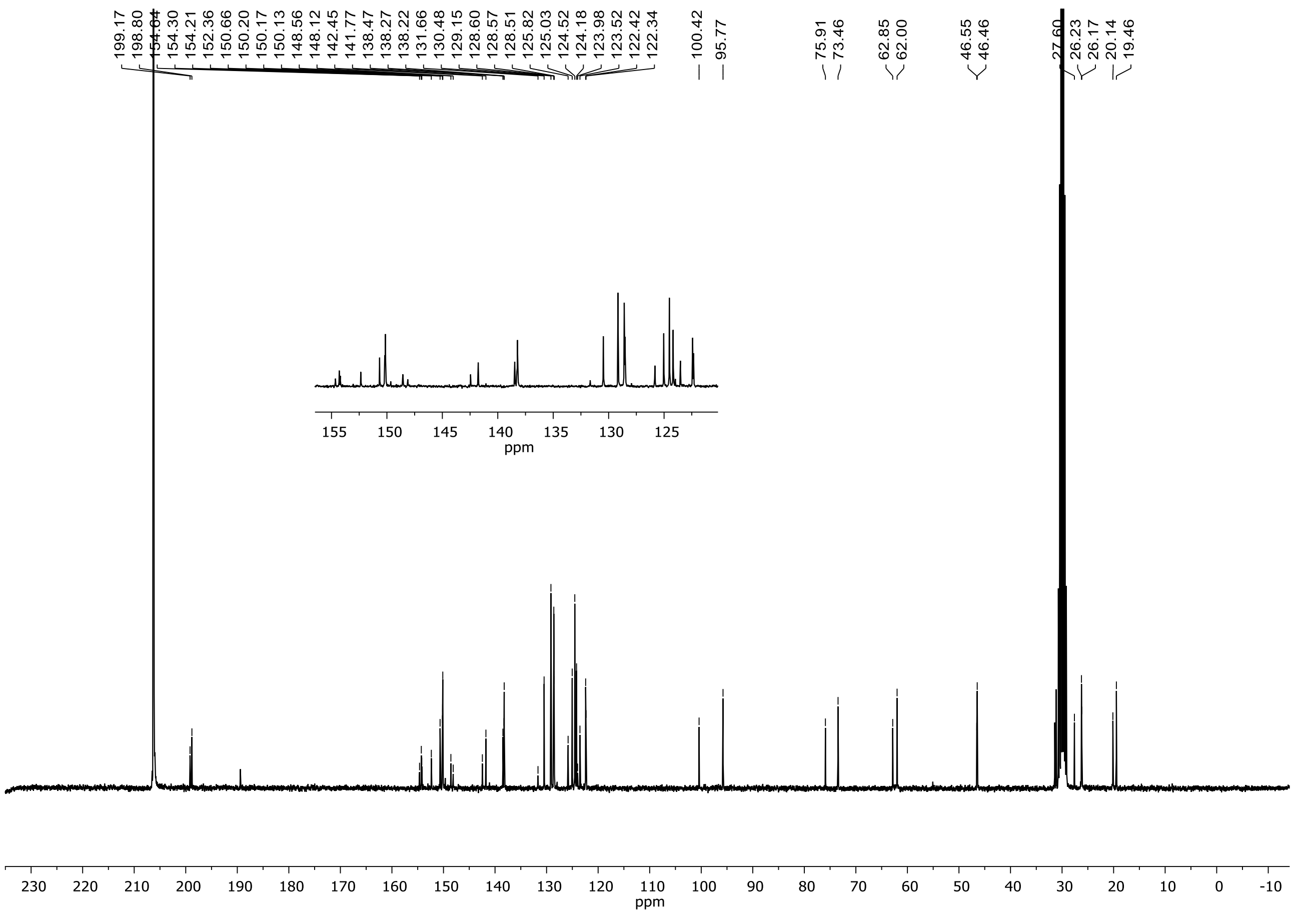

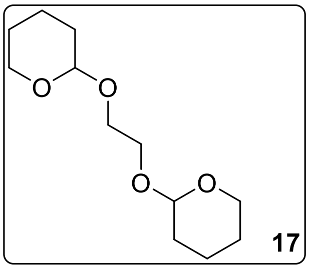

17 ^{1}H-NMR (300 MHz, acetone-d_6)

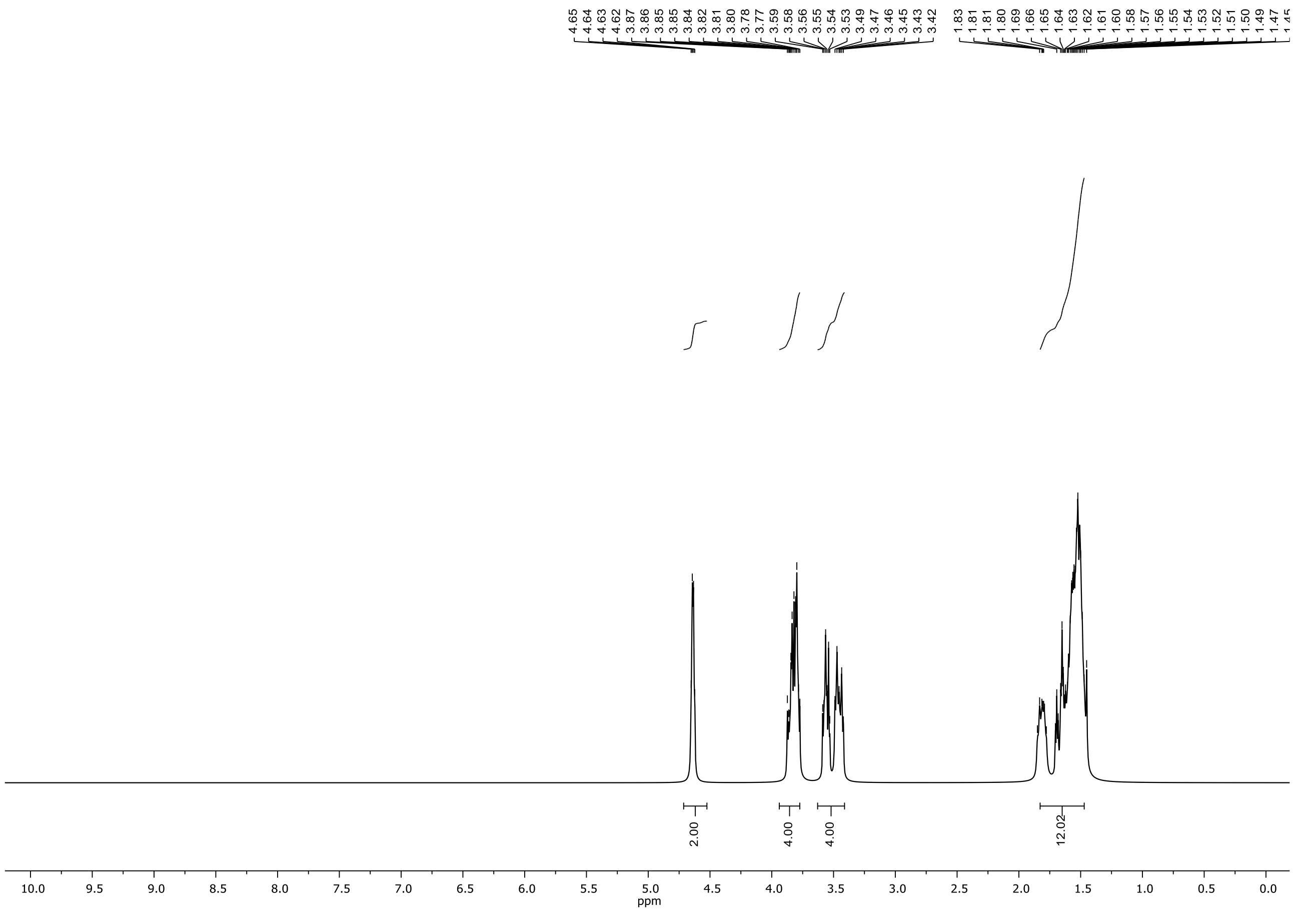

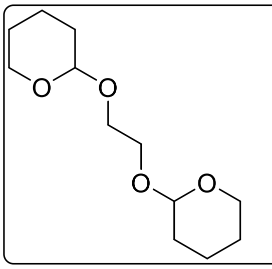

17 $^{13}C\{^1H\}$-NMR (75 MHz, acetone-d_6)

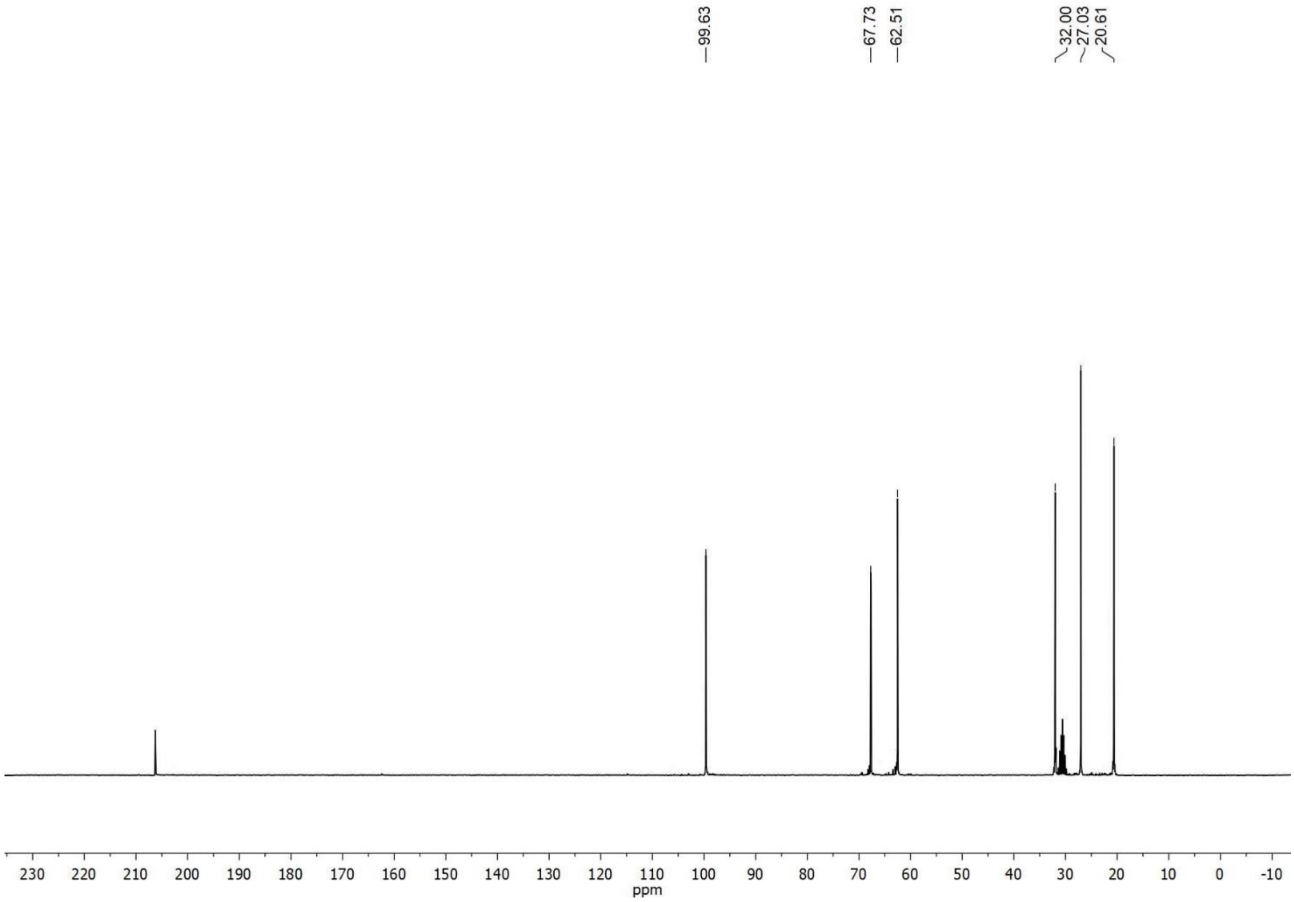

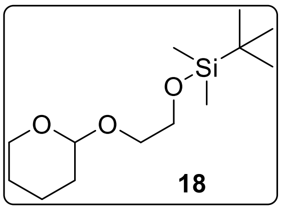

^{1}H-NMR (300 MHz, acetone-d_6)

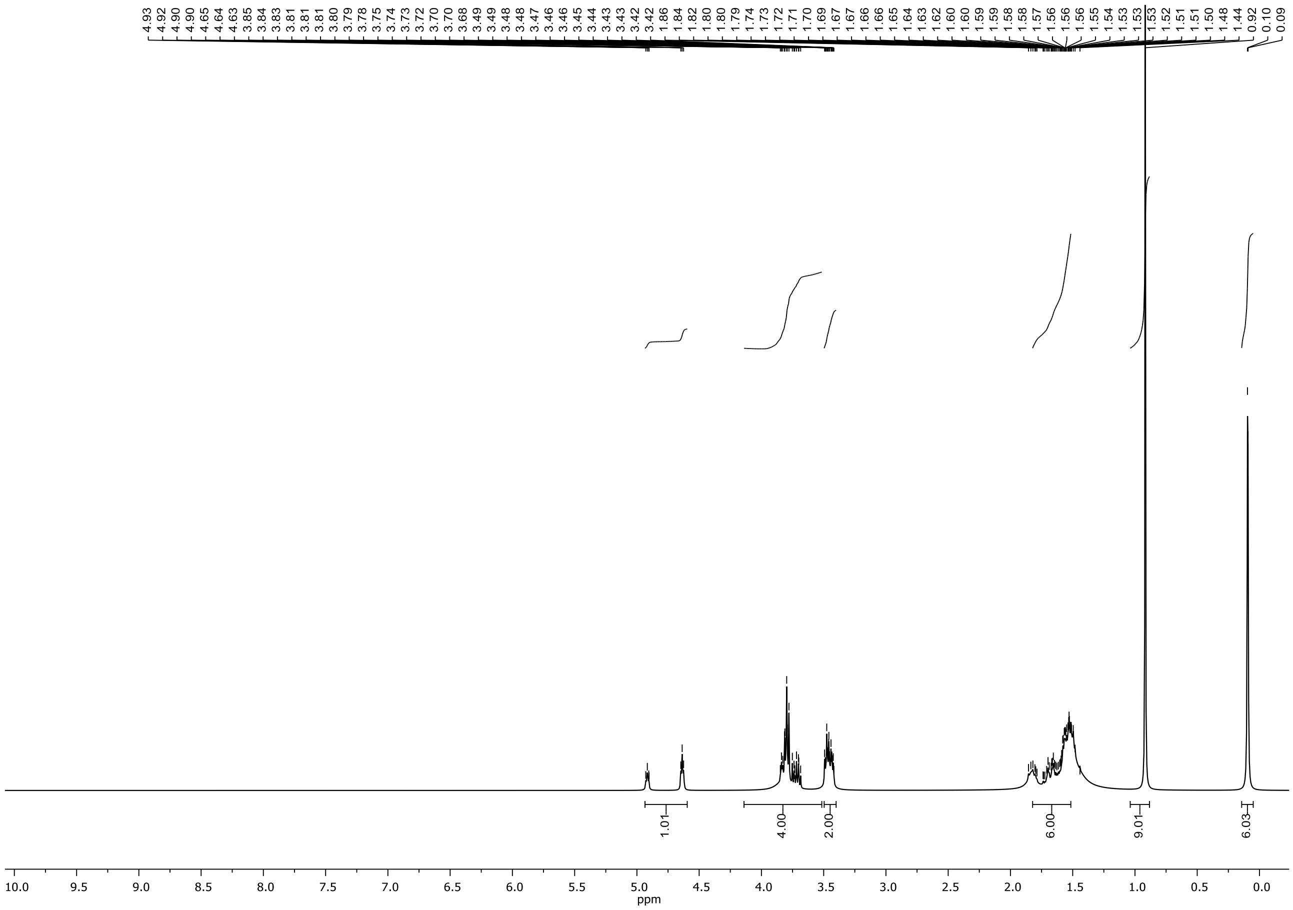

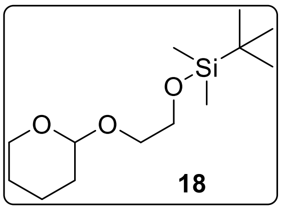

18

$^{13}C\{^{1}H\}$-NMR (75 MHz, acetone-d_6)

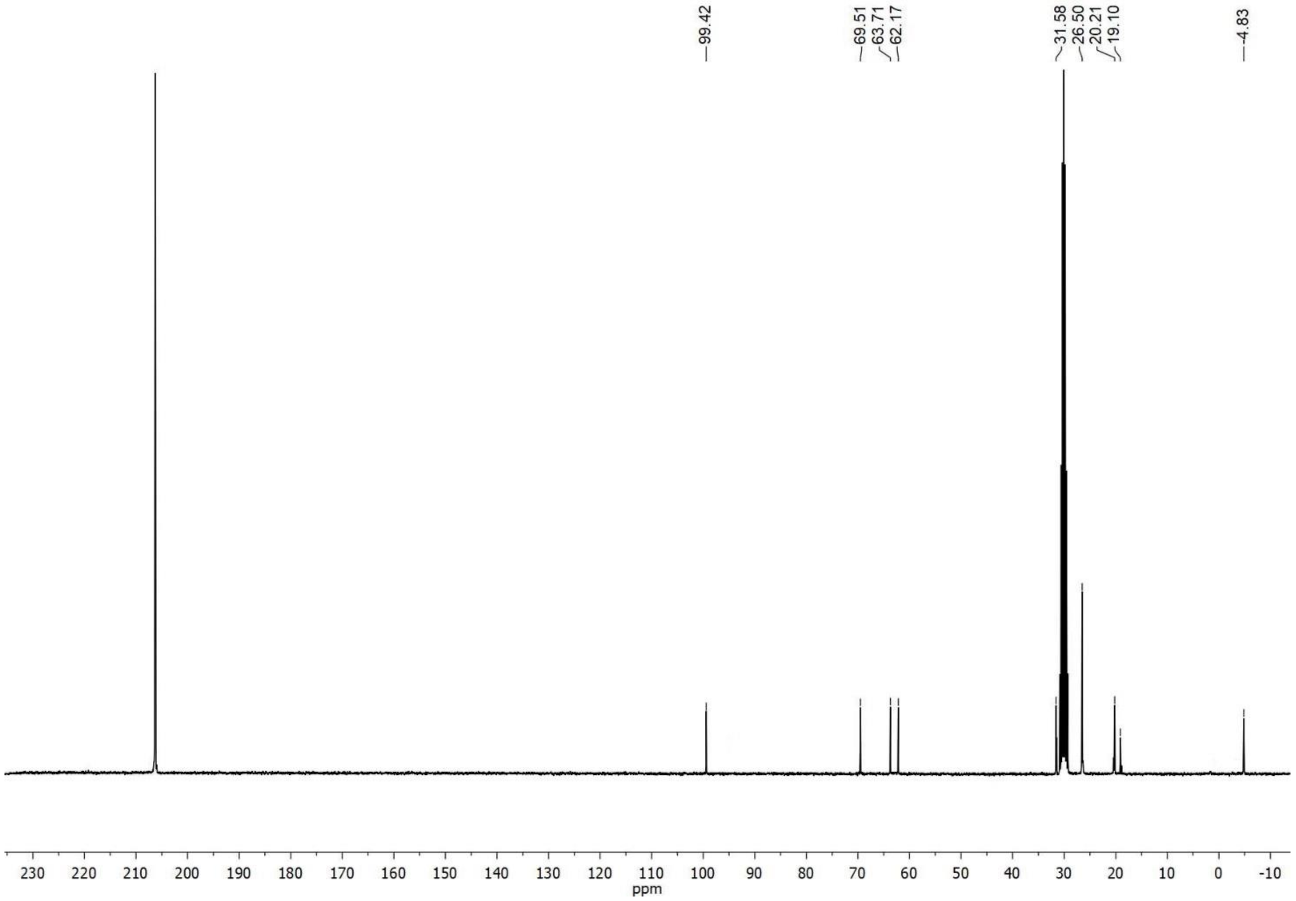

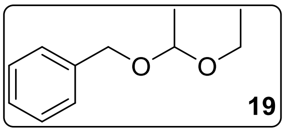

19 ^{1}H-NMR (300 MHz, acetone-d_6)

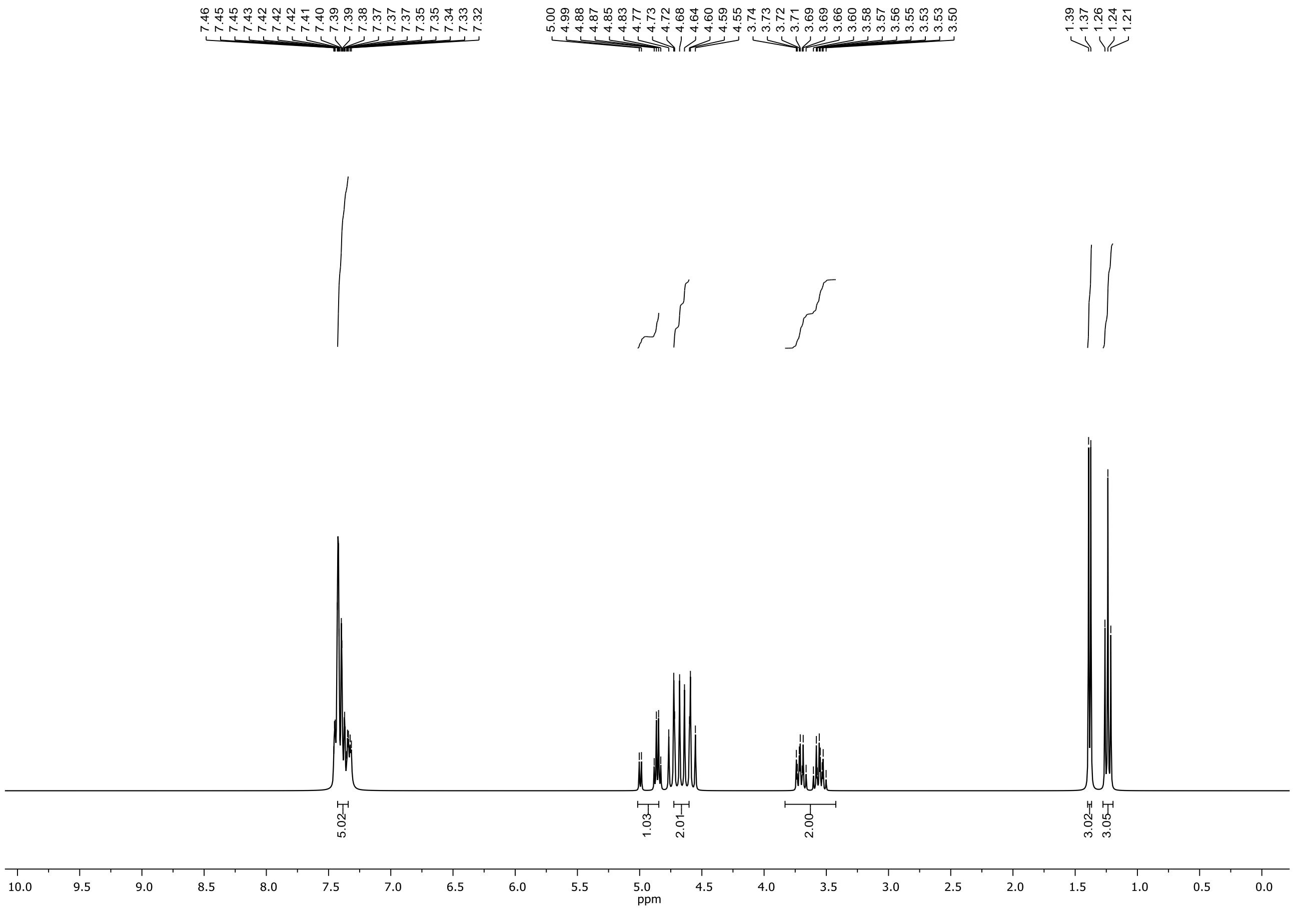

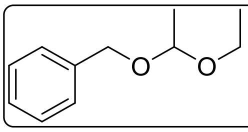

19 $^{13}C\{^1H\}$-NMR (75 MHz, acetone-d_6)

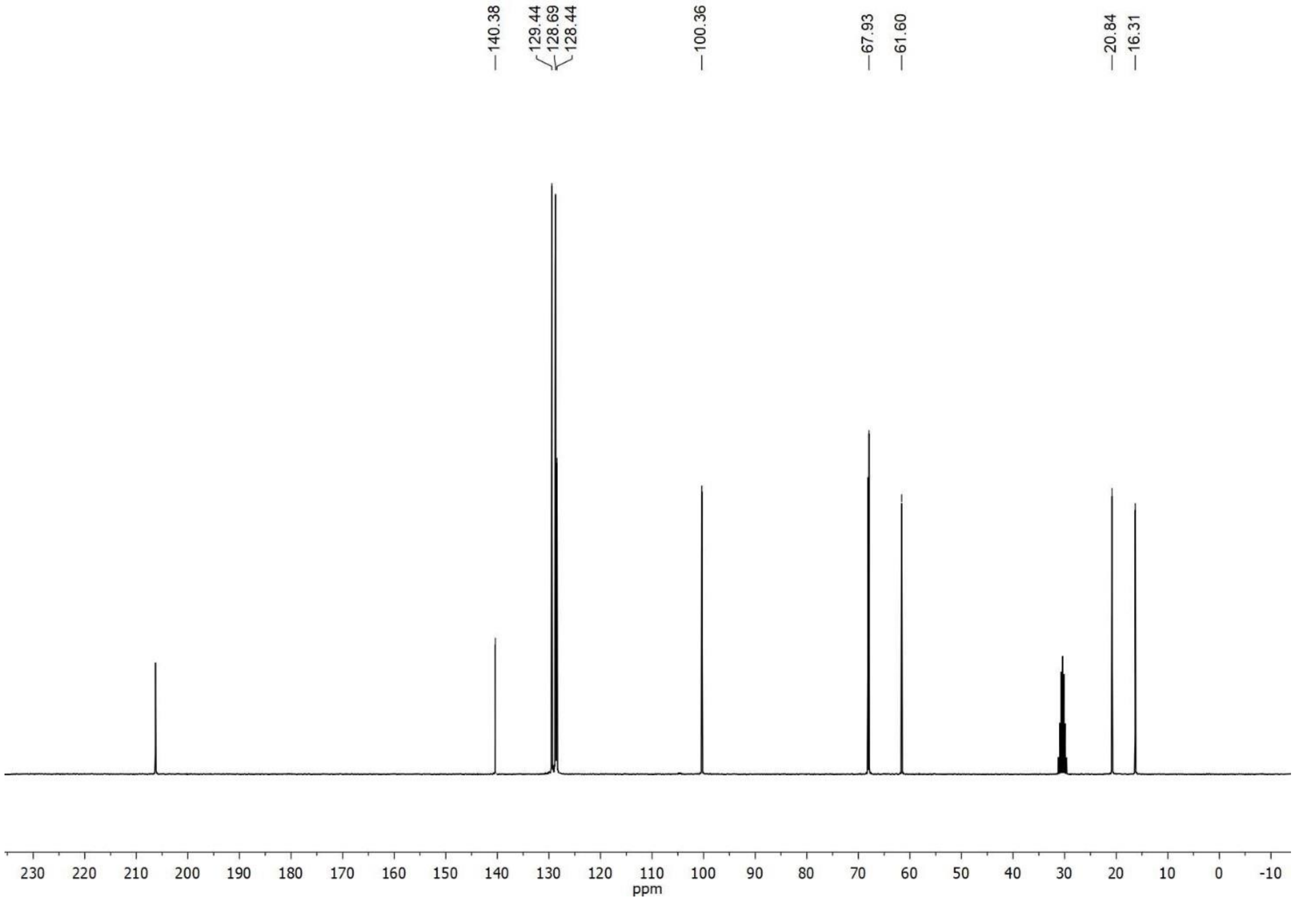

REFERENCES

(S1) Hanoian, P.; Sigala, P. A.; Herschlag, D.; Hammes-Schiffer S. *Biochemistry*, **2010**, *49*, 10339–10348.

(S2) Le Paih, J.; Dérien, S.; Demerseman, B.; Bruneau, Dixneuf, C.; Toupet, P. H. L.; Dazinger, G.; Kirchner K. *Chem. Eur. J.* **2005**, *11*, 1312–1324.

(S3) Sarju, J.; Danks, T. N.; Wagner, G. *Tetrahedron Lett.* **2004**, *45*, 7675–7677.

(S4) Mazille, F.; Schoettl, T.; Lopez, A.; Pulgarin, C. *J. Photochem. & Photobiol. A: Chem.* **2010**, *210*, 193–199.

(S5) Um, I.-H.; Lee, J.-Y.; Ko, S.-H.; Bae, S.-K. *J. Org. Chem.* **2006**, *71*, 5800–5803.

(S6) Litwinienko, G.; Ingold, K. U. *J. Org. Chem*. **2004**, *69*, 5888–5896.
